# Supplementary material for: Characteristics and expression of lncRNA and transposable elements in Drosophila aneuploidy
Source: iScience. 2023 Nov 19;26(12):108494. doi: 10.1016/j.isci.2023.108494 (PMC10730892; doi:10.1016/j.isci.2023.108494)
Supplement: Document S1. Figures S1–S10 [file mmc1.pdf]

iScience, Volume *26*

Supplemental information

Characteristics and expression of lncRNA and transposable elements in *Drosophila* aneuploidy

Shuai Zhang, Ruixue Wang, Xilin Zhu, Ludan Zhang, Xinyu Liu, and Lin Sun

iScience, Volume *26*

Supplemental information

Characteristics and expression of lncRNA and transposable elements in *Drosophila* aneuploidy

Shuai Zhang, Ruixue Wang, Xilin Zhu, Ludan Zhang, Xinyu Liu, and Lin Sun

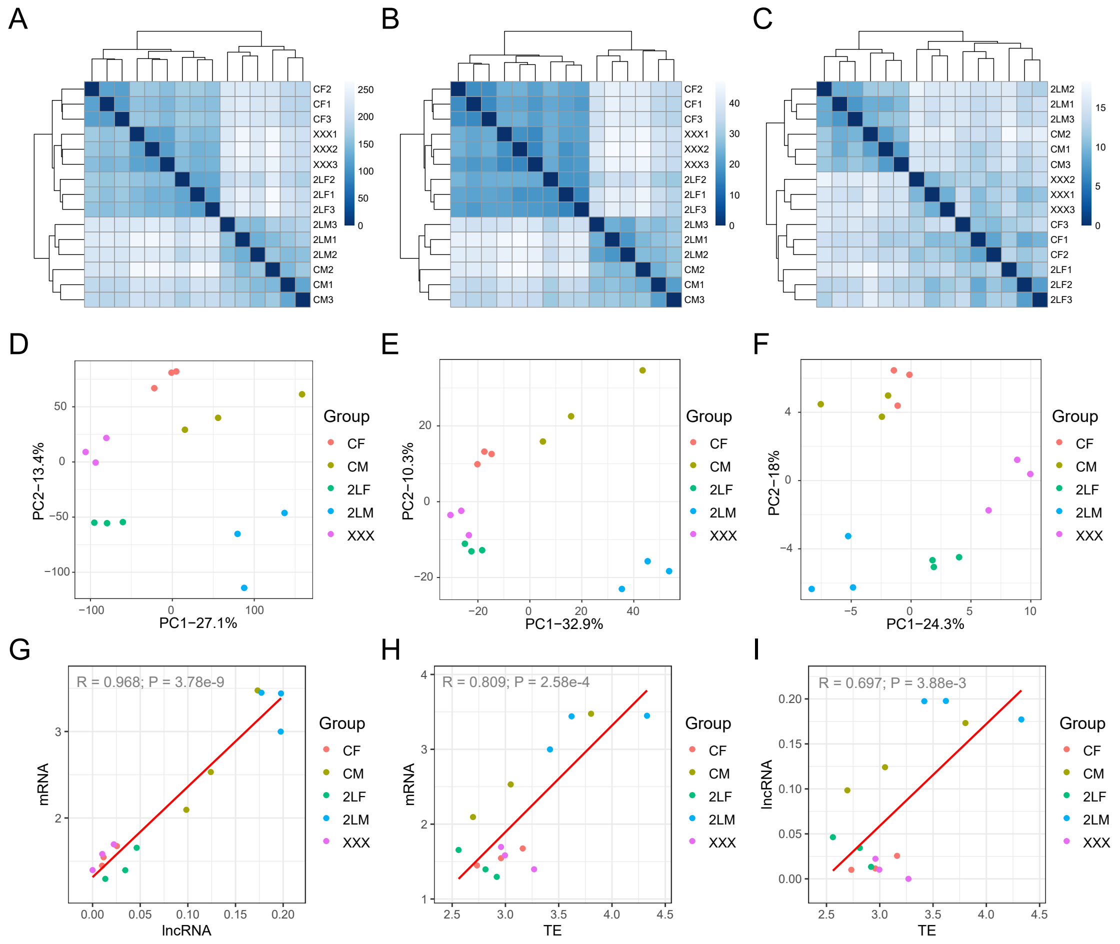

Figure S1, Related to Figure 1. Quality control of samples by different transcript types

(A-C) Heatmaps of distances between samples based on protein-coding genes (A), long noncoding RNAs (lncRNAs) (B), and transposable elements (TEs) (C).

(D-F) PCA plots of samples based on protein-coding genes (D), lncRNAs (E), and TEs (F).

(G-I) Correlation of the expression of different types of transcripts among samples. The Pearson's correlation coefficients and p-values of the medians of expression levels (CPM) for two types of transcripts among samples are shown in the upper left corner of the plots.

CF, wildtype female control; CM, wildtype male control; 2LF, trisomy 2L female; 2LM, trisomy 2L male; XXX, metafemale.

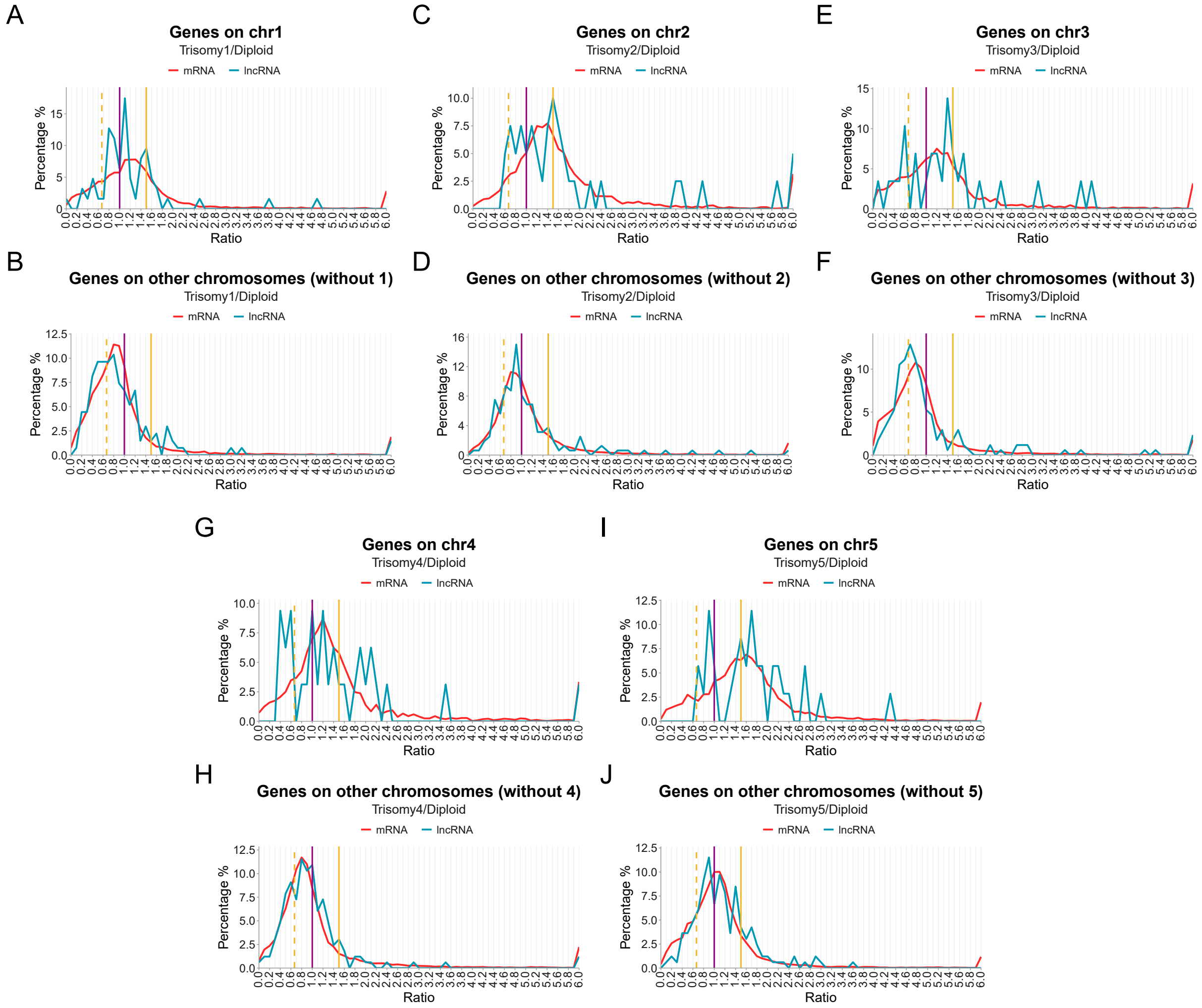

Figure S2, Related to Figure 2. Ratio Distributions of lncRNA and mRNA in aneuploid *Arabidopsis*

(A,B) Ratio distributions of the expression levels of lncRNA and mRNA on chromosome 1 (A) and other chromosomes (B) in trisomy 1 *Arabidopsis*.

(C,D) Ratio distributions of the expression levels of lncRNA and mRNA on chromosome 2 (C) and other chromosomes (D) in trisomy 2 *Arabidopsis*.

(E,F) Ratio distributions of the expression levels of lncRNA and mRNA on chromosome 3 (E) and other chromosomes (F) in trisomy 3 *Arabidopsis*.

(G,H) Ratio distributions of the expression levels of lncRNA and mRNA on chromosome 4 (G) and other chromosomes (H) in trisomy 4 *Arabidopsis*.

(I,J) Ratio distributions of the expression levels of lncRNA and mRNA on chromosome 5 (I) and other chromosomes (J) in trisomy 5 *Arabidopsis*.

The vertical purple solid line represents the ratio of 1.00, the vertical yellow solid line represents the ratio of 1.50, and the vertical yellow dashed line shows the ratio of 0.67. The ratio distributions were generated using ERCC-normalized RPKM data downloaded from GSE79676, and the percentages of frequencies were plotted in bins of 0.1.

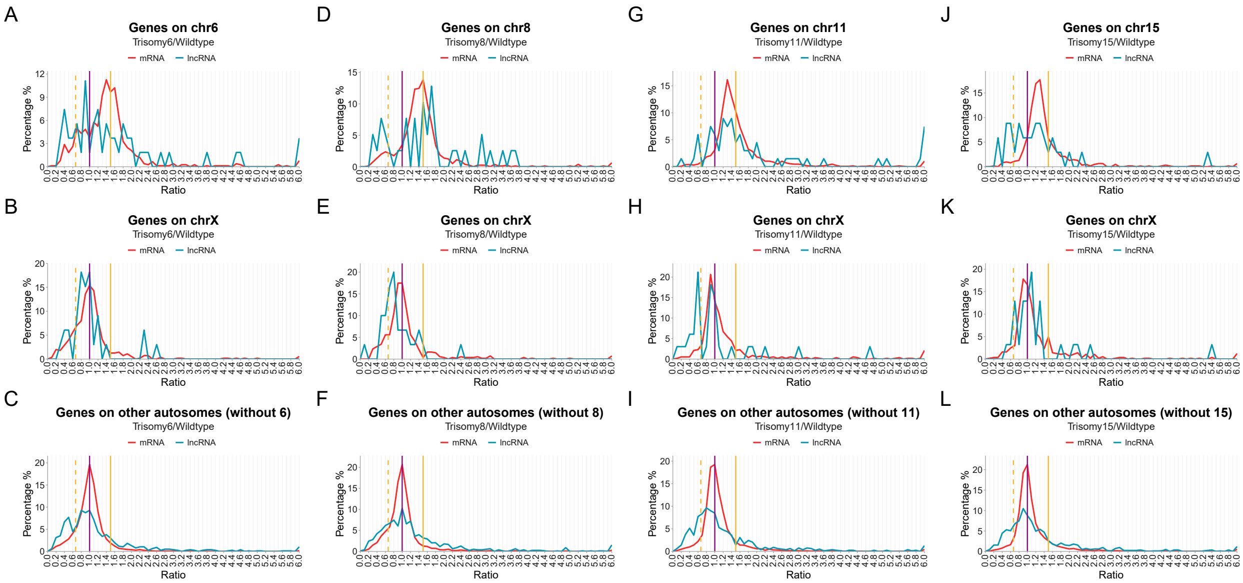

Figure S3, Related to Figure 2. Ratio Distributions of lncRNA and mRNA in aneuploid embryonic stem cells in mouse

(A-C) Ratio distributions of the expression levels of lncRNA and mRNA on chromosome 6 (A), chromosome X (B), and other autosomes (C) in trisomy 6 mouse embryonic stem cells.

(D-F) Ratio distributions of the expression levels of lncRNA and mRNA on chromosome 8 (D), chromosome X (E), and other autosomes (F) in trisomy 8 mouse embryonic stem cells.

(G-I) Ratio distributions of the expression levels of lncRNA and mRNA on chromosome 11 (G), chromosome X (H), and other autosomes (I) in trisomy 11 mouse embryonic stem cells.

(J-L) Ratio distributions of the expression levels of lncRNA and mRNA on chromosome 15 (J), chromosome X (K), and other autosomes (L) in trisomy 15 mouse embryonic stem cells.

The vertical purple solid line represents the ratio of 1.00, the vertical yellow solid line represents the ratio of 1.50, and the vertical yellow dashed line shows the ratio of 0.67. The ratio distributions were generated using CPM data downloaded from GSE179435, and the percentages of frequencies were plotted in bins of 0.1.

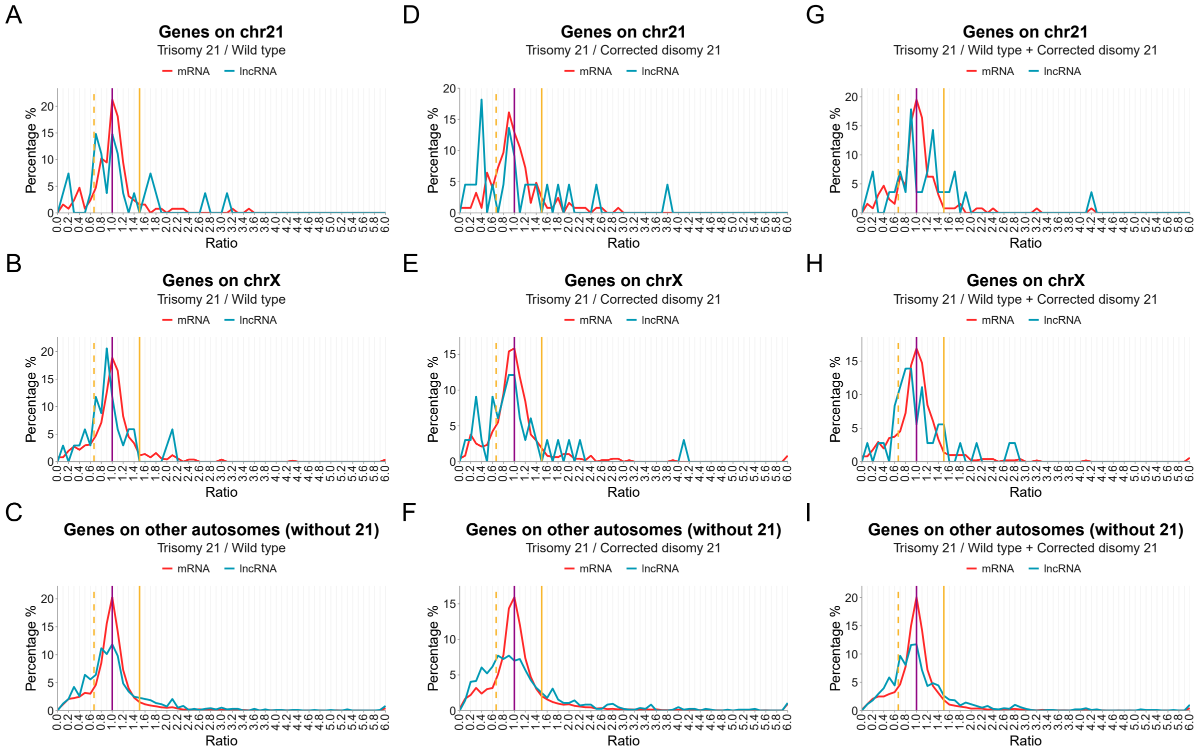

Figure S4, Related to Figure 2. Ratio Distributions of lncRNA and mRNA in induced-pluripotent stem-derived vascular endothelial cells (iPSC-derived ECs) in Down syndrome (human trisomy 21)

(A-C) Ratio distributions of the expression levels of lncRNA and mRNA on chromosome 21 (A), chromosome X (B), and other autosomes (C) in trisomy 21 iPSC-derived ECs compared with wildtype cells.

(D-F) Ratio distributions of the expression levels of lncRNA and mRNA on chromosome 21 (D), chromosome X (E), and other autosomes (F) in trisomy 21 iPSC-derived ECs compared with isogenic corrected disomy 21 cells.

(G-I) Ratio distributions of the expression levels of lncRNA and mRNA on chromosome 21 (G), chromosome X (H), and other autosomes (I) in trisomy 21 iPSC-derived ECs compared with wildtype and corrected disomy 21 cells.

The vertical purple solid line represents the ratio of 1.00, the vertical yellow solid line represents the ratio of 1.50, and the vertical yellow dashed line shows the ratio of 0.67. The ratio distributions were generated using FPKM data downloaded from GSE203257, and the percentages of frequencies were plotted in bins of 0.1.

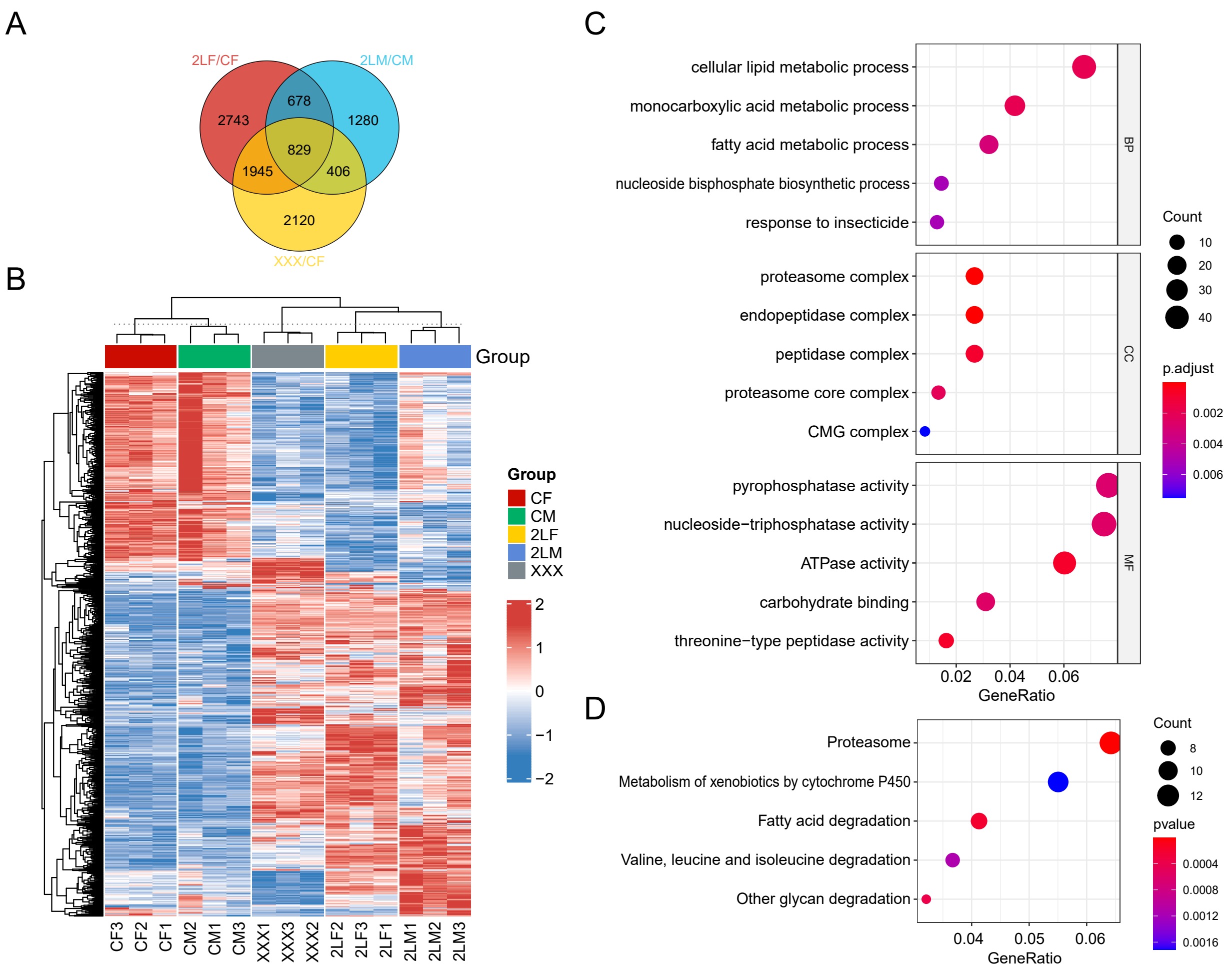

Figure S5, Related to Figure 3. Differential expression analysis of mRNA in aneuploid *Drosophila*

(A) Venn diagram of the number of differentially expressed mRNA (DE-mRNA) in trisomy 2L female, trisomy 2L male, and metafemale.

(B) Clustering heatmap of mRNAs differentially expressed in all three aneuploidies.

(C) Top 5 enriched GO terms in biological process (BP), cell component (CC), and molecular function (MF) of DE-mRNA in three aneuploidies.

(D) Top 5 enriched KEGG pathways of DE-mRNA in three aneuploidies.

CF, wildtype female control; CM, wildtype male control; 2LF, trisomy 2L female; 2LM, trisomy 2L male; XXX, metafemale.

A

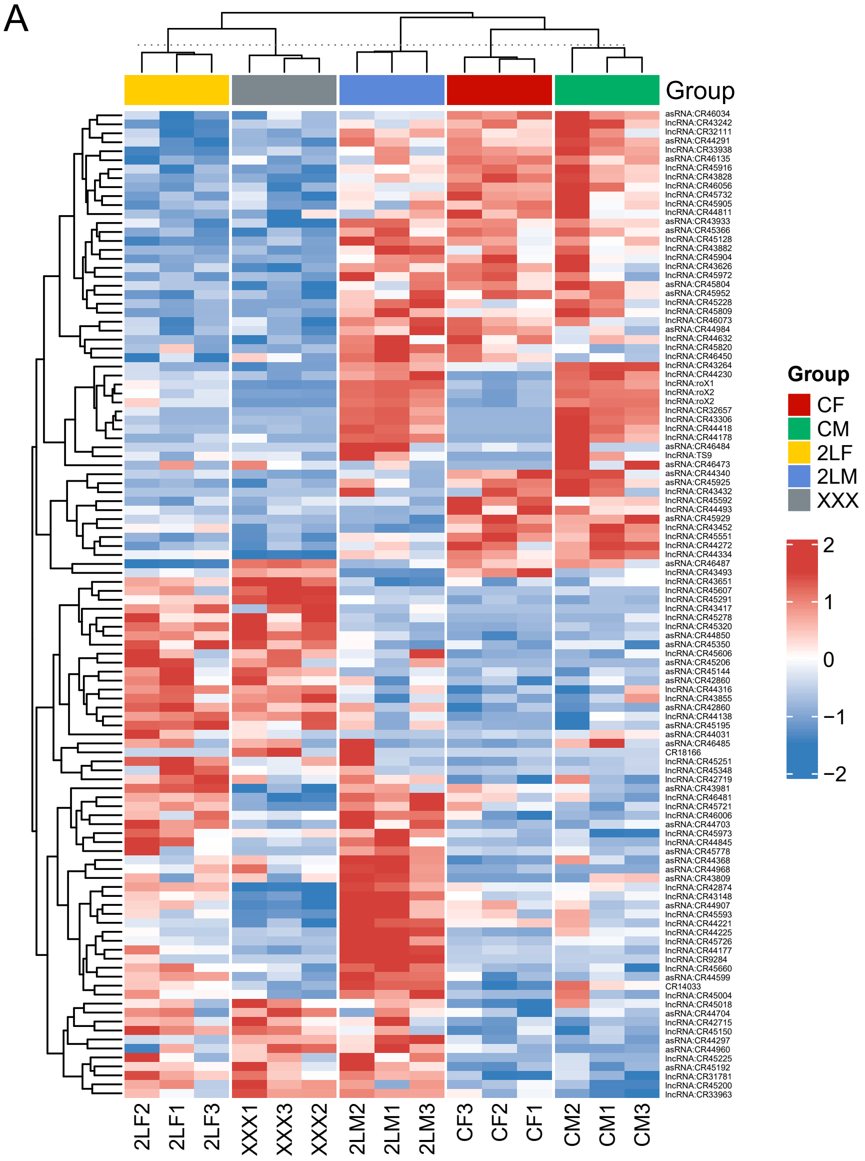

Figure S6, Related to Figure 3. LncRNAs that are significantly differentially expressed in at least two groups of comparisons

(A) Clustering heatmap of lncRNAs differentially expressed in at least two groups of comparisons of trisomy 2L female, trisomy 2L male, and metafemale to their respective controls. CF, wildtype female control; CM, wildtype male control; 2LF, trisomy 2L female; 2LM, trisomy 2L male; XXX, metafemale.

A

lncRNA	predicted functions
lncRNA:roX2	oxidative phosphorylation; mitophagy; biosynthesis of amino acids; peroxisome
lncRNA:CR43306	inorganic ion homeostasis; aerobic respiration; cilium assembly; cilium movement; sperm axoneme assembly; organelle assembly; axoneme assembly; plasma membrane bounded cell projection assembly; mitophagy; biosynthesis of amino acids; peroxisome
lncRNA:CR44178	metal ion homeostasis; aerobic respiration; cilium assembly; cilium movement; organelle assembly; mitophagy; biosynthesis of amino acids
lncRNA:CR32657	inorganic ion homeostasis; aerobic respiration; cilium assembly; cilium movement; sperm axoneme assembly; organelle assembly; mitophagy; biosynthesis of amino acids; peroxisome
lncRNA:CR33938	ubiquitin-dependent protein catabolic process; proteasome assembly; cell fate specification; leg disc proximal/distal pattern formation; renal tubule development; Malpighian tubule development; transcription initiation from RNA polymerase II promoter; histone H4 acetylation; cellular response to virus; regulation of smoothened signaling pathway; proteasome; protein processing in endoplasmic reticulum
lncRNA:roX1	oxidative phosphorylation; mitophagy; biosynthesis of amino acids; peroxisome
lncRNA:CR44418	mitophagy; biosynthesis of amino acids
lncRNA:CR43828	lysosome
lncRNA:CR44225	regulation of actin polymerization or depolymerization; receptor metabolic process; supramolecular fiber organization; glutamine family amino acid biosynthetic process; receptor clustering
lncRNA:CR45916	glycerophospholipid metabolism
lncRNA:CR9284	regulation of hydrolase activity; tyrosine metabolic process; apoptotic mitochondrial changes
asRNA:CR44850	humoral immune response; positive regulation of antibacterial peptide biosynthetic process; positive regulation of Toll signaling pathway; negative regulation of endopeptidase activity; negative regulation of proteolysis; mitochondrial electron transport, cytochrome c to oxygen
lncRNA:CR45809	attachment of spindle microtubules to kinetochore; regulation of cell cycle; protein phosphorylation; negative regulation of BMP signaling pathway; membrane organization; sperm axoneme assembly; male gamete generation

B

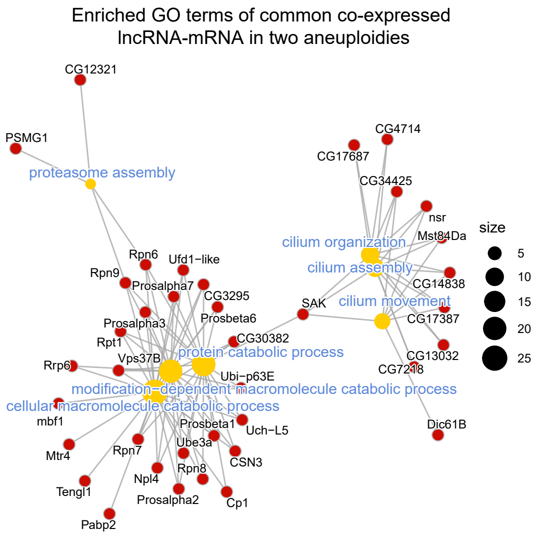

C

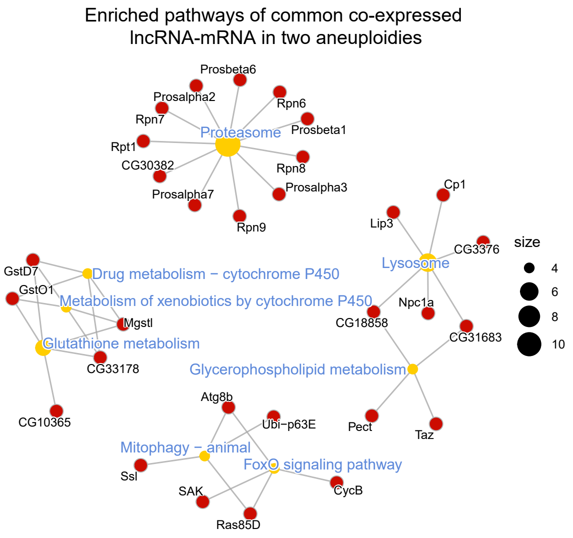

Figure S7, Related to Figure 4. The function of lncRNAs predicted based on lncRNA-mRNA co-expression network

(A) The predicted functions of several lncRNAs with high degree according to the enrichment analysis of their co-expressed mRNAs.

(B) Enriched functions of lncRNA-mRNA co-expression network and related genes.

(C) Enriched pathways of lncRNA-mRNA co-expression network and related genes.

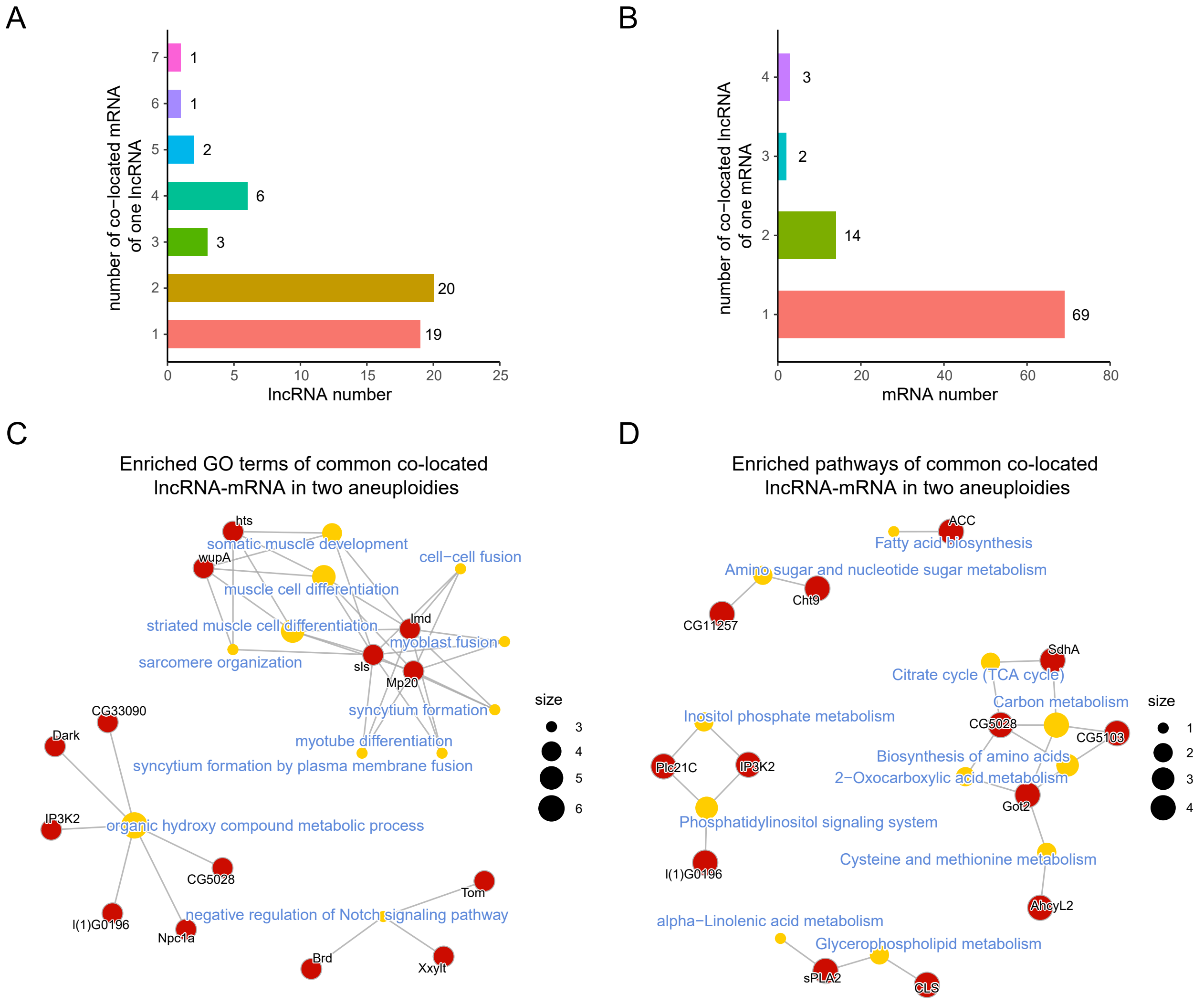

Figure S8, Related to Figure 4. Co-located lncRNA and mRNA, and their enrichment analysis

(A) The number of co-located mRNA owned by per lncRNA.

(B) The number of co-located lncRNA owned by per mRNA.

(C) Enriched functions of lncRNA-mRNA co-localization network and related genes.

(D) Enriched pathways of lncRNA-mRNA co-localization network and related genes.

A

TE	predicted functions
HMS-Beagle	ubiquitin-dependent protein catabolic process; proteasome assembly; response to heat; response to external biotic stimulus; Hippo signaling pathway; apoptosis
accord	organophosphate metabolic process; regulation of ATP metabolic process; purine nucleotide biosynthetic process; oxidative phosphorylation; drug metabolism
Burdock	cellular metal ion homeostasis; regulation of lipid storage; organic acid transmembrane transport
Juan	purine ribonucleoside monophosphate biosynthetic process; ocellus pigment metabolic process; biosynthesis of cofactors
Max	negative regulation of peptide secretion; regulation of calcium-mediated signaling; cellular response to starvation; response to carbohydrate; oxidative phosphorylation; drug metabolism; phagosome

B

Enriched GO terms of common co-expressed TE-mRNA in two aneuploidies

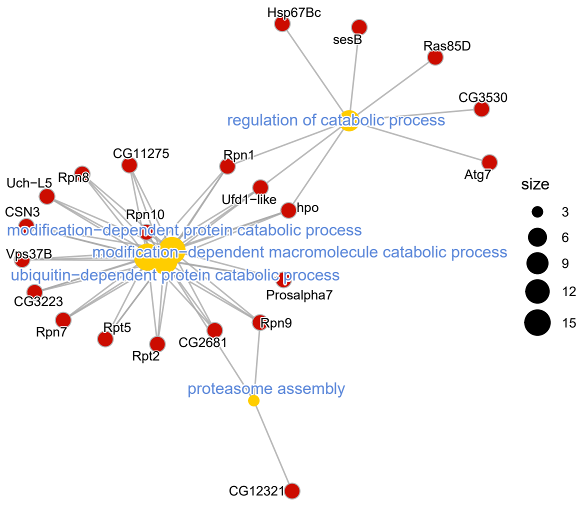

C

Enriched pathways of common co-expressed TE-mRNA in two aneuploidies

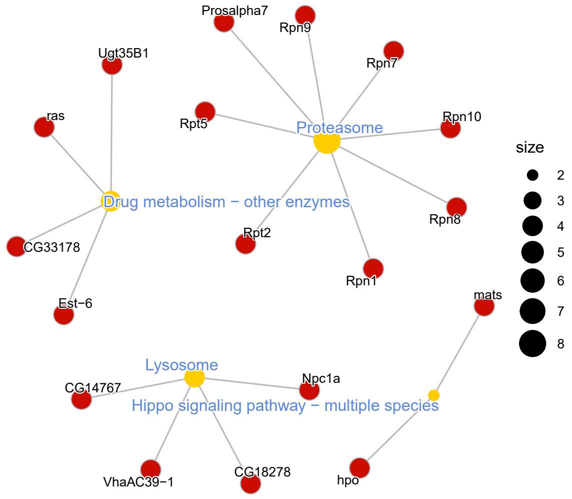

Figure S9, Related to Figure 5. The function of TE families predicted based on TE-mRNA co-expression network

(A) The predicted functions of several TE families with high degree according to the enrichment analysis of their co-expressed mRNAs.

(B) Enriched functions of TE-mRNA co-expression network and related genes.

(C) Enriched pathways of TE-mRNA co-expression network and related genes.

A

Primer name	Sequence
CG3295-F-T3	TGTTGGGAAATCACTCCCAATTAACTGGAGCTAACAATGGACAG
CG3295-R-T7	GTAATACGACTCACTATAGGGAGACCACAGTAGGGGCACTTCAGTATG
HmgD-F-T3	TGTTGGGAAATCACTCCCAATTAACCACCCTAATCCATCACCAA
HmgD-R-T7	GTAATACGACTCACTATAGGGAGACCACCAACGCACATCCTGCTAATC
tsh-F-T3	TGTTGGGAAATCACTCCCAATTAACTCCGAAAGCATAGTTCAGG
tsh-R-T7	GTAATACGACTCACTATAGGGAGACCACCAACCTTGTCTGAGGACTTG
dap-F-T3	TGTTGGGAAATCACTCCCAATTAAGACCGCAAGCTAAATGGAAT
dap-R-T7	GTAATACGACTCACTATAGGGAGACCACAAGTAATGCGGATGTGTTGG
Tehao-F-T3	TGTTGGGAAATCACTCCCAATTAATTGCGTCTGAACAAGAACTC
Tehao-R-T7	GTAATACGACTCACTATAGGGAGACCACGTAGGTCTTGTCCTTGTCCA
Su(var)205-F-T3	TGTTGGGAAATCACTCCCAATTAATCAAAACGTACCACAGATGC
Su(var)205-R-T7	GTAATACGACTCACTATAGGGAGACCACTACAGACACTGACTCTGGAC
ifc-F-T3	TGTTGGGAAATCACTCCCAATTAATTATTAACCCGAAGCCACCT
ifc-R-T7	GTAATACGACTCACTATAGGGAGACCACGTCCGAATGCCCCTTATCTA
nonA-l-F-T3	TGTTGGGAAATCACTCCCAATTAAGACGCAACCGCTTATATGTT
nonA-l-R-T7	GTAATACGACTCACTATAGGGAGACCACCGTCGCTATTATCTGGACCT
Atg8b-F-T3	TGTTGGGAAATCACTCCCAATTAAGCAGCTACATAAGACCTTCG
Atg8b-R-T7	GTAATACGACTCACTATAGGGAGACCACTAGTCCTTGTCGAAGTGCTC
lncRNA:CR33938-F-T3	TGTTGGGAAATCACTCCCAATTAAAAAACGTATGGACTCGGCTA
lncRNA:CR33938-R-T7	GTAATACGACTCACTATAGGGAGACCACAAATTTTGTCCAAAGGGCCG
lncRNA:CR45916-F-T3	TGTTGGGAAATCACTCCCAATTAAAATGCGATGGGAGTAAGAGT
lncRNA:CR45916-R-T7	GTAATACGACTCACTATAGGGAGACCACAGGGGGTAAAACAACTCCAT
asRNA:CR42860-F-T3	TGTTGGGAAATCACTCCCAATTAAGATGACTGAACAGCCGTAAC
asRNA:CR42860-R-T7	GTAATACGACTCACTATAGGGAGACCACAGGCATACGTGGATGGTTTA
lncRNA:CR43148-F-T3	TGTTGGGAAATCACTCCCAATTAAAAACTGTAAGGAAGGTGGCT
lncRNA:CR43148-R-T7	GTAATACGACTCACTATAGGGAGACCACGGCTTGTAGGAGTGGAAATC
lncRNA:CR44178-F-T3	TGTTGGGAAATCACTCCCAATTAAAGGGTATTGAAAGAGGGGTG
lncRNA:CR44178-R-T7	GTAATACGACTCACTATAGGGAGACCACGTCAACAGTTTCCACGTCTT
lncRNA:CR44418-F-T3	TGTTGGGAAATCACTCCCAATTAAAGTTGGAACAGGACGAGTAA
lncRNA:CR44418-R-T7	GTAATACGACTCACTATAGGGAGACCACTATTTGCCCGAAGTCAACTC
HMS-Beagle-F-T3	TGTTGGGAAATCACTCCCAATTAAGAATGTAGAGTGGGGAGTGG
HMS-Beagle-R-T7	GTAATACGACTCACTATAGGGAGACCACTATTGATGGAGAAGGCGGTC

B

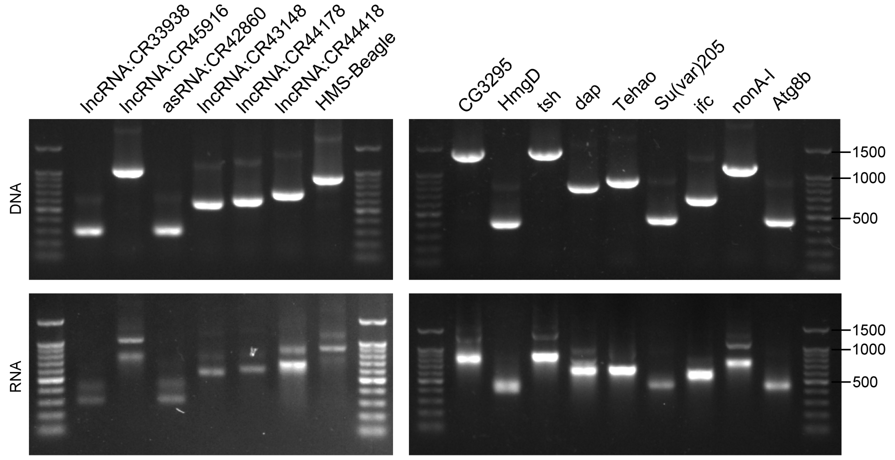

Figure S10, Related to Figure 6. Probe primers designed for candidate genes and their validation

(A) The sequences of probe primers.

(B) The size of the products of the primers after PCR amplification and *in vitro* transcription was tested by agarose gel electrophoresis.
