# Supplementary material for: Characteristics and expression of lncRNA and transposable elements in Drosophila aneuploidy
Source: iScience. 2023 Nov 19;26(12):108494. doi: 10.1016/j.isci.2023.108494 (PMC10730892; doi:10.1016/j.isci.2023.108494)
Supplement: Data S1. Embryo-FISH of sixteen candidate genes, related to Figure 6 [file mmc2.pdf]

iScience

CellPress
OPEN ACCESS

Article

Characteristics and expression of lncRNA and transposable elements in *Drosophila* aneuploidy

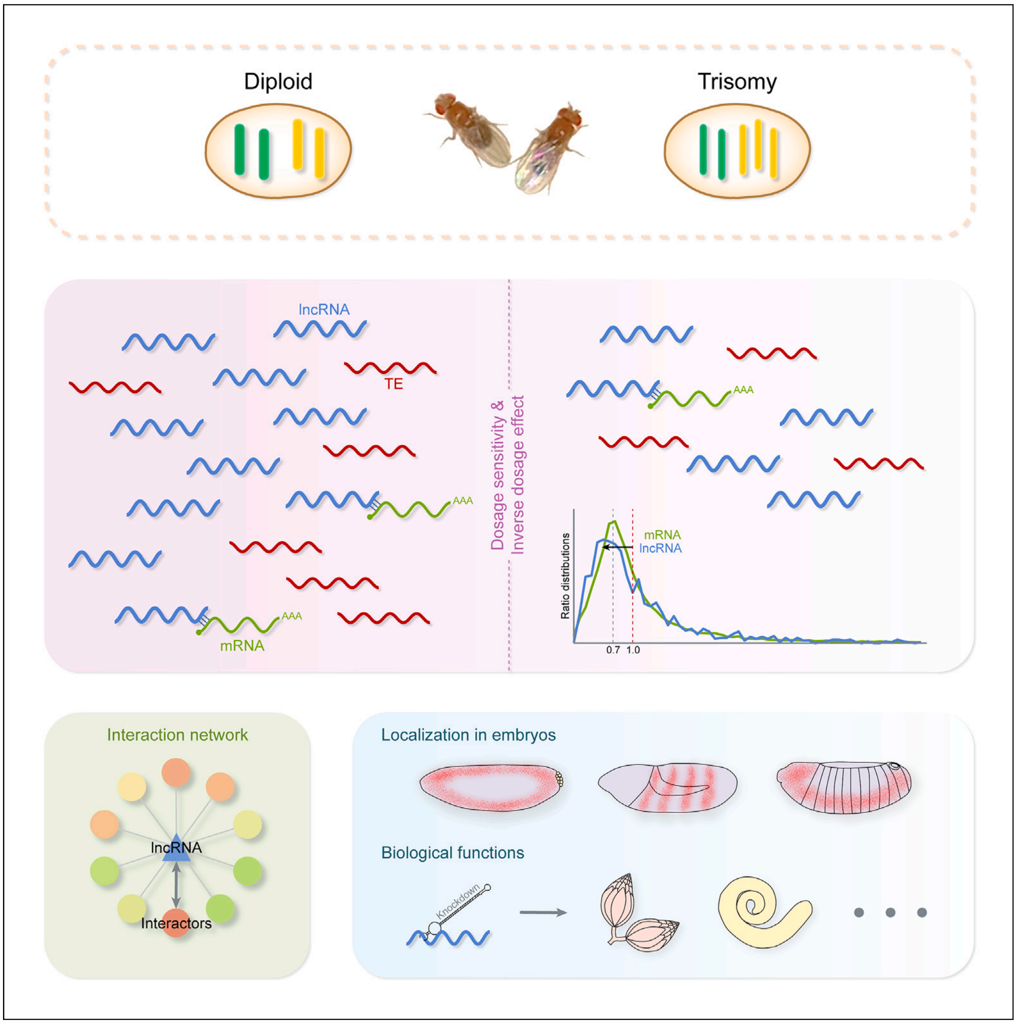

Shuai Zhang, Ruixue Wang, Xilin Zhu, Ludan Zhang, Xinyu Liu, Lin Sun

sunlin@bnu.edu.cn

Highlights

We showed the characteristics and expression of lncRNAs and TEs in aneuploidy

LncRNAs and TEs are dosage affected and strongly responsive to inverse dosage effect

Noncoding genes can act together with their co-expressed/co-localized genes

Some lncRNAs and TEs have developmental and transcriptional regulatory functions

Zhang et al., iScience 26, 108494
December 15, 2023 © 2023 The Authors.
https://doi.org/10.1016/j.isci.2023.108494

iScience

CellPress
OPEN ACCESS

Article

Characteristics and expression of lncRNA and transposable elements in *Drosophila* aneuploidy

Shuai Zhang,[1,3] Ruixue Wang,[1,3] Xilin Zhu,[1] Ludan Zhang,[1] Xinyu Liu,[1] and Lin Sun[1,2,4,*]

SUMMARY

Aneuploidy can globally affect the expression of the whole genome, which is detrimental to organisms. Dosage-sensitive regulators usually have multiple intermolecular interactions, and changes in their stoichiometry are responsible for the dysregulation of the regulatory network. Currently, studies on noncoding genes in aneuploidy are relatively rare. We studied the characteristics and expression profiles of long noncoding RNAs (lncRNAs) and transposable elements (TEs) in aneuploid *Drosophila*. It is found that lncRNAs and TEs are affected by genomic imbalance and appear to be more sensitive to an inverse dosage effect than mRNAs. Several dosage-sensitive lncRNAs and TEs were detected for their expression patterns during embryogenesis, and their biological functions in the ovary and testes were investigated using tissue-specific RNAi. This study advances our understanding of the noncoding sequences in imbalanced genomes and provides a novel perspective for the study of aneuploidy-related human diseases such as cancer.

INTRODUCTION

Aneuploidy usually causes serious harm to organisms, including defects in cell proliferation, altered metabolic characteristics, abnormal protein production, increased genomic instability, and decreased cell viability.[1–4] This results in growth retardation, developmental delays and defects, as well as abnormal morphology of organisms.[1,2,5–7] Aneuploidy is also frequently present in human cancers.[1,5] Studies have found that the gene expression in aneuploidy shows a wide range of dysregulation.[8–10] Not only is the expression of genes located on varied chromosomes (*cis*) regulated, but the genes in the remainder of the genome (*trans*) also have altered expression,[10–13] and the influence can affect the entire gene expression regulatory network.[14,15] The molecular basis of genomic imbalance caused by dosage changes of single chromosomes or chromosome segments is related to the disturbance of the balanced relationship of regulatory genes.[16,17] Changes in the stoichiometry of subunits of macromolecular complexes, mainly including transcription factors, chromatin proteins, and signal transduction components,[18] will alter their assembly kinetics and overall function.[19,20] This view, namely, the Gene Balance Hypothesis, has been confirmed by evolutionary genomics, quantitative trait genetics, and other evidences. It can explain, to some extent, the phenomena of aneuploid syndromes, dosage compensation, and regulatory gene evolution following polyploidization.[21–23] Currently, most of the gene expression studies in aneuploidy are conducted for protein-coding genes, while the modulation of noncoding genes has rarely been studied.[24,25]

Long noncoding RNAs (lncRNAs) are defined as RNAs that are longer than 200 nucleotides and lack a significant open reading frame.[26] LncRNAs are mainly transcribed by RNA polymerase II similarly to mRNAs (some can also be transcribed by RNA polymerase III). Pre-mature lncRNAs usually have 5′-end m7G caps and 3′-end poly(A) tails and undergo alternative splicing. LncRNAs have high diversity and low evolutionary conservation, but they usually have conserved secondary structures, which may be crucial to their functions.[27,28] Although most lncRNAs have not been well characterized and are still functionally unknown, there are sufficient evidences that they have important cellular functions.[28–30] Some lncRNAs can participate in modulations at epigenetic, transcriptional, and post-transcriptional levels through different mechanisms, and many lncRNAs are regulated by other genes.[27,28] Functional lncRNA transcripts can serve as signals, decoys, guides, and scaffolds to exert their *cis* or *trans* molecular functions.[31] In other cases, the transcription or splicing process of lncRNAs will have a transcription regulation function. Furthermore, the regulatory function may depend only on the DNA elements embedded in lncRNA loci and not on transcriptional products.[32–34] Despite the relatively low expression levels, lncRNAs usually tend to be expressed in certain tissues, cell types, and developmental stages[35,36] and are closely associated with histone modification, chromatin remodeling, genomic imprinting, dosage compensation, subcellular structure organization, embryogenesis, as well as a variety of human diseases, including cancer.[37–42]

Another genomic component that is frequently overlooked is the transposable elements (TEs).[43] Almost all eukaryotes contain TEs, with variable abundance and diversity among species.[43] There are thousands of TE families in plants, and in some species, TEs constitute more

[1]Beijing Key Laboratory of Gene Resource and Molecular Development, College of Life Sciences, Beijing Normal University, Beijing 100875, China
[2]Key Laboratory of Cell Proliferation and Regulation Biology of Ministry of Education, College of Life Science, Beijing Normal University, Beijing 100875, China
[3]These authors contributed equally
[4]Lead contact
*Correspondence: sunlin@bnu.edu.cn
https://doi.org/10.1016/j.isci.2023.108494

This is an open access article under the CC BY license (http://creativecommons.org/licenses/by/4.0/).

than 80% of the total genome (e.g., maize).[44] In contrast, TEs account for about 45% of the human genome and 20% in *Drosophila*.[45,46] The insertion of TEs is often detrimental, which can lead to various human inherited diseases and is related to tumorigenesis.[47–49] The main factors causing these harmful effects are: changes in gene expression, cytotoxicity of TE transcripts or protein products, and ectopic recombination caused by TEs.[50] TE-mediated genome shuffling can also be detrimental, such as the retrotransposon *LINE-1* (*L1*) in human and *Foldback* (*FB*) element in *Drosophila*.[51–53] TEs can play a regulatory role in different ways. In addition to directly changing the gene structures or destroying *cis*-regulatory elements,[46,54] they can also carry TE-derived DNA elements such as promoters, enhancers, and insulators into the regulatory apparatus.[47,55,56] Another mechanism is to spread their repressive epigenetic marks to adjacent chromatin and silence nearby genes.[46,57] Moreover, TEs also contribute a large number of noncoding regulatory RNAs, including lncRNAs, microRNAs (miRNAs), and circular RNAs (circRNAs).[47,58,59] A study found that approximately 75% of human lncRNAs contained TE-derived fragments,[58] and another study showed that 12% of human miRNA genes were derived from TEs.[59] TEs are essential in gene regulatory networks, embryonic development, dosage compensation, domestication of animals and plants, etc.[47,60–62]

In previous studies, we investigated the global expression of autosomal and sex chromosome aneuploidy in *Drosophila*. Dosage compensation is found to occur in genes on the varied chromosomes, and inverse dosage effect, which is negatively related to the changes in chromosome numbers, is observed in genes located on unvaried chromosomes.[12,13,63,64] Some protein-coding genes have been identified as potential dosage-sensitive regulators in *Drosophila*.[18,63,64] A recent study on the dosage sensitivity of miRNAs in maize[25] inspired us to consider the responses of genes other than protein-coding genes such as lncRNAs and TEs in genomic imbalance. By analyzing the transcriptomes of autosomal and sex chromosome aneuploid, we describe the characteristics and expression profiles of lncRNAs and TEs in *Drosophila*. Given that both lncRNAs and TEs have important regulatory functions and are related to the growth, development, and disease of organisms, we asked whether lncRNAs and TEs also play roles in the modulation of genomic imbalance and the development of aneuploidy. We searched for dosage-sensitive lncRNAs and TEs, as well as their potential interactors; then we examined the expression patterns of candidate genes during embryogenesis in aneuploid *Drosophila*. These studies demonstrate the possibility of lncRNAs and TEs acting as dosage-sensitive regulators in gene expression and development in *Drosophila*.

RESULTS

Genomic characteristics of lncRNAs and TEs in aneuploid *Drosophila*

To investigate the expression and characteristics of noncoding sequences in aneuploid *Drosophila*, ribosomal RNA is removed from the samples to retain mRNA and all ncRNA except for rRNA. We also used strand-specific RNA sequencing to improve the accuracy of read mapping and transcript quantification. Through transcriptome analysis, we obtained the expression of more than 30,000 *Drosophila* transcripts in three biological replicates of five different karyotypes. Based on the known lncRNAs defined by FlyBase, these transcripts can be divided into 28,944 protein-coding transcripts and 2,952 lncRNA transcripts. In addition, according to Ensembl annotation, 5,574 TE insertions were found in the *Drosophila* genome. We combined them into 127 TE families and counted the overall expression of each TE family. Through the heatmaps and PCA plots of 15 samples, it was found that the three biological replicates of the same genotype clustered well, which represents the reproducibility of the samples and the high quality of sequencing (Figures S1A–S1F). Samples of the same sex were preferentially clustered together, whether they were aneuploid or not (Figures S1A–S1C). Furthermore, the expression levels of lncRNAs and mRNAs show a high correlation among different samples (Pearson's correlation coefficient = 0.968 and p value <0.05; Figure S1G). The correlation between TE and lncRNA/mRNA is low but still significantly positively correlated (Figure S1H, and S1I).

According to genomic positions, lncRNAs were divided into four types: long intergenic noncoding RNA (lincRNA, 56.4%), intronic-lncRNA (17.1%), sense-lncRNA (4.3%), and antisense-lncRNA (22.2%) (Figure 1A). Among them, lincRNA has the highest proportion, accounting for approximately half of all lncRNAs, which is consistent with previous studies[65,66] Benefiting from the strand-specific sequencing, which can retain the directional information of transcripts, the proportion of lncRNAs from antisense strands also accounts for more than 20%. The number of mRNA transcripts on each chromosome is much greater than that of lncRNAs, and the number of transcripts is approximately related to chromosome length (Pearson correlation coefficients: lncRNA = 0.932, mRNA = 0.992; p values: lncRNA = 2.20e-3, mRNA = 1.16e-5) (Figure 1B). By analyzing the number of exons and the length of transcripts from protein-coding genes and noncoding genes, it can be found that both the distributions of exon numbers and transcript lengths of lncRNAs are more concentrated than those of mRNAs, but the horizontal axis positions of lncRNA peaks are smaller (Figures 1C and 1D). As found in a previous study, most of the lncRNAs in *Drosophila* are monoexonic with an average size of only 1000 nt.[65] Moreover, we found that the average exon number and size of lncRNAs that were differentially expressed in at least one aneuploid (see in the following text) (1.669 exons and 1443.4 nt) were greater than those of lncRNAs that were not differentially expressed in any aneuploid (1.626 exons and 1016.0 nt). Previous reports suggested that developmentally dynamic lncRNAs are usually longer in length and contain more exons.[65,67] Therefore, we speculate that the lncRNAs affected by aneuploidy may share some common features with developmentally dynamic lncRNAs. The overall expression level of mRNA is significantly higher than that of lncRNA (Mann-Whitney U test p value <2.2e-16; Figure 1E), and the mean CPM values of mRNA are approximately three times higher than those of lncRNA (Figure 1F). The expression levels of lncRNA fluctuated relatively widely among genotypes and are lower in aneuploidies than in the corresponding euploidies. This indicates that lncRNAs may be modulated by a dosage-dependent inverse effect in aneuploidy.

TEs were also divided into four types, with intergenic-TEs (50.5%) and intronic-TEs (42%) being the two most abundant, which together account for more than 90% of all TEs (Figure 1G). The other two types, sense-TE (3.7%) and antisense-TE (3.7%), account for less than 10% of the total. The intronic-TEs account for a high proportion, which may be related to the fact that TE can introduce splicing sites into introns, leading to the generation of chimeric transcripts.[68] TEs are widely distributed on the autosomal and sex chromosomes of *Drosophila*, and it is

CellPress
OPEN ACCESS

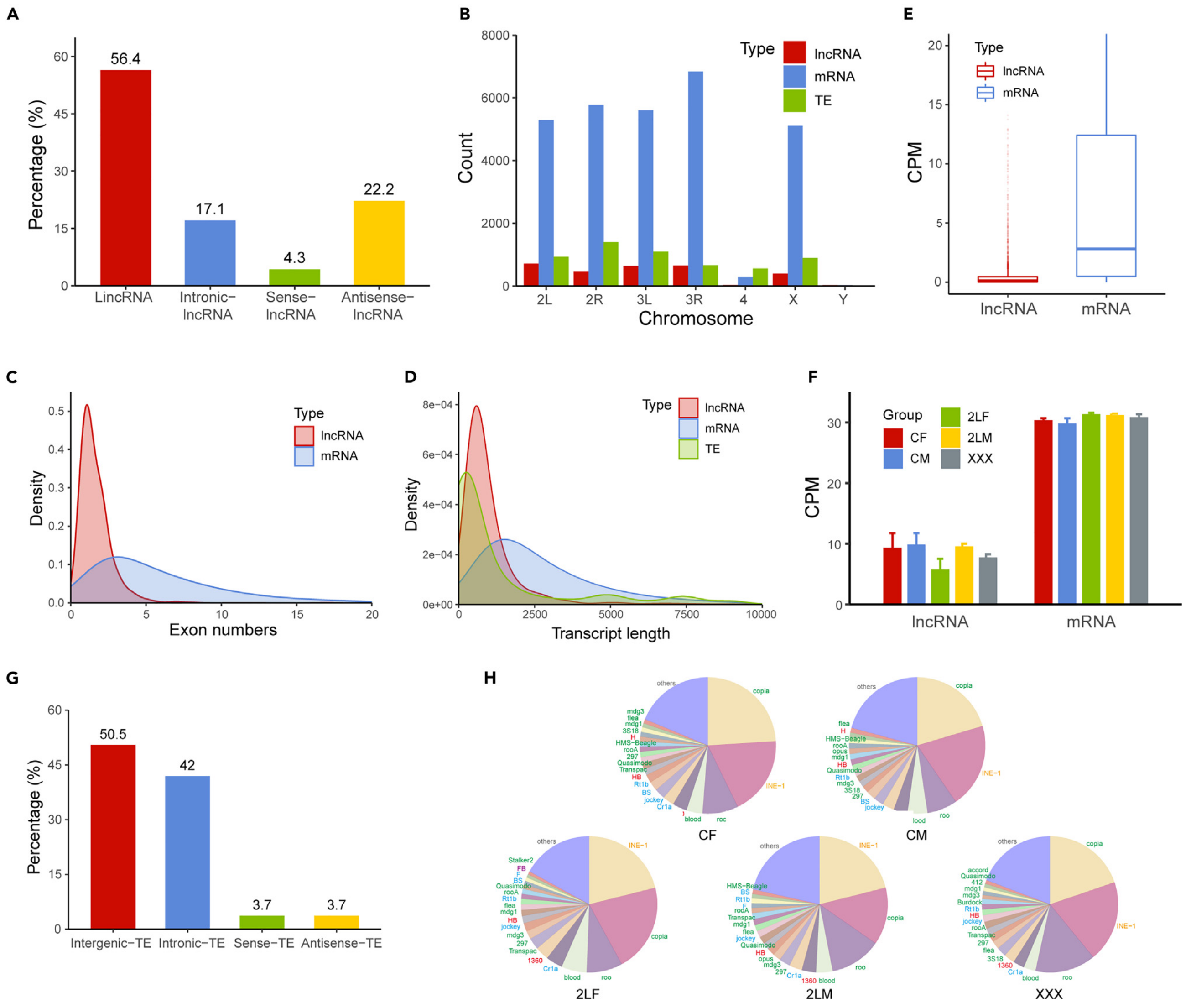

Figure 1. Characteristics of long noncoding RNA (lncRNA) and transposable elements (TEs) in *Drosophila* samples
(A) Percentage of different types of lncRNAs in *Drosophila*.
(B) The number of lncRNA, mRNA, and TE insertion located on different chromosomes.
(C) Density plot of exon numbers of lncRNA and mRNA.
(D) Density plot of transcript length of lncRNA, mRNA, and TE.
(E) Boxplots of the expression levels (CPM) of lncRNA and mRNA.
(F) Mean expressions of lncRNA and mRNA in each sample. The error bars indicate standard deviation (SD) of three biological replicates.
(G) Percentage of different types of TEs in *Drosophila*.
(H) Pie charts showing the proportion of the expression of each TE family to the total expression of TEs in each sample. The text colors of the top 20 TE families listed represented their classification, while the green text represented LTR (long terminal repeat) retrotransposons, blue represented LINE-like TEs, yellow represented SINE-like TEs, and red represented TIR (terminal inverted repeat) DNA transposons. CF, wild-type female control; CM, wild-type male control; 2LF, trisomy 2L female; 2LM, trisomy 2L male; XXX, metafemale.

noteworthy that there is an excessive amount of TEs relative to chromosome length on the fourth chromosome, even more than the number of mRNAs (Figure 1B). This is because many TEs tend to accumulate in heterochromatic regions with low recombination rates, and the fourth chromosome is a specific transposon-dense chromosome.[43,69] The peak position of the transcript length distribution of TEs is lower than mRNAs and lncRNAs, but there are two small peaks at larger values, indicating that there is a polarization in the size of TEs (Figure 1D). The total expression of TEs in each sample accounts for 0.2%–0.3% of all mapped reads. Analysis of the TE families with the highest expression in each genotype reveals a similar picture (Figure 1H). *Copia*, *INE-1*, and *roo*, the top 3 highly expressed TE families are all retrotransposons, and their expression accounts for approximately half of all TEs. More than half of the TE families with the top 20 levels of expression are LTR

(long terminal repeat) retrotransposons, the second most abundant type is LINE-like TE, and a few TIR (terminal inverted repeat) DNA transposons represented by the 1,360 family are also highly expressed (Figure 1H).

There are dosage sensitivities of lncRNAs and TEs in aneuploidy

Previous studies have found that the global expression in *Drosophila* and other species is affected by genomic imbalance. The genes located on the varied chromosomes (*cis*) generally show dosage compensation, while the genes on unvaried chromosomes (*trans*) are mainly subject to negative modulation with chromosome number changes, which is called an inverse dosage effect.[12,13,64] To determine whether lncRNAs and TEs are regulated by dosage-dependent effects, we generated ratio distributions of expression changes for different types of transcripts in aneuploidies compared with diploid controls according to their chromosomal locations (Figure 2).

In trisomy chromosome 2 left arm (2L) females, the ratio distribution curves of X-linked lncRNAs and mRNAs are inversely regulated by the addition of an autosomal arm and concentrated at 0.67, as indicated by the vertical yellow dashed line, and there is no significant difference between lncRNAs and mRNAs (Figure 2A; Kolmogorov-Smirnov (K-S) test p value = 0.66). In contrast, the ratio distribution of mRNAs expressed from chromosome 2L is centered around the ratio of 1.0, as indicated by the vertical purple solid line, representing dosage compensation (Figure 2B). However, the ratio distribution of lncRNAs expressed from 2L is significantly different from that of mRNAs (K-S test p value = 4.4e-3). Although there is a high distribution around 1.0, the highest peak appears at a ratio of 0.7 (Figure 2B). This result indicates that most of the lncRNAs expressed from the varied chromosome can be further inversely regulated. At the same time, the distribution of lncRNAs also has a small peak at 1.5 indicated by the vertical yellow solid line, which represents lncRNAs that exhibit a proportional dosage effect without compensation. For lncRNAs and mRNAs expressed from other autosomes, the ratio distribution curves both shift to the left, with the peak of mRNA at 0.6 and the peak of lncRNA at 0.4 (Figure 2C; K-S test p value = 7.5e-6). Therefore, the *trans* lncRNAs and mRNAs, which are located on the unvaried chromosomes both exhibit an inverse dosage effect, but lncRNAs are affected to a greater extent.

The aneuploid effect in trisomy 2L males is different from that in females. The peak of the distributions of X-linked lncRNAs and mRNAs are both centered around the ratio of 1.0 (Figure 2D; K-S test p value = 0.71), indicating no changes in expression. The peak of the ratio distribution curve of mRNAs expressed from 2L is slightly lower than 1.5 (Figure 2E), which indicates that the expression level of mRNA is compensated to some extent but not completely. The distribution of 2L lncRNAs is significantly different from that of mRNA (K-S test p value = 4.2e-7), with one peak near the ratio of 1.1, representing dosage compensation, and two secondary peaks near 0.7 (inverse dosage effect) and 1.5 (gene dosage effect). The peak of mRNA localized on other autosomes is at the ratio of 0.9 (Figure 2F), representing the slight *trans*-acting inverse dosage effect. The peak of lncRNAs is around 1.0, but there is a shoulder peak at the ratio of 0.8 and a small peak at a ratio of 0.5 (Figure 2F; K-S test p value = 1.3e-11). This suggests that a subset of *trans* lncRNAs is more inversely regulated than mRNAs in trisomy 2L males.

In addition to autosomal aneuploidy, we also analyzed the expression of lncRNAs and mRNAs in sex chromosome aneuploidy. For X-linked *cis* mRNAs, the distribution curve is around 1.0, indicating that the expression of most mRNAs is unchanged in XXX; AA metafemales (Figure 2G). The ratio distribution of *cis* lncRNAs is significantly different from that of mRNAs (K-S test p value = 2.5e-5), showing a multipeak shape, with the highest peak at 0.7–0.8, which may represent slightly excessive dosage compensation compared to the normal female level. There is a second peak at the ratio of 0.3, which may represent stronger inverse regulation, and a small peak at 1.1, which represents partial incomplete compensation (Figure 2G). The ratio distributions of *trans* lncRNAs and mRNAs are both biased to the left of 1.0 (Figure 2H), suggesting decreased expression levels due to the inverse dosage effect of the triple X genotype. However, lncRNAs are more left-shifted than mRNA (K-S test p value = 7.6e-13); that is, the expression level of lncRNAs is further down-regulated.

Compared with trisomy 2L males, in trisomy 2L females and metafemales, *cis* genes appear to be more likely to show dosage compensation, while *trans* genes tend to exist inverse dosage effects. Therefore, *cis* and *trans* genes are generally regulated in the same direction, which indicates that the *trans* genes affected by an inverse dosage effect and the *cis* compensated genes may be affected by related mechanisms. In addition, the inverse dosage effect seems to be stronger in females than in males, reflecting a sexual dimorphism of genomic imbalance. Similar to some mRNAs, lncRNAs are dosage-affected in aneuploid *Drosophila*, and there is dosage compensation of *cis* lncRNAs and inverse dosage effects of *trans* lncRNAs as described previously.[12,13,64] Whereas the responses of lncRNAs and mRNAs to the addition of an extra chromosome arm (whether autosomal or sex chromosome) are different, in that lncRNAs have a greater magnitude of expression modulation (Figures 2B, 2C, and 2E–2H). This result is also consistent with the comparisons of the mean CPM values of lncRNAs and mRNAs among samples (Figure 1F). Also, the sex chromosomes and autosomes have different characteristics in aneuploidy. In trisomy 2L *Drosophila*, only lncRNAs expressed from the X chromosome will produce ratio distributions that are not significantly different from mRNAs (Figures 2A and 2D).

Furthermore, we examined whether lncRNAs have similar responses in other species. In the five trisomies of *Arabidopsis* (Figure S2), the peaks of the ratio distributions of *cis* mRNAs are mostly between 1.0 and 1.5, which represents partial compensation to different degrees. The number of *cis* lncRNAs is small, showing a scattered distribution (Figures S2A, S2C, S2E, S2G, and S2I). The ratio distributions of mRNAs and lncRNAs located on the varied chromosomes are significantly shifted to the left of 1.0 in all aneuploidies, in which lncRNAs have main peaks or secondary peaks with smaller abscissae than mRNAs (Figures S2B, S2D, S2F, S2H, and S2J). As for the ratio distributions of four trisomies of mouse embryonic stem cells, *cis* mRNAs have a shoulder peak at the position below 1.5 (Figures S3A, and S3D), or have a main peak at the left side of 1.5 (Figures S3G, and S3J). The distributions of *cis* lncRNAs are more left shifted than that of mRNAs (K-S test p values <0.05), which is close to dosage compensation. The mRNAs expressed from the X chromosome are affected by autosomal aneuploidy, and their ratio distribution curves, centered at 1.0, form a shoulder peak on the left side; X-linked lncRNAs, on the other hand, produce a sharp peak at about

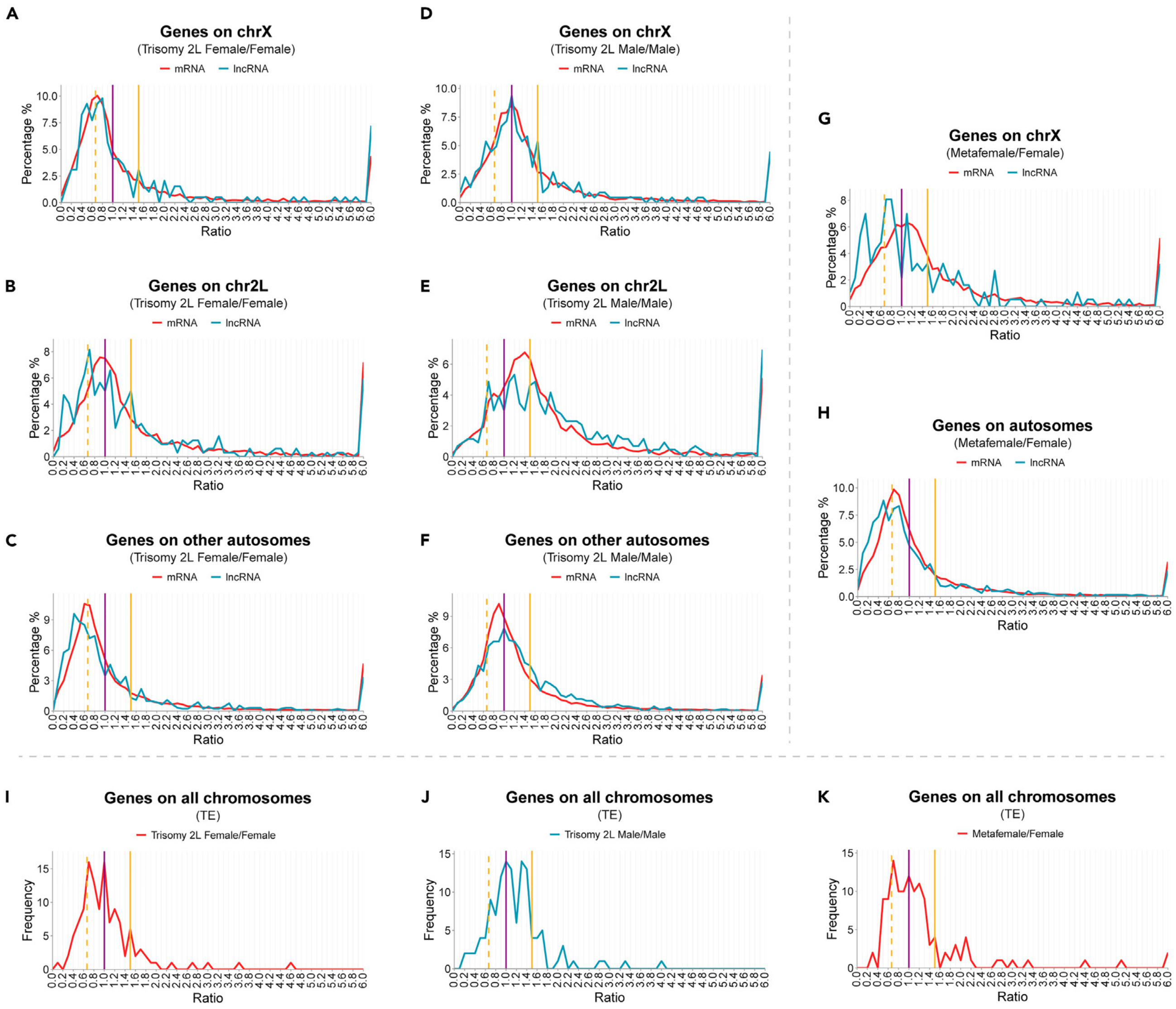

Figure 2. Distributions of the expression ratios of lncRNA and TE family in aneuploid *Drosophila*

(A–C) Ratio distributions of the expression levels of lncRNA and mRNA located on chromosome X (A), 2L (B), and other autosomes (C) in trisomy 2L females compared with wild-type females.

(D–F) Ratio distributions of the expression levels of lncRNA and mRNA located on chromosome X (D), 2L (E), and other autosomes (F) in trisomy 2L males compared with wild-type males.

(G and H) Ratio distributions of the expression levels of lncRNA and mRNA located on chromosome X (G) and autosomes (H) in metafemales compared with wild-type females.

(I–K) Ratio distributions of the expression levels of TE families in trisomy 2L females compared with wild-type females (I), trisomy 2L males compared with wild-type males (J), and metafemales compared with wild-type females (K).

The vertical purple solid line represents the ratio of 1.00, the vertical yellow solid line represents the ratio of 1.50, and the vertical yellow dashed line shows the ratio of 0.67. The ratio distributions were generated as described in STAR methods, and the percentages of frequencies were plotted in bins of 0.1.

0.7, which represents the consequence of an inverse dosage effect (Figures S3B, S3E, S3H, and S3K). For the genes on unvaried autosomes, the ratio distributions show that the expression levels of most mRNAs in mouse trisomy cells have not changed, but lncRNAs have relatively more left-leaning curves (Figures S3C, S3F, S3I, and S3L), indicating that lncRNAs might be more sensitive to genomic imbalance. In another dataset of induced-pluripotent stem-derived vascular endothelial cells (iPSC-derived ECs) of human trisomy 21 (Figure S4), it is found that, compared with wild type, or isogenic corrected disomy 21 cells, or both of them together, the ratio distribution curves of mRNAs and lncRNAs on chromosome 21 have main peaks at 1.0, which means that most *cis* genes are completely compensated. It is even found that lncRNAs have a peak around 0.4 when compared with isogenic corrected disomy 21 cells. The main peaks of mRNAs on the X chromosome and other autosomes are all located at 1.0, and there is a shoulder peak on the left (Figures S4B, S4C, S4E, S4F, S4H, and S4I). The distribution curves of

trans lncRNAs have peaks whose abscissae are smaller than that of mRNAs (Figures S4B, S4E, and S4H), or peaks with an overall left deviation (Figures S4C, S4F, S4I; K-S test p values <0.05). Generally speaking, the datasets of three different species we have analyzed all show that lncRNA and mRNA can be affected by genome imbalance, and lncRNA seems to be more strongly affected by inverse dosage effect than mRNA in aneuploidy. Therefore, the inverse dosage effect and the highly dosage sensitivity of lncRNAs in aneuploidy are generalizable across taxa.

Due to the high conservation of DNA sequences among insertions of the same TE family, we combined all insertions by TE family and calculated their expression. The ratio distributions of TE families no longer distinguish *cis* and *trans* chromosomes. In trisomy 2L females, the two main peaks of the ratio distribution are observed at ratios of 1.0 and 0.7, representing the TEs with unchanged expression and the TEs that are subject to an inverse dosage effect (Figure 2I). The ratio distribution curve of trisomy 2L males is different from that of females (K-S test p value = 0.0103), with two main peaks located at ratios of 1.0 and 1.3 (Figure 2J), indicating complete and incomplete dosage compensation, respectively. Meanwhile, there is a small peak at 0.7, which indicates that some TEs are regulated by an inverse dosage effect. In metafemales, the main peak of the distribution is located at 0.7 (Figure 2K), indicating that the expression of most TEs is inversely regulated. The second highest peak is at 1.0, representing no change in expression levels or dosage compensation. Therefore, TEs are also dosage-affected in aneuploidy and regulated by inverse dosage effects to a certain degree.

Differential expression analysis of lncRNAs in aneuploid *Drosophila*

Next, we performed differential expression analysis for different types of transcripts in aneuploid *Drosophila* to provide a statistical description of the lncRNAs and mRNAs affected by aneuploidy. We identified 6,195, 3,121, and 5,300 differentially expressed mRNA (DE-mRNA) transcripts in trisomy 2L females, trisomy 2L males, and metafemales, respectively (Figure S5A). Trisomy 2L females and metafemales share more DE-mRNAs, while trisomy 2L females and trisomy 2L males have slightly fewer common DE-mRNAs (2,774 compared with 1,507). In terms of DE-mRNAs, aneuploidies of the same sex and different chromosome arms are more similar than aneuploidies of different sexes and the same chromosome arm. Furthermore, the number of DE-mRNAs shared by any two aneuploidies is significant (Fisher's exact test p value <2.2e-16), suggesting that multiple aneuploidies can affect the same genes. There are 829 common DE-mRNAs in all three aneuploidies (Figure S5A), and their clustering shows obvious regularity (Figure S5B). The expression levels of more than half of the common DE-mRNAs in aneuploidy are higher than those in diploid, but there is still a large number of DE-mRNAs down-regulated (Figure S5B). Functional enrichment analysis of these common DE-mRNAs reveals that they are mainly involved in multiple biological metabolic processes and have various enzyme activities (Figure S5C). Their intracellular locations are mainly in the proteasome, peptidase complex, and CMG complex. In addition, the pathway analysis of these mRNAs also focuses on metabolism and the proteasome (Figure S5D). These results are consistent with the fact that aneuploidy is generally characterized by altered metabolism, reduced viability, abnormal protein production, and increased sensitivity to conditions interfering with protein synthesis, folding, and degradation.[3,4,70]

For lncRNAs, we found 266, 108, and 239 differentially expressed transcripts in trisomy 2L females, trisomy 2L males, and metafemales, respectively (Figure 3A). The rules found with mRNAs seem to be equally applicable to lncRNAs. The number of differentially expressed lncRNAs (DE-lncRNAs) shared by trisomy 2L females and metafemales is greater than the number of DE-lncRNAs shared by trisomy 2L females and trisomy 2L males (81 compared with 31). Moreover, the intersection of any two groups of DE-lncRNAs is significant (Fisher's exact test p value <0.05), indicating that the modulation of lncRNAs in aneuploidy is also gene-specific. Seventeen lncRNAs are differentially expressed in the three aneuploidies (Figure 3B). To gain a better picture, we analyzed a total of 110 lncRNAs that are significantly differentially expressed in at least two comparisons (Figure S6).

Volcano plots were drawn for all DE-lncRNAs, and the numbers of significantly up-regulated and down-regulated transcripts were counted (Figures 3C–3E). There are more up-regulated lncRNAs in trisomy 2L females and trisomy 2L males, but the number of significantly down-regulated lncRNAs is greater than the number of up-regulated lncRNAs in metafemales (Figures 3C–3E). Significantly down-regulated lncRNAs are found in all three trisomies, indicating that there is a subset of lncRNAs that are subjected to statistically significant inverse regulation, especially in sex chromosome aneuploidy. We labeled the DE-lncRNAs which have known GO functions in the plots (Figures 3C–3E), and listed their functions in the table (Figure 3F). The majority of them are related to spermatogenesis (for example, *lncRNA:CR43306*), and several others are related to growth and development (*lncRNA:CR31781*), gene expression regulation (*lncRNA:let7C*), and dosage compensation (*lncRNA:roX1* and *lncRNA:roX2*) (Figure 3F). In addition, the expression levels of *lncRNA:CR43306* and *lncRNA:CR31781* are significantly up-regulated in two aneuploidies, while the expression levels of *lncRNA:Hsromega*, which is related to nuclear omega speck organization and the regulation of protein metabolism, are significantly down-regulated in two aneuploidies (Figures 3C–3E).

Potential interactors of lncRNAs affected by aneuploidy

The function of most DE-lncRNAs we identified is unknown. Since lncRNAs may exert their functions by interacting with other factors in *cis* or *trans*,[32,65] we predicted their functions by looking for interactors co-expressed or co-located with DE-lncRNAs. The DE-lncRNA and DE-mRNA pairs whose absolute value of Pearson correlation coefficient >0.95 and p value <0.05 were considered as co-expressed. The lncRNA-mRNA pairs shared by at least two aneuploidies were selected to form a co-expression network, which contains 41 lncRNAs, 294 mRNAs, and a total of 568 edges (Figures 4A–4C). It can be observed that there are two large subnetworks in the whole co-expression network, which we call cluster 1 (Figure 4A) and cluster 2 (Figure 4B), in addition to some scattered small clusters (Figure 4C). Protein-protein interaction (PPI) analysis was performed for all DE-mRNAs in the network, and the connectivity degree of each mRNA was determined. Therefore, mRNAs with a higher degree, i.e., the redder nodes in Figure 4 (for example, *Ubi-p63E* encoding a polyubiquitin precursor, *Su(var)205* as a structural component of

CellPress
OPEN ACCESS

A

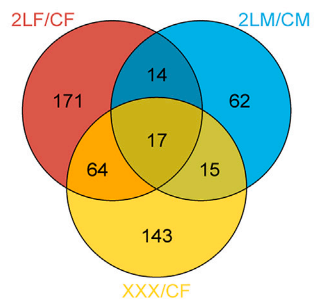

F

lncRNA	function
lncRNA:CR31781	lateral inhibition; expressed in embryonic/larval somatic muscle cell
lncRNA:CR43855	defense response to insect
lncRNA:CR44811	regulation of gene expression; negative regulation of imaginal disc-derived wing vein specification
lncRNA:CR43306	spermatogenesis
lncRNA:CR44420	spermatogenesis
lncRNA:CR45670	spermatogenesis
lncRNA:TS27	spermatogenesis
lncRNA:Hsromega	nuclear omega speck organization; positive regulation of cellular protein metabolic process; positive regulation of JNK cascade; regulation of proteolysis; positive regulation of apoptotic process
lncRNA:let7C	negative regulation of gene expression; mushroom body development; cellular response to ecdysone; expressed in adult brain and synaptic neuropil block
lncRNA:roX1	dosage compensation by hyperactivation of X chromosome; spreading of the MSL complex in cis
lncRNA:roX2	dosage compensation by hyperactivation of X chromosome; spreading of the MSL complex in cis

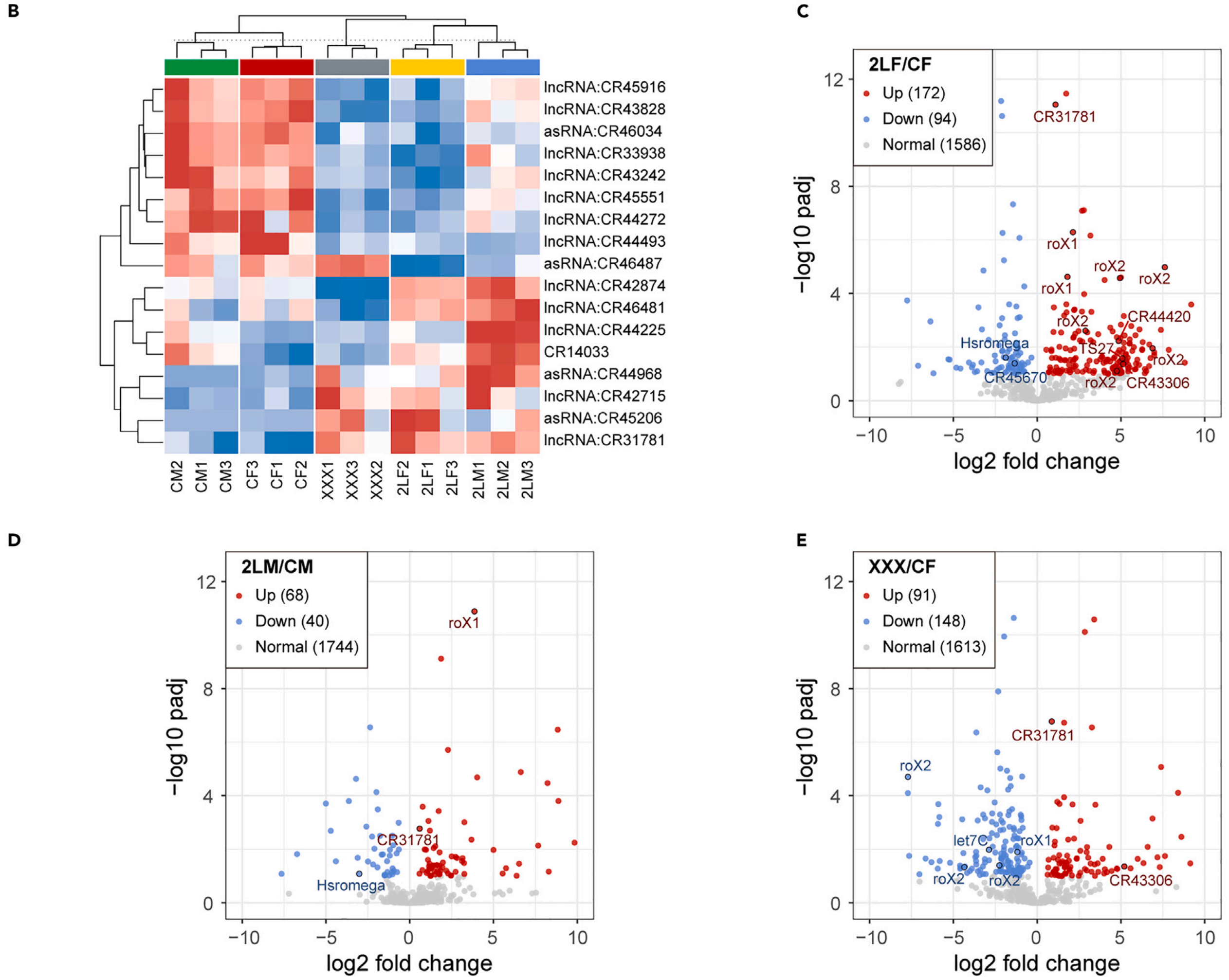

Figure 3. Differential expression analysis of lncRNA in aneuploid *Drosophila*

(A) Venn diagram of the number of differentially expressed lncRNA (DE-lncRNA) in trisomy 2L female, trisomy 2L male, and metafemale.

(B) Clustering heatmap of lncRNAs differentially expressed in all three aneuploidies.

(C–E) Volcano plots of DE-lncRNA in trisomy 2L female (C), trisomy 2L male (D), and metafemale (E). The positions of DE-lncRNAs with known functions are marked in the plots. The texts in the upper left corner are the statistics of the number of up- and down-regulated lncRNAs.

(F) List of lncRNAs with known functions that are differentially expressed in at least one aneuploidy.

CF, wild-type female control; CM, wild-type male control; 2LF, trisomy 2L female; 2LM, trisomy 2L male; XXX, metafemale.

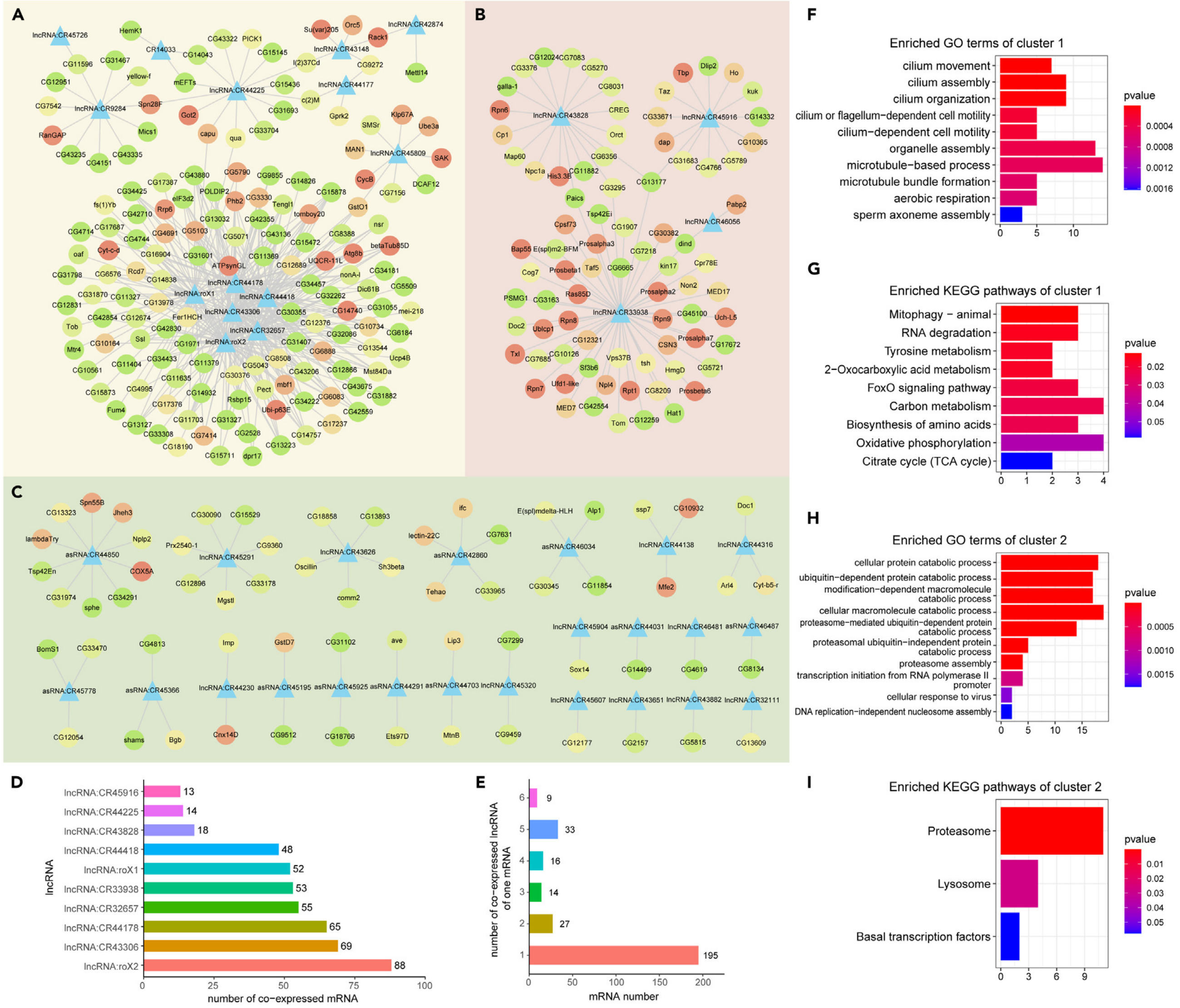

Figure 4. Co-expressed differentially expressed lncRNAs and mRNAs

(A–C) Network of co-expressed lncRNAs and mRNAs. The triangular nodes represent lncRNAs, and the round nodes represent mRNAs. The color of the round nodes indicates the degree of this mRNA in the protein-protein interaction (PPI) network composed of DE-mRNAs, with red indicating high connectivity and green indicating low connectivity. The lncRNA and mRNA connected by edges are co-expressed (the absolute value of Pearson correlation coefficient >0.95 and p value <0.05). The entire co-expression network can be divided into two larger clusters (A and B), and a number of smaller, dispersed clusters (C).

(D) The top 10 lncRNAs with the largest number of co-expressed mRNAs.

(E) The number of co-expressed lncRNA owned by per mRNA.

(F) GO functional enrichment analysis of mRNAs involved in the first large cluster of the co-expression network.

(G) KEGG pathway enrichment analysis of mRNAs involved in the first large cluster of the co-expression network.

(H) GO functional enrichment analysis of mRNAs in the second large cluster of the co-expression network.

(I) KEGG pathway enrichment analysis of mRNAs in the second large cluster of the co-expression network.

heterochromatin, *Ras85D* as an essential component involved in the signal pathway regulating growth and development, etc.), may play a more critical role in the dysregulation of expression in aneuploidy.

Further analysis of the lncRNA-mRNA co-expression network shows that some lncRNAs have numerous co-expressed mRNAs; for example, *lncRNA:roX2*, which plays a role in the regulation of X chromosome in *Drosophila* males, has 88 co-expressed mRNAs (Figure 4D). Many potential interactors may indicate the functions of these lncRNAs. On the contrary, most mRNAs have only one co-expressed lncRNA (Figure 4E). Next, functional enrichment analysis and pathway analysis were performed on the two large co-expression subnetworks (Figures 4F–4I). The results showed that the functions of cluster 1 are mainly related to microtubule-based processes, such as cilium

CellPress
OPEN ACCESS

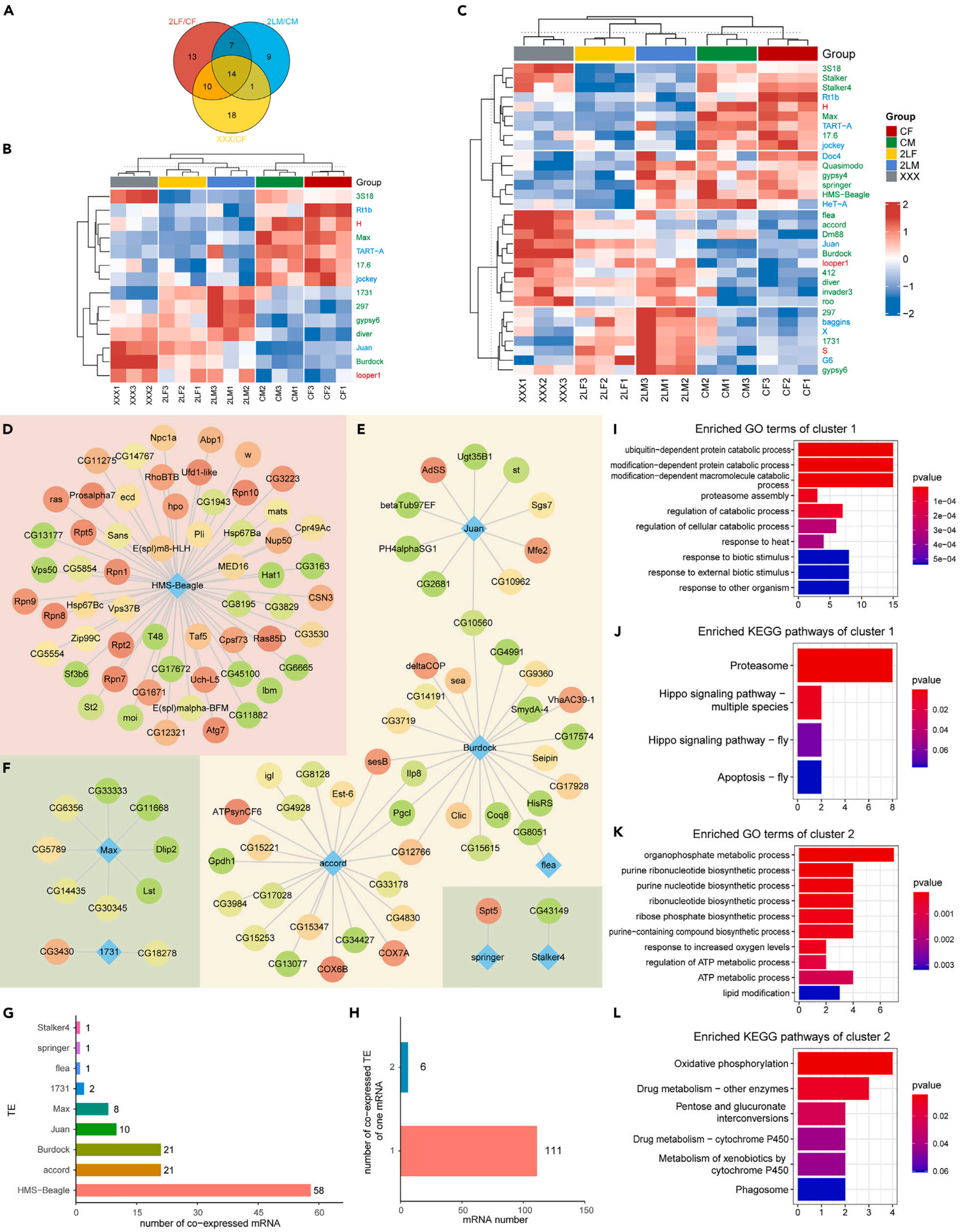

Figure 5. Co-expressed differentially expressed TE families and mRNAs

(A) Venn diagram of the number of differentially expressed TE families (DE-TEs) in trisomy 2L female, trisomy 2L male, and metafemale.

(B) Clustering heatmap of TE families differentially expressed in all three aneuploidies.

(C) Clustering heatmap of TE families differentially expressed in at least two groups of comparisons. The text colors of the TE families represented their classification, with green indicating LTR retrotransposons, blue indicating LINE-like TEs, and red indicating TIR transposons. CF, wild-type female control; CM, wild-type male control; 2LF, trisomy 2L female; 2LM, trisomy 2L male; XXX, metafemale.

(D–F) Network of co-expressed TE families and mRNAs. The diamond nodes represent TE families, and the round nodes represent mRNAs. The color of the round nodes indicates the degree of this mRNA in the PPI network composed of DE-mRNAs, with red indicating high connectivity and green indicating low connectivity. The TE family and mRNA connected by edges are co-expressed (the absolute value of Pearson correlation coefficient >0.95 and p value <0.05). The entire co-expression network can be divided into two larger clusters (D and E), and a number of smaller, dispersed clusters (F).

(G) TE families sorted by the number of their co-expressed mRNAs.

(H) The number of co-expressed TE families owned by one mRNA.

(I) GO functional enrichment analysis of mRNAs involved in the first large cluster of the co-expression network.

(J) KEGG pathway enrichment analysis of mRNAs involved in the first large cluster of the co-expression network.

(K) GO functional enrichment analysis of mRNAs in the second large cluster of the co-expression network.

(L) KEGG pathway enrichment analysis of mRNAs in the second large cluster of the co-expression network.

movement, cilium organization, and sperm axoneme assembly (Figure 4F); some pathways are enriched by genes in cluster 1, including mitophagy, RNA degradation, amino acid metabolism, aerobic respiration, and so on (Figure 4G). Cluster 2 is mainly enriched in proteasome mediated ubiquitin-independent protein catabolic process, proteasome assembly, and transcription initiation (Figure 4H). Its enriched pathways are proteasome, lysosome, and basal transcription factors (Figure 4I). Using interacting mRNAs to predict the functions of individual lncRNAs (Figure S7A), most of the lncRNAs in cluster 1 are assumed to function in cilium assembly and movement, sperm axoneme assembly, oxidative phosphorylation, biosynthesis of amino acids, mitophagy, and peroxisomes. The lncRNA in cluster 2, represented by *lncRNA:CR33938*, is speculated to have ubiquitin-dependent protein catabolic process, proteasome assembly, cell fate specification, and other important functions affecting various developmental processes. For the entire lncRNA-mRNA co-expression network, the enriched functions or pathways with the names of genes that contributed to the enrichment were displayed using cnetplots (Figures S7B and S7C).

Furthermore, we analyzed DE-mRNAs close to DE-lncRNAs in genome location (within 10 kb), and constructed a co-located lncRNA-mRNA network. This network consists of 89 co-located lncRNA-mRNA pairs, but they are so dispersed that no major clusters are formed (not shown). For each lncRNA, most have one or two co-located mRNAs; while for each mRNA, only one lncRNA co-located with it (Figures S8A and S8B). Enrichment analysis revealed that the functions of genes in co-localization network are mainly enriched in somatic muscle development, myoblast fusion, organic hydroxy compound metabolic process, and negative regulation of the Notch signaling pathway (Figure S8C). There were also enrichments of multiple metabolic pathways, such as fatty acid biosynthesis, amino sugar and nucleotide sugar metabolism, inositol phosphate metabolism, and carbon metabolism (Figure S8D).

Differentially expressed TE families and their interactors

We additionally performed differential expression analysis for TE families. 44, 31, and 43 differentially expressed TE families (DE-TEs) are found in trisomy 2L females, trisomy 2L males, and metafemales, respectively (Figure 5A). Similar to mRNA and lncRNA, the number of DE-TEs shared by trisomy 2L females and metafemales was greater than the number of DE-TEs shared by trisomy 2L females and trisomy 2L males (24 compared with 21), and the species of differentially expressed families was significantly correlated (Fisher's exact test p values <0.05). Fourteen DE-TEs were present in all three aneuploidies (Figure 5B), while 32 TE families were differentially expressed in at least two groups (Figure 5C). Furthermore, most of these DE-TEs are LTR or LINE-like retrotransposons (Figures 5B and 5C).

Subsequently, we performed co-expression analysis of these TE families with DE-mRNAs (Figures 5D–5F). The TE-mRNA co-expression network includes 9 TE families, 117 mRNAs, and 123 pairs of interactions. This network can also be further divided into two smaller subnetworks, cluster 1 and cluster 2 (Figures 5D and 5E), and some scattered clusters (Figure 5F). The DE-TE with the most potential interactors is an LTR transposon, *HMS-Beagle*, with 58 co-expressed mRNAs (Figure 5G). On the contrary, there are only one or two co-expressed TE families per mRNA, and the majority are one (Figure 5H). Enrichment analysis of cluster 1 with the core of *HMS-Beagle* showed that its functions are mainly concentrated in ubiquitin-dependent protein catabolic process, proteasome assembly, response to heat, and response to biotic stimulus (Figure 5I). Pathway analysis found that the proteasome, apoptosis, and Hippo signaling pathways are enriched (Figure 5J). The genes in cluster 2 are enriched with lipid modification, ATP metabolic process, purine nucleotide biosynthetic process, and other metabolic processes (Figure 5K). For cellular pathways, they are enriched in oxidative phosphorylation, drug metabolism, phagosome, etc. (Figure 5L). We also predicted the functions of individual TE families based on their co-expressed mRNAs (Figure S9A), and identified specific mRNAs that are responsible for the enriched functions and pathways (Figures S9B and S9C).

Expression patterns of dosage-affected lncRNAs and their interactors in aneuploid *Drosophila* embryos

To examine the developmental expression patterns of the dosage-affected noncoding genes identified previously, we designed probes (Figure S10) for several lncRNAs and their potential interactors which was used for tyramide signal amplification (TSA)-based fluorescence *in situ* hybridization (FISH) of *Drosophila* embryos. With high resolution, sensitivity, and consistency, TSA-FISH can provide information about the temporal and spatial patterns of RNA expression and allow us to predict the function of RNA based on their subcellular and subembryonic

Table 1. Information of candidate genes

Group	Genes	Type	Function
Co-expressed cluster 2	lncRNA:CR33938	lncRNA	lncRNA:CR33938 is highly expressed in the distal cells of the third instar larval leg disc and has potential roles in regulating distal leg development.
	CG3295	mRNA	Predicted to enable ubiquitin protein ligase and transferase activity. Involved in positive regulation of proteasomal ubiquitin-dependent protein catabolic process. Part of GID complex.
	HmgD	mRNA	HmgD encodes a highly abundant chromosomal protein involved in DNA bending and chromatin organization.
	tsh	mRNA	tsh is a homeotic protein that determine segment identity throughout the entire trunk during embryogenesis. It has multiple development, transcription, and signaling functions.
Co-expressed cluster 2	lncRNA:CR45916	lncRNA	Predicted to be involved in miRNA-mediated gene silencing. Predicted to be part of RISC complex.
	dap	mRNA	dap encodes a Cyclin-dependent kinase inhibitor in the CIP/KIP family. It binds to CycE-Cdk2 complexes and thereby inhibits their protein kinase activity.
Co-expressed small cluster	asRNA:CR42860	lncRNA	–
	Tehao	mRNA	It is involved in the Toll signaling pathway and innate immune response.
	ifc	mRNA	Sphingolipid-delta-4-desaturase activity. Required to initiate spermatid differentiation among other signals. Required for central spindle assembly and cytokinesis during male meiosis.
Co-expressed cluster 1	lncRNA:CR43148	lncRNA	–
	Su(var)205	mRNA	Structural component of heterochromatin, involved in gene repression and the modification of position-effect variegation. Recognizes and binds histone H3 tails methylated at Lys-9, leading to epigenetic repression.
Co-expressed cluster 1	lncRNA:CR44178	lncRNA	–
	nonA-l	mRNA	Predicted to enable mRNA binding activity. Predicted to be involved in regulation of transcription, DNA-templated. Predicted to be active in nucleus. Is expressed in adult head.
	lncRNA:CR44418	lncRNA	–
	Atg8b	mRNA	Atg8b encodes a ubiquitin-like protein and is a member of the autophagy-related gene family. Atg8b has an abundant testis-specific expression and has roles in the maintenance of sperm motility.
Transposable element	HMS-Beagle	TE	–

GID complex, glucose-induced degradation-deficient complex; RISC, RNA-induced silencing complex; CIP/KIP family, CDK-interacting protein/kinase inhibitory protein family; CycE-Cdk2 complex, Cyclin E/Cyclin-Dependent Kinase 2 complex; TE, transposable element.

localization.[8] A total of sixteen candidate genes that may perform different functions were selected for detection, including co-expressed lncRNA-mRNA pairs, as well as a TE family that has the highest connectivity in the co-expression network (Table 1). Full results are shown in Data S1, including subembryonic and subcellular localization of probes and corresponding descriptions; the relative quantification of signal intensities at three developmental stages is also listed.

Stage 1–5 and Stage 6–11 include many important early developmental markers, such as the maternal-zygotic transition and the germband elongation. It was found that the zygotically expressed genes in early embryogenesis are enriched in transcription regulation, cell fate determination, tissue and organ development, and morphogenesis.[8] The candidate gene *tsh* is considered to be able to regulate the development of eye, leg, midgut, head, and prothoracic segment, and participate in transcription regulation and Wg signaling pathway. It is also one of the genes with interesting expression patterns in our FISH experiment. In the two early periods, the subembryonic localizations of *tsh* show segmented patterns, which are distributed in the midposterior and posterior part of the embryos respectively; and the localization of *tsh* in wild type and trisomy 2L are similar (Figures 6A, 6A′, 6B, 6B′, and Data S1). Therefore, *tsh* may be a vital gene to maintain early embryogenesis. For lncRNAs, although the functions of the majority are unknown, specific expression patterns can also be detected. For example,

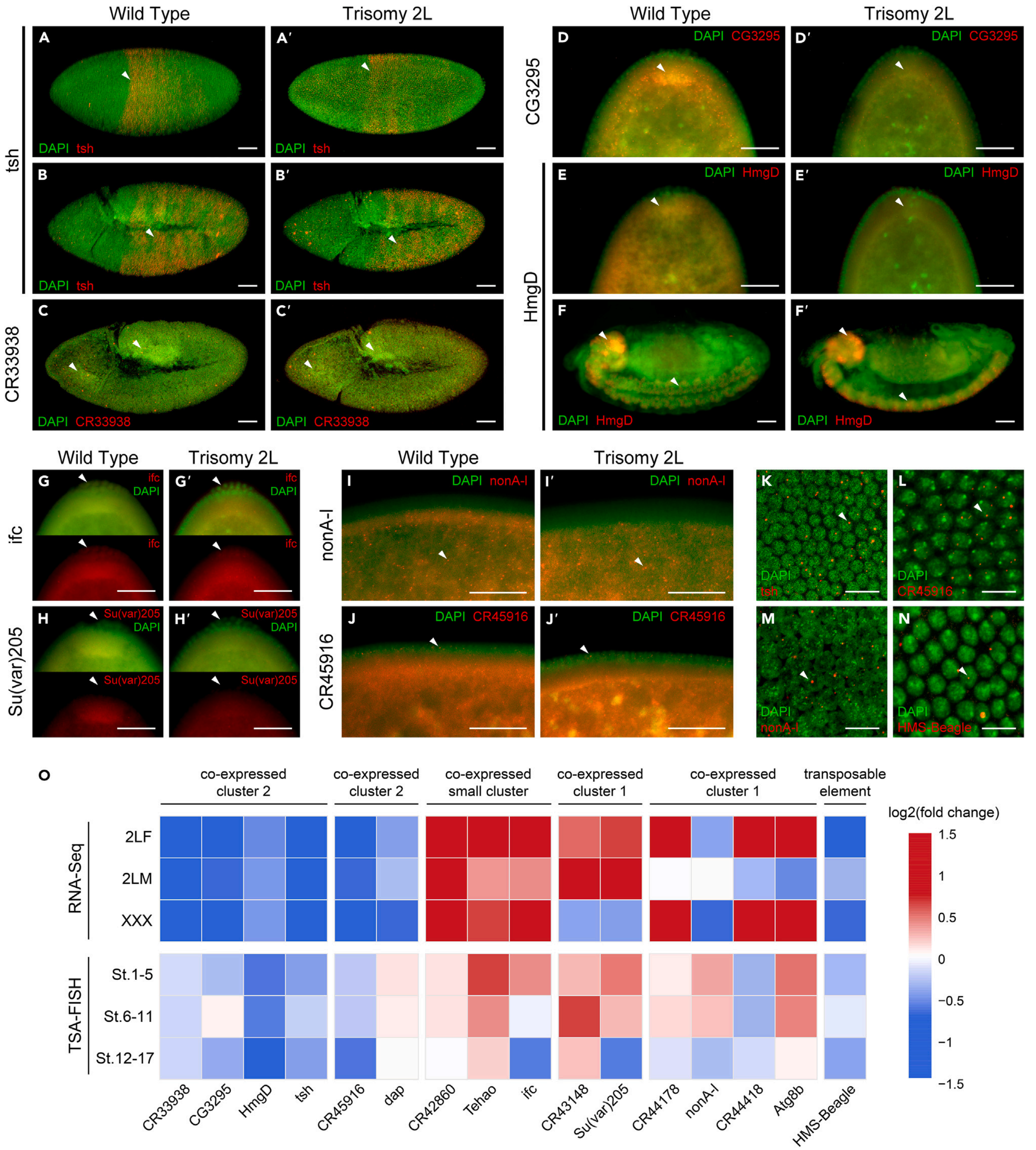

Figure 6. Expression patterns of candidate genes in embryonic development of *Drosophila*

(A–J) Subembryonic distribution of probes in wild-type *Drosophila*. (A′-J′) Subembryonic distribution of probes in Trisomy 2L *Drosophila*. The name of the gene is shown in the left of the picture, and the genotype is shown above. Red, probe; green, DAPI. Arrowheads indicate regions of enriched or differential probe signal. Scale bars, 50 μm.

(K–N) Subcellular localization of probe signals. Probe names are written in the lower left corner of the picture. Red, probe; green, DAPI. Arrowheads indicate the foci of probe signals. Scale bars, 10 μm.

(O) Expression changes of candidate genes in aneuploid *Drosophila* detected by RNA-seq and TSA-FISH. The colors of the heatmap represent log2 fold changes in trisomy compared with wild type. 2LF, trisomy 2L female; 2LM, trisomy 2L male; XXX, metafemale. FISH data represents the mean value of 10 embryos.

CellPress
OPEN ACCESS

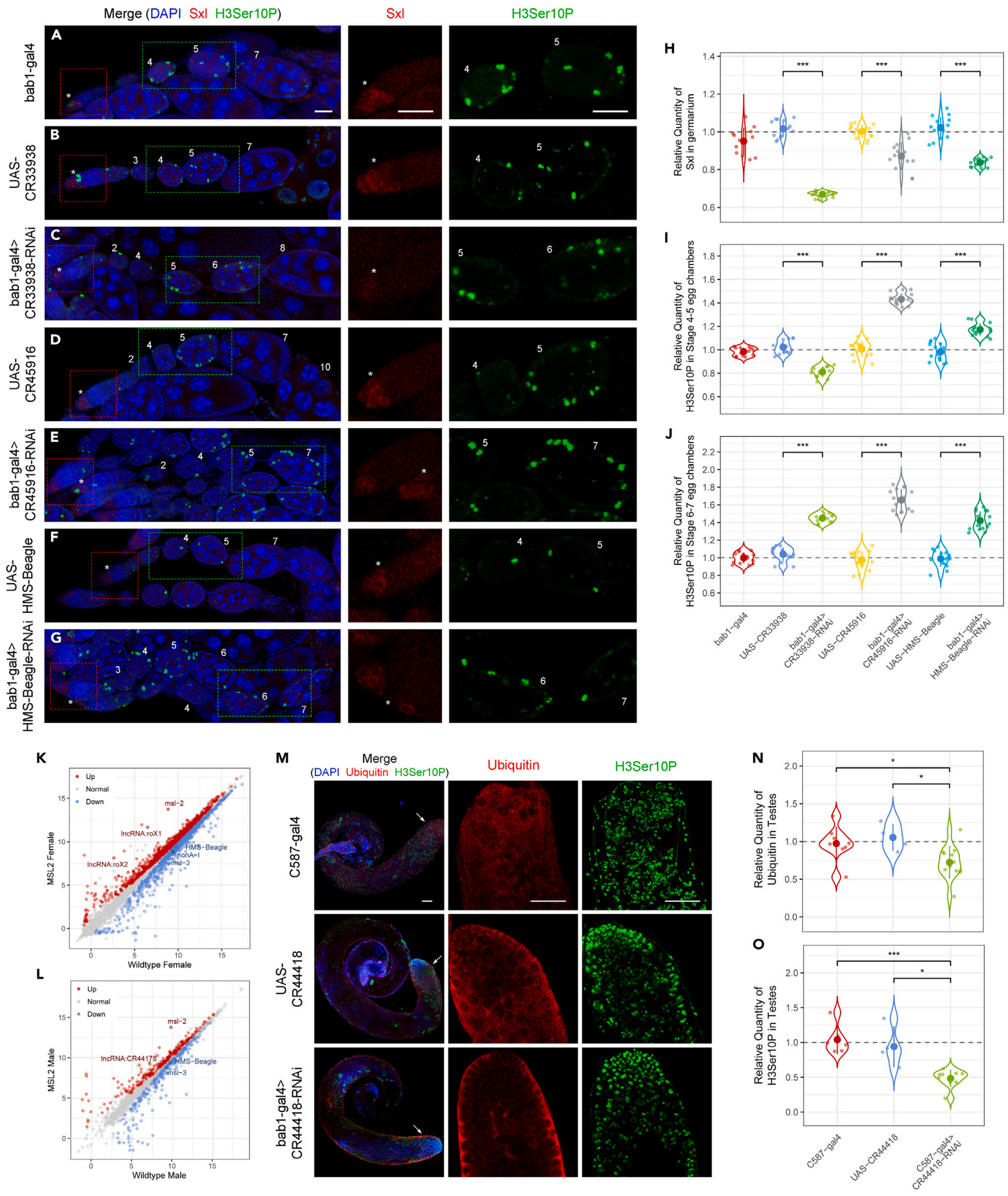

Figure 7. Immunofluorescence staining of ovaries and testes from lncRNAs and TE knockdown *Drosophila*

(A–G) Immunofluorescence staining of *Drosophila* ovaries. Each multi-color fluorescent photograph focuses on one ovariole whose egg chambers is marked with the development stages, and an asterisk indicates the germarium. The genotypes are annotated on the left side of each row. Red, SXL; green, H3Ser10P; blue,

Figure 7. *Continued*

DAPI. The red dashed squares indicate germarium and are enlarged in the middle panels. The green dashed rectangles indicate egg chambers and are enlarged in the right panels. Scale bars, 25 μm.

(H) Relative quantification of SXL at the top of germarium based on fluorescence intensity. Sample size ≥12.

(I and J) Relative quantification of H3Ser10P in stage 4–5 (I) and stage 6–7 (J) egg chambers based on fluorescence intensity. Sample size ≥12.

(K and L) Scatterplots of gene expression after overexpression of MSL2 in female (K) and male (L) *Drosophila*. The axis indicate the average expression of a gene, in the form of regularized-logarithm transformation. Red and blue dots represent significantly up-regulated and down-regulated genes, respectively (padj < 0.1). DEGs in the candidate genes mentioned above and MSL complex are marked with their symbols.

(M) Immunofluorescence staining of *Drosophila* testes. The apex of the testes, indicated by the arrows, is shown in the middle and right panels. The genotypes are annotated on the left. Red, Ubiquitin; green, H3Ser10P; blue, DAPI. Scale bars, 50 μm.

(N and O) Relative quantification of Ubiquitin and H3Ser10P in *Drosophila* testes based on fluorescence intensity. Sample size ≥4. Student's *t* test *p < 0.05, **p < 0.01, ***p < 0.001.

lncRNA:CR33938, co-expressed with *tsh*, is mainly enriched in the head and amnioproctodeal invagination in St. 6–11, though its probe signals show a ubiquitous distribution in St.1-5 (Figures 6C, 6C′, and Data S1).

Two other genes, *CG3295* and *HmgD*, in the same co-expression cluster have different localizations in trisomy 2L and wild-type embryos. *CG3295*, which is involved in regulation of proteasomal ubiquitin-dependent protein catabolic process, loses its localization at posterior yolk plasm in the trisomy 2L blastoderm (Figures 6D, 6D′, and Data S1) while *HmgD*, which is related to transcription regulation and chromatin organization, not only loses its posterior yolk plasm localization in trisomy 2L but also shows an apical enrichment that is not found in wild-type embryos (Figures 6E, 6E′, and Data S1). The distribution changes of *CG3295* and *HmgD* may lead to subsequent disturbance of ubiquitination and chromatin organization, which may be associated with the abnormal development of trisomy 2L. In addition, signals of *HmgD* are strongly enriched in the brain and ventral nerve cords of *Drosophila* embryos during the third period (Stage 12–17) (Figures 6F and 6F′, and Data S1), suggesting the possible nervous system function of *HmgD*.

The expression patterns of genes in the other co-expressed clusters also reflect their biological functions. For example, the probe signals of *ifc* related to spermatogenesis are enriched in posterior yolk plasm and pole cell in Stage 1–5, indicating a reproductive related function (Figures 6G, 6G′, and Data S1). *Su(var)205*, which is associated with heterochromatin assembly, shows a pole cell exclusion pattern, and the posterior yolk plasm localization disappears (Figures 6H, 6H′, and Data S1). Both *nonA-l*, which is related to mRNA splicing, and *lncRNA:CR45916*, which is involved in miRNA-mediated gene silencing, have small foci distributed on the surface of blastoderms, and both have intranuclear subcellular localizations (Figures 6M, 6L, and Data S1). Notably, several probes are enriched in posterior yolk plasm in wild-type embryos in St. 1–5, but not in trisomy 2L (Data S1). These common localization differences show that trisomy 2L has an impact starting in early embryonic development. The characteristic events of *Drosophila* embryonic development in the third period (St. 12–17) are dorsal closure, head involution, and midgut closure. Most of the candidate probes can be detected in the brain, midgut, hindgut, proventriculus, amnioserosa, and the medial side of the embryos during St. 12–17 (Data S1). This may be due to the full activation of zygote genome and generally active transcription of genes in the late embryonic development, in preparation for entering the larval stage.

At the subcellular level, the distribution patterns of most probes (87.5%) are or at some stages are perinuclear (Data S1). *tsh*, *lncRNA:CR45916*, *nonA-l*, and transposable element *HMS-Beagle* have intranuclear localization patterns (Figure 6K–6N). Among the nine co-expressed pairs determined by RNA-seq results, five pairs of lncRNA and mRNA have the same subcellular localization patterns, validating the interaction relationship between these lncRNAs and mRNAs. In addition, there is no visible difference in subcellular distributions of all candidate genes between wild type and trisomy 2L embryos.

The expression levels of candidate genes in wild type and trisomy 2L *Drosophila* embryos were analyzed according to the relative fluorescence intensities of probes. Compared with the wild type, the expression levels of most candidate genes changed significantly in trisomy 2L regardless of whether the probe distributions changed or not (Data S1). The expression changes of 75% (12/16) of the candidate genes in most developmental stages are consistent with the results of RNA sequencing (Figure 6O and Data S1). Furthermore, according to the RNA-seq results, sixteen candidate genes contain nine groups of co-expression relationships, and five co-expression relationships exist in the FISH experiment, which confirms the association between these lncRNAs and mRNAs. The differences between sequencing results and FISH quantification may be due to the different development stages of detection.

Biological functions of candidate lncRNAs and TEs in *Drosophila* gonads

To further investigate the biological functions of candidate noncoding genes, we used the GAL4-UAS system to knock down several candidate lncRNAs and TE in *Drosophila* gonads. The ovary of female *Drosophila* consists of dozens of ovarioles containing sequentially developing egg chambers, with the germarium at its anterior. The Sex-Lethal (SXL) protein is required for germline stem cells (GSCs) to enter the differentiation pathway. In the absence of SXL, germ cells will be blocked in a state between GSC and cystoblast.[71] According to immunofluorescence staining, SXL protein was only enriched in the cytoplasm of a few cells at the top of the germarium (Figures 7A–7G). There was no obvious difference in its location in *lncRNA:CR33938*, *lncRNA:CR45916*, and the TE *HMS-Beagle* ovarian-specific knockdown lines compared with the control. However, the relative quantity of SXL in germarium of these RNAi *Drosophila* has been down-regulated, especially in *lncRNA:CR33938* RNAi strain (Figure 7H). Studies have reported that lack of SXL will lead to continued proliferation and germline tumors that inappropriately express a set of male specific markers.[72] In our experiment, lncRNA or TE knockdown lines showed reduced SXL levels, but no ovarian tumor phenotype was observed.

CellPress
OPEN ACCESS

The development of egg chambers is divided into 14 stages, which can be determined by the morphology and size of the egg chamber.[73] In addition to the germline cells, the egg chambers also include a layer of somatic follicle cells. Follicle cells undergo mitosis until stage 5, after which they undergo the mitosis-endocycle (M/E) transition and begin to differentiate. When the mitotic marker H3Ser10P was used to indicate the dividing cells, the positive signal in the normal ovary was mainly distributed in the germarium, and follicle cells of the egg chambers before stage 6 (that is, before the M/E switch) (Figures 7A, 7B, 7D, and 7F). However, in the three ovarian-specific RNAi strains, it was observed that the H3Ser10P signal of follicle cells persisted to stage 6 or even stage 7 (Figures 7C, 7E, and 7G). Therefore, knocking down *lncRNA:CR33938*, *lncRNA:CR45916*, or *HMS-Beagle* may interfere the M/E transition of follicle cells, and affect the development of *Drosophila* ovary. Relative quantification of the signal intensity of H3Ser10P revealed that in *lncRNA:CR33938* RNAi strain, the level of H3Ser10P was significantly decreased in stage 4–5 egg chambers, but increased in stage 6–7 egg chambers (Figure 7I and 7J). It is suggested that *lncRNA:CR33938* may play a role in female reproduction during oogenesis, and its deficiency will not only reduce the level of SXL in germarium but also delay the development of the egg chambers. For *lncRNA:CR45916* and *HMS-Beagle* RNAi, H3Ser10P was significantly increased in both stage 4–5 and stage 6–7 egg chambers (Figures 7I and 7J), which shows disruption of follicle cell proliferation and abnormal ovarian development.

Based on the results of RNA sequencing, both *lncRNA:CR33938* and *HMS-Beagle* have more than fifty co-expressed mRNAs, many of which have developmental regulatory functions (Figures 4B, 4D; 5D, and 5G). For example, *Prosalpha7*, *CSN3*, *Ras85D*, which are co-expressed with *lncRNA:CR33938*, and *Atg7*, *ecd*, *mats*, which are co-expressed with *HMS-Beagle*, have ovarian related functions such as oogenesis, ovarian follicle cell or germ cell development. In addition, *dap*, which is co-expressed with *lncRNA:CR45916* functions in oocyte fate determination and germline cell cycle switching. There is also a link between *HMS-Beagle* and the male-specific lethal (MSL) complex, as the expression of *HMS-Beagle* was significantly down-regulated in the transcriptome data of MSL2 overexpressed *Drosophila* (Figures 7K, and 7L). These results indicate that candidate lncRNAs and TE are important, which may affect sex determination and dosage compensation, or gonad development and reproductive ability of *Drosophila* by interacting with SXL, MSL2, and their co-expressed protein-coding genes.

Another candidate gene *lncRNA:CR44418* has 48 co-expressed mRNAs (Figures 4A and 4D), of which five (*Atg8b*, *CG42355*, *Mst84Da*, *nsr*, and *Rcd7*) have spermatogenesis related functions, and the co-expressed genes are enriched with GO term of Ubiquitous-dependent protein catabolic process (p value = 2.0e-12). Therefore, we examined the level of ubiquitin in the testes of *lncRNA:CR44418* gonad-specific knockdown *Drosophila*. It was found that ubiquitin signal was mainly concentrated in the apex, but it was also widely distributed in other parts of the testes (Figure 7M). Although the distribution of ubiquitin in the testes of *lncRNA:CR44418* RNAi *Drosophila* was unchanged, its relative level was significantly down-regulated (Figure 7N). Since ubiquitination is required for chromatin remodeling and spermatid terminal differentiation (i.e., individualization) of *Drosophila* sperm,[74,75] reduced expression of *lncRNA:CR44418* may lead to decreased ubiquitination in testes and impair spermatogenesis. Furthermore, we also observed a significant decrease of H3Ser10P signal in RNAi testes (Figure 7O), implying a reduction in cell division and a disturbance in the development of male gonads.

DISCUSSION

Dosage changes of single chromosomes or chromosome segments (aneuploidy) in organisms are usually more detrimental than the increase or decrease of a whole set of chromosomes (euploid variation) known as genetic imbalance.[15–17] This phenomenon has been found in different taxa such as *Drosophila*, maize, *Arabidopsis*, *Datura*, and human cells.[10,11,16] Some view that the expression of genes on the varied chromosomes will change proportionally, and this is the primary basis of the deleterious phenotype of aneuploidy.[4,76] However, others found partial genome changes can produce extensive regulation on the expression of both *cis* and *trans* genes.[8–13,77–80] A few studies have examined the expression changes of protein-coding genes in aneuploidy,[10–13,63,64,77] with a focus on several potential dosage-sensitive factors, mostly components of protein complexes.[16,64,77] In this study, we performed an analysis of the expression and characteristics of lncRNAs and TEs in aneuploid *Drosophila*, as well as their potential interactors. Their distribution patterns during embryonic development and biological functions in gonads were also investigated. It was demonstrated that lncRNAs and TEs are dosage affected and may be involved in gene expression modulation and development control of aneuploidy.

The patterns of expression in aneuploidy were first detected in maize, which are also applicable to other species.[16,81] Early studies of genomic imbalance revealed a phenomenon, in which the genes without copy-number changes in aneuploidy are inversely modulated, which is called the inverse dosage effect.[81,82] Later, with the rapid development of high-throughput sequencing technology, comprehensive studies were carried out on different dosage series of segmental aneuploidies.[11,77,83] It was found that the expression of *cis* genes generally ranged from compensation to dosage effect, while the expression of *trans* genes mainly ranged from unchanged to negatively correlated with chromosome changes.[10,11,77] *Trans* genes tend to show an inverse dosage effect when *cis* genes approach dosage compensation.[11,77] *Drosophila*, with a relatively simple chromosome complement, also has many in-depth studies on genomic imbalance.[16,18] In addition, these studies also revealed sexual dimorphism in aneuploid *Drosophila*, that is, differences between sex-biased and non-sex-biased genes, and the specificity of the response of sex chromosomes.[13,63] The fact that the inverse dosage effects act on the entire genome suggests that the dosage compensation of *cis* genes results from a simultaneous inverse modulation that counteracts the direct proportional dosage effect that might otherwise occur.[64,84,85]

Previous aneuploidy studies have seldom distinguished between protein-coding and noncoding genes, and most have paid little attention to whether different types of genes have different responses. Only a few studies examined the dosage-dependent expression of some atypical RNAs, for example, serine-4 transfer RNA and *copia* retrotransposons were found to show dosage compensation.[86,87] A recent study examined the dosage sensitivity of miRNAs in a series of aneuploid maize, and found that miRNAs are affected as well and could act as regulators in aneuploidy.[25,88] We performed an analysis of mRNAs, lncRNAs, and TEs in aneuploid *Drosophila* and revealed that the expression

of lncRNAs and TEs can also be affected by genomic imbalance. Similar to protein-coding genes, a subset of *cis* noncoding genes is dosage compensated, while *trans* noncoding genes mostly show down-regulation in trisomies. The down-regulation of lncRNAs is generally more significant than that of mRNAs, indicating that lncRNAs maybe more strongly affected. The greater sensitivity of lncRNAs was documented in different species. Furthermore, the responses of lncRNAs are distinct between males and females in aneuploid *Drosophila*, and the expression of females tends to be lower, which represents a sexual dimorphism. The specific responses of lncRNAs on the X chromosome were also demonstrated. For TEs, although the expression levels of each insertion were not analyzed by chromosomal location, the overall expression of TE families also showed that TEs are dosage affected, with their expression mainly inversely regulated, and are sexually dimorphic.

It is reasonable that lncRNAs and TEs have similar dosage sensitivity as mRNAs. On the one hand, they share the same transcription mechanism with mRNAs.[89,90] On the other hand, there is evidence that they have important regulatory functions.[31–34] It has been found that dosage-dependent regulators are usually transcription factors, signal transduction components, and chromatin proteins, which have in common that they are members of macromolecular complexes or play roles in multiple interactions.[18] Among these regulators, the most clearly studied one is *Inverse regulator-a* (*Inr-a* or *pcf11*), a bridge between RNA polymerase II and nascent transcripts, whose gene copy-number variations will perfectly mimic aneuploid effects.[64,91,92] Additional evidence suggests that in the fractionation after whole-genome duplication of eukaryotes, genes that participate in macromolecular complexes and are highly connected in the interactome are preferentially retained for longer periods, because deletion of one member of a duplicate pair may act like an aneuploid effect that is detrimental to genome balance.[21–23] In contrast, in small-scale duplications, genes of the same category are often underrepresented.[21,93] Genes whose products have more interactions with other molecules are more likely to be influenced by stoichiometry changes and participate in the regulatory networks in aneuploidy.

LncRNAs and TEs may act as dosage-sensitive regulators in imbalanced genomes. LncRNAs can have regulatory roles by interacting with proteins or other molecules.[32,90] For example, a large number of transcription factors interact with lncRNAs, which may be crucial for their functions.[94] Many lncRNAs are located on chromatin, and promote or inhibit the binding and activity of their interacting proteins at their targets.[28] Some lncRNAs are associated with chromatin remodeling complexes (such as polycomb repressive complexes PRC1 and PRC2), and further participate in chromatin remodeling and epigenetic regulation of gene expression through histone modifications.[90,95] In addition, lncRNAs can also form hybridization structures with DNA to influence chromatin accessibility,[28,90] or complementarily combine with pre-mRNA to affect mRNA splicing, editing, and stability.[90,96] Other lncRNAs can sponge miRNAs as competitive endogenous RNAs (ceRNAs), indirectly affecting the translation of mRNA.[28,90,97] With such a complex intermolecular interaction, lncRNAs are likely to be important in gene regulatory networks and susceptible to the disruption of stoichiometric balance. For TEs, their movement and accumulation may participate in the rewiring of the regulatory networks.[54] Studies have found that TEs form a large number of tissue-specific and alternative promoters in various species,[47] and nearly a quarter of the experimentally determined human promoters contain TE-derived sequences.[98] In mammals, TEs provide an average of 20% of binding sites for different transcription factors.[47] One-third of the transcription factors screened in human cells and tissues bind to the *L1* retrotransposon.[99] Furthermore, many regulatory RNAs, like some miRNAs and lncRNAs, are derived from TE sequences.[47,58,59] Other studies suggest that TEs can also be used as a source of ceRNAs, combining with miRNAs.[100] These complicated relationships may make TEs also dosage sensitive.

Through differential expression analysis, we identified many mRNAs, lncRNAs, and TE families affected by aneuploidy. The enriched functions of common DE-mRNAs explain the reduced viability of aneuploids, consistent with previous studies.[3,4,64,70] The functions of most DE-lncRNAs are unknown, so we performed co-expression and co-localization analysis with protein-coding genes to determine their potential interactors. We found that the lncRNA-mRNA co-expression network contains two large clusters, and their functions are predicted to be mainly related to microtubule-based processes (cluster 1) and proteasome-mediated protein catabolism (cluster 2). Several lncRNA-mRNA pairs in the co-expression network were selected for subsequent experimental verification. For the differentially expressed TE families, we also established a co-expression network with DE-mRNAs. The TE family with the most interacting mRNAs is *HMS-Beagle*, whose functions are predicted to be related to ubiquitin-dependent protein catabolic processes and responses to heat and biotic stimulus.

Evidence shows that the expression of lncRNAs is finely regulated and has higher tissue, cell type, developmental stage, and biological context specificity than mRNAs.[35,36] In *Drosophila*, approximately 30% of lncRNAs have the highest expression in testis,[101] and most lncRNAs studied in embryonic development have dynamic expression patterns[35] or complex subcellular localization.[102] Moreover, a large number of lncRNAs are significantly up-regulated at the beginning of metamorphosis in *Drosophila*, indicating that the enrichment of lncRNAs is critical for developmental transformation and organogenesis.[65,103] Similar to lncRNAs, TEs are often expressed in tissue and development stage-specific ways.[104,105] Highly conserved TE fragments in mammals tend to cluster around genes involved in development and transcriptional regulation.[106] A large number of developmentally regulated promoters derived from TEs are found in different species and drive the transcription of neighboring genes in a tissue or stage-specific fashion.[47] In addition, endogenous retroviruses (ERVs) are dynamically expressed in human and mouse embryonic development,[61,107] and some TEs of *Drosophila* can regulate early embryogenesis by inducing the degradation of maternal mRNAs through piRNAs produced by them.[108] Since both lncRNAs and TEs seem to have important developmental control functions, we also explored the roles of lncRNAs, TEs, and their interactors in the embryo development of aneuploid *Drosophila*.

We screened sixteen candidate genes from the differentially expressed lncRNA-mRNA pairs that may perform different types of functions, and an important TE family, and detected their expression patterns in aneuploid *Drosophila* embryos. The results showed that most candidate genes have specific subembryonic and subcellular localizations in three stages of embryogenesis, and some show different distributions in aneuploidy and wild type. Several candidate genes have interesting localization patterns. For example, the probe signals of *tsh*, which has multiple developmental and transcriptional regulatory functions, show segmented patterns in early embryogenesis; *HmgD*, which is related to transcription regulation and chromatin organization, is enriched in the nervous system in late embryonic development; *ifc*, which is related

to spermatogenesis, is enriched in pole cells of blastoderm. Both *nonA-l* (related to mRNA processing) and *lncRNA:CR45916* (involved in gene silencing) have intranuclear subcellular localization. Although the functions of most lncRNAs and TEs are unknown, candidate lncRNAs and TE have specific localization patterns in embryonic FISH. Compared with wild type, the distributions of several probes are changed in early trisomic embryos, indicating that these genes may contribute to the abnormal development of aneuploid *Drosophila*. Furthermore, almost all of the candidate genes have significant differences in relative fluorescence intensities between aneuploidy and wild-type embryos, and the directions are mostly consistent with the sequencing results. Through the analysis of RNA localization and relative expression, the potential relationship between lncRNAs and their paired mRNAs was confirmed. These results suggest that lncRNAs, TEs, and their interacting mRNAs can affect the development of aneuploid *Drosophila* embryos through spatiotemporal specific expression.

RNAi transgenic *Drosophila* lines were constructed to investigate the biological functions of some candidate lncRNAs and TE. We knocked down *lncRNA:CR33938*, *lncRNA:CR45916*, and the TE *HMS-Beagle* in *Drosophila* ovaries. By immunofluorescence staining, it was found that the relative quantity of SXL protein, whose enrichment at the top of germarium is required for GSC differentiation, was significantly reduced in the germarium in all three RNAi lines. Also, in mutant ovaries, the mitotic marker H3Ser10P tended to be distributed to later stage egg chambers, such as stages 6–7; whereas in control groups, H3Ser10P normally disappears in egg chambers at stage 6 when the follicle cells are entering the endocycle. Therefore, depletion of *lncRNA:CR33938*, *lncRNA:CR45916*, or *HMS-Beagle* may lead to abnormal ovarian development of *Drosophila*. Furthermore, the three genes all have co-expressed mRNAs with functions related to developmental regulation, and the interaction between them may be critical for their functions. The expression of *HMS-Beagle* is also affected by MSL2, which is implicated in *Drosophila* X chromosome expression. We also knocked down *lncRNA:CR44418* in *Drosophila* testes. It was predicted to have functions related to spermatogenesis and ubiquitination. We found that the ubiquitin signal was significantly down-regulated in mutant testes, confirming the relationship between *lncRNA:CR44418* and ubiquitination, and indicating that its reduction may affect male *Drosophila* fertility. These results all suggest that dosage-sensitive lncRNAs and TEs could play important biological functions.

In summary, our study provides a comprehensive picture of the characteristics and expression of lncRNAs and TEs in aneuploidy and demonstrates that lncRNAs and TEs, together with their interactors, are dosage-sensitive. Noncoding genes can also be involved in expression modulation and developmental control in aneuploid *Drosophila*. Moreover, based on the fact that aneuploidy is a feature of solid tumors[1,5] and that both lncRNAs and TEs are strongly associated to tumorigenesis,[42,49] studying the relationships among noncoding genes, unbalanced genomes, and aneuploidy-related human diseases may help to uncover the molecular mechanisms of disease and develop new therapies.

Limitations of the study

In this paper, through the study of aneuploid *Drosophila*, we showed that lncRNAs and TEs are dosage affected and strongly responsive to an inverse dosage effect. The sequencing data of some other species also support the prevalence of this response. Because this paper focuses on *Drosophila*, three additional aneuploidy datasets, *Arabidopsis*, mouse, and human cells, were added. With the widespread application of sequencing technology, it will be possible to find more resources of different taxa and different series of aneuploidy data on public database platforms, and further improve the dosage sensitivity study of noncoding genes across species. Although the *trans*-acting dosage effects in aneuploidy have been recognized for more than a century, the detailed molecular mechanism remains largely unknown. Dozens of dosage-dependent modifiers have been found in early studies, all of which are protein-coding genes. The lncRNAs and TEs mentioned in this study may be dosage-dependent modifiers, but more evidence is needed. Co-expression relationships are statements of the expression of lncRNAs or TEs and mRNAs in sequencing data but do not necessarily reflect regulatory relationships. In addition, lncRNAs and TEs have various ways to regulate the expression of other genes, which is also worth further exploration. We performed a preliminary study of the functions of several candidate lncRNAs and TE in *Drosophila* embryos and gonads by FISH and RNAi. More molecular biology experiments or studies at the cellular level are warranted.

STAR★METHODS

Detailed methods are provided in the online version of this paper and include the following:

- KEY RESOURCES TABLE
- RESOURCE AVAILABILITY
  - Lead contact
  - Materials availability
  - Data and code availability
- EXPERIMENTAL MODEL AND STUDY PARTICIPANT DETAILS
  - *Drosophila* stocks and genetic crosses
- METHOD DETAILS
  - Long noncoding RNAs (lncRNA) sequencing and data analysis
  - Data analysis of transposable elements (TEs)
  - Ratio distributions
  - Differential expression analysis and functional enrichment analysis
  - Screening of potential interactors of lncRNAs and TEs
  - Fluorescence *in situ* hybridization (FISH) of *Drosophila* embryos

- ○ Immunofluorescence of *Drosophila* ovaries and testes
- QUANTIFICATION AND STATISTICAL ANALYSIS

SUPPLEMENTAL INFORMATION

Supplemental information can be found online at https://doi.org/10.1016/j.isci.2023.108494.

ACKNOWLEDGMENTS

This research was supported by National Natural Science Foundation of China (Grant No. 31871243 and No. 32070566) to L.S. We are grateful to James A. Birchler for revising the manuscript. High-throughput sequencing services were performed by Novogene.

AUTHOR CONTRIBUTIONS

S.Z., R.W., and L.S. conceived the project, designed the experiments, undertook experiments, and wrote the manuscript; S.Z., R.W., X.Z., L.Z., and X.L. performed the experiments; S.Z. analyzed the data; S.Z., R.W., and L.S. wrote the manuscript. All authors have read and agreed to the published version of the manuscript.

DECLARATION OF INTERESTS

The authors declare no competing interests.

Received: July 2, 2023
Revised: September 28, 2023
Accepted: November 16, 2023
Published: November 19, 2023

REFERENCES

1. Orr, B., Godek, K.M., and Compton, D. (2015). Aneuploidy. Curr. Biol. *25*, R538–R542.
2. Williams, B.R., Prabhu, V.R., Hunter, K.E., Glazier, C.M., Whittaker, C.A., Housman, D.E., and Amon, A. (2008). Aneuploidy affects proliferation and spontaneous immortalization in mammalian cells. Science *322*, 703–709.
3. Torres, E.M., Sokolsky, T., Tucker, C.M., Chan, L.Y., Boselli, M., Dunham, M.J., and Amon, A. (2007). Effects of aneuploidy on cellular physiology and cell division in haploid yeast. Science *317*, 916–924.
4. Hwang, S., Cavaliere, P., Li, R., Zhu, L.J., Dephoure, N., and Torres, E.M. (2021). Consequences of aneuploidy in human fibroblasts with trisomy 21. Proc. Natl. Acad. Sci. USA *118*, e2014723118.
5. Torres, E.M., Williams, B.R., and Amon, A. (2008). Aneuploidy: cells losing their balance. Genetics *179*, 737–746.
6. Ambartsumyan, G., and Clark, A.T. (2008). Aneuploidy and early human embryo development. Hum. Mol. Genet. *17*, R10–R15.
7. García-Bellido, A., Delprado, J.M., and Botas, J. (1983). The Effect of Aneuploidy on Embryonic-Development in Drosophila-Melanogaster. Mol. Gen. Genet. *192*, 253–263.
8. Lécuyer, E., Yoshida, H., Parthasarathy, N., Alm, C., Babak, T., Cerovina, T., Hughes, T.R., Tomancak, P., and Krause, H.M. (2007). Global analysis of mRNA localization reveals a prominent role in organizing cellular architecture and function. Cell *131*, 174–187.
9. Braun, R., Ronquist, S., Wangsa, D., Chen, H., Anthuber, L., Gemoll, T., Wangsa, D., Koparde, V., Hunn, C., Habermann, J.K., et al. (2019). Single Chromosome Aneuploidy Induces Genome-Wide Perturbation of Nuclear Organization and Gene Expression. Neoplasia *21*, 401–412.
10. Hou, J., Shi, X., Chen, C., Islam, M.S., Johnson, A.F., Kanno, T., Huettel, B., Yen, M.R., Hsu, F.M., Ji, T., et al. (2018). Global impacts of chromosomal imbalance on gene expression in *Arabidopsis* and other taxa. Proc. Natl. Acad. Sci. USA *115*, E11321–E11330.
11. Shi, X., Yang, H., Chen, C., Hou, J., Hanson, K.M., Albert, P.S., Ji, T., Cheng, J., and Birchler, J.A. (2021). Genomic imbalance determines positive and negative modulation of gene expression in diploid maize. Plant Cell *33*, 917–939.
12. Sun, L., Johnson, A.F., Donohue, R.C., Li, J., Cheng, J., and Birchler, J.A. (2013). Dosage compensation and inverse effects in triple X metafemales of *Drosophila*. Proc. Natl. Acad. Sci. USA *110*, 7383–7388.
13. Sun, L., Johnson, A.F., Li, J., Lambdin, A.S., Cheng, J., and Birchler, J.A. (2013). Differential effect of aneuploidy on the X chromosome and genes with sex-biased expression in *Drosophila*. Proc. Natl. Acad. Sci. USA *110*, 16514–16519.
14. Prestel, M., Feller, C., and Becker, P.B. (2010). Dosage compensation and the global re-balancing of aneuploid genomes. Genome Biol. *11*, 216.
15. Birchler, J.A., and Veitia, R.A. (2010). The gene balance hypothesis: implications for gene regulation, quantitative traits and evolution. New Phytol. *186*, 54–62.
16. Birchler, J.A., and Veitia, R.A. (2012). Gene balance hypothesis: connecting issues of dosage sensitivity across biological disciplines. Proc. Natl. Acad. Sci. USA *109*, 14746–14753.
17. Birchler, J.A., Yao, H., and Chudalayandi, S. (2007). Biological consequences of dosage dependent gene regulatory systems. Biochim. Biophys. Acta *1769*, 422–428.
18. Birchler, J.A., Bhadra, U., Bhadra, M.P., and Auger, D.L. (2001). Dosage-dependent gene regulation in multicellular eukaryotes: implications for dosage compensation, aneuploid syndromes, and quantitative traits. Dev. Biol. *234*, 275–288.
19. Veitia, R.A., Bottani, S., and Birchler, J.A. (2008). Cellular reactions to gene dosage imbalance: genomic, transcriptomic and proteomic effects. Trends Genet. *24*, 390–397.
20. Veitia, R.A., Bottani, S., and Birchler, J.A. (2013). Gene dosage effects: nonlinearities, genetic interactions, and dosage compensation. Trends Genet. *29*, 385–393.
21. Freeling, M. (2009). Bias in plant gene content following different sorts of duplication: tandem, whole-genome, segmental, or by transposition. Annu. Rev. Plant Biol. *60*, 433–453.
22. Blanc, G., and Wolfe, K.H. (2004). Functional divergence of duplicated genes formed by polyploidy during *Arabidopsis* evolution. Plant Cell *16*, 1679–1691.
23. Birchler, J.A., and Veitia, R.A. (2007). The gene balance hypothesis: from classical genetics to modern genomics. Plant Cell *19*, 395–402.
24. Disteche, C.M. (2016). Dosage compensation of the sex chromosomes and autosomes. Semin. Cell Dev. Biol. *56*, 9–18.
25. Shi, X., Yang, H., Chen, C., Hou, J., Ji, T., Cheng, J., and Birchler, J.A. (2022). Dosage-sensitive miRNAs trigger modulation of gene expression during genomic imbalance in maize. Nat. Commun. *13*, 3014.
26. Rogoyski, O.M., Pueyo, J.I., Couso, J.P., and Newbury, S.F. (2017). Functions of long non-coding RNAs in human disease and their

conservation in *Drosophila* development. Biochem. Soc. Trans. *45*, 895–904.
27. Li, K., Tian, Y., Yuan, Y., Fan, X., Yang, M., He, Z., and Yang, D. (2019). Insights into the Functions of LncRNAs in *Drosophila*. Int. J. Mol. Sci. *20*, 4646.
28. Statello, L., Guo, C.J., Chen, L.L., and Huarte, M. (2021). Gene regulation by long non-coding RNAs and its biological functions. Nat. Rev. Mol. Cell Biol. *22*, 96–118.
29. Wapinski, O., and Chang, H.Y. (2011). Long noncoding RNAs and human disease. Trends Cell Biol. *21*, 354–361.
30. Kung, J.T.Y., Colognori, D., and Lee, J.T. (2013). Long noncoding RNAs: past, present, and future. Genetics *193*, 651–669.
31. Wang, K.C., and Chang, H.Y. (2011). Molecular mechanisms of long noncoding RNAs. Mol. Cell. *43*, 904–914.
32. Kopp, F., and Mendell, J.T. (2018). Functional Classification and Experimental Dissection of Long Noncoding RNAs. Cell *172*, 393–407.
33. Hongay, C.F., Grisafi, P.L., Galitski, T., and Fink, G.R. (2006). Antisense transcription controls cell fate in *Saccharomyces cerevisiae*. Cell *127*, 735–745.
34. Groff, A.F., Sanchez-Gomez, D.B., Soruco, M.M.L., Gerhardinger, C., Barutcu, A.R., Li, E., Elcavage, L., Plana, O., Sanchez, L.V., Lee, J.C., et al. (2016). In Vivo Characterization of *Linc-p21* Reveals Functional *cis*-Regulatory DNA Elements. Cell Rep. *16*, 2178–2186.
35. Schor, I.E., Bussotti, G., Males, M., Forneris, M., Viales, R.R., Enright, A.J., and Furlong, E.E.M. (2018). Non-coding RNA Expression, Function, and Variation during *Drosophila* Embryogenesis. Curr. Biol. *28*, 3547–3561.e3549.
36. Cabili, M.N., Trapnell, C., Goff, L., Koziol, M., Tazon-Vega, B., Regev, A., and Rinn, J.L. (2011). Integrative annotation of human large intergenic noncoding RNAs reveals global properties and specific subclasses. Genes Dev. *25*, 1915–1927.
37. Dhanoa, J.K., Sethi, R.S., Verma, R., Arora, J.S., and Mukhopadhyay, C.S. (2018). Long non-coding RNA: its evolutionary relics and biological implications in mammals: a review. J. Anim. Sci. Technol. *60*, 25.
38. ENCODE Project Consortium, Birney, E., Stamatoyannopoulos, J.A., Dutta, A., Guigó, R., Gingeras, T.R., Margulies, E.H., Weng, Z., Snyder, M., Dermitzakis, E.T., et al. (2007). Identification and analysis of functional elements in 1% of the human genome by the ENCODE pilot project. Nature *447*, 799–816.
39. Whitehead, J., Pandey, G.K., and Kanduri, C. (2009). Regulation of the mammalian epigenome by long noncoding RNAs. Biochim. Biophys. Acta *1790*, 936–947.
40. Clemson, C.M., Hutchinson, J.N., Sara, S.A., Ensminger, A.W., Fox, A.H., Chess, A., and Lawrence, J.B. (2009). An architectural role for a nuclear noncoding RNA: *NEAT1* RNA is essential for the structure of paraspeckles. Mol. Cell. *33*, 717–726.
41. Pauli, A., Rinn, J.L., and Schier, A.F. (2011). Non-coding RNAs as regulators of embryogenesis. Nat. Rev. Genet. *12*, 136–149.
42. Schmitt, A.M., and Chang, H.Y. (2016). Long Noncoding RNAs in Cancer Pathways. Cancer Cell *29*, 452–463.
43. Wells, J.N., and Feschotte, C. (2020). A Field Guide to Eukaryotic Transposable Elements. Annu. Rev. Genet. *54*, 539–561.
44. Schnable, P.S., Ware, D., Fulton, R.S., Stein, J.C., Wei, F., Pasternak, S., Liang, C., Zhang, J., Fulton, L., Graves, T.A., et al. (2009). The B73 maize genome: complexity, diversity, and dynamics. Science *326*, 1112–1115.
45. Slotkin, R.K., and Martienssen, R. (2007). Transposable elements and the epigenetic regulation of the genome. Nat. Rev. Genet. *8*, 272–285.
46. Mérel, V., Boulesteix, M., Fablet, M., and Vieira, C. (2020). Transposable elements in *Drosophila*. Mobile DNA *11*, 23.
47. Chuong, E.B., Elde, N.C., and Feschotte, C. (2017). Regulatory activities of transposable elements: from conflicts to benefits. Nat. Rev. Genet. *18*, 71–86.
48. Hancks, D.C., and Kazazian, H.H., Jr. (2016). Roles for retrotransposon insertions in human disease. Mobile DNA *7*, 9.
49. Lee, E., Iskow, R., Yang, L., Gokcumen, O., Haseley, P., Luquette, L.J., 3rd, Lohr, J.G., Harris, C.C., Ding, L., Wilson, R.K., et al. (2012). Landscape of somatic retrotransposition in human cancers. Science *337*, 967–971.
50. Bourgeois, Y., and Boissinot, S. (2019). On the Population Dynamics of Junk: A Review on the Population Genomics of Transposable Elements. Genes *10*, 419.
51. Moran, J.V., DeBerardinis, R.J., and Kazazian, H.H. (1999). Exon shuffling by L1 retrotransposition. Science *283*, 1530–1534.
52. Moschetti, R., Marsano, R.M., Barsanti, P., Caggese, C., and Caizzi, R. (2004). FB elements can promote exon shuffling: a promoter-less white allele can be reactivated by FB mediated transposition in Drosophila melanogaster. Mol. Genet. Genom. *271*, 394–401.
53. Cáceres, M., Puig, M., and Ruiz, A. (2001). Molecular characterization of two natural hotspots in the Drosophila buzzatii genome induced by transposon insertions. Genome Res. *11*, 1353–1364.
54. Feschotte, C. (2008). Transposable elements and the evolution of regulatory networks. Nat. Rev. Genet. *9*, 397–405.
55. Roseman, R.R., Swan, J.M., and Geyer, P.K. (1995). A *Drosophila* insulator protein facilitates dosage compensation of the X chromosome *min-white* gene located at autosomal insertion sites. Development *121*, 3573–3582.
56. Bejerano, G., Lowe, C.B., Ahituv, N., King, B., Siepel, A., Salama, S.R., Rubin, E.M., Kent, W.J., and Haussler, D. (2006). A distal enhancer and an ultraconserved exon are derived from a novel retroposon. Nature *441*, 87–90.
57. Guio, L., Vieira, C., and González, J. (2018). Stress affects the epigenetic marks added by natural transposable element insertions in *Drosophila melanogaster*. Sci. Rep. *8*, 12197.
58. Kapusta, A., Kronenberg, Z., Lynch, V.J., Zhuo, X., Ramsay, L., Bourque, G., Yandell, M., and Feschotte, C. (2013). Transposable elements are major contributors to the origin, diversification, and regulation of vertebrate long noncoding RNAs. PLoS Genet. *9*, e1003470.
59. Piriyapongsa, J., Mariño-Ramírez, L., and Jordan, I.K. (2007). Origin and evolution of human microRNAs from transposable elements. Genetics *176*, 1323–1337.
60. Lynch, V.J., Leclerc, R.D., May, G., and Wagner, G.P. (2011). Transposon-mediated rewiring of gene regulatory networks contributed to the evolution of pregnancy in mammals. Nat. Genet. *43*, 1154–1159.
61. Gerdes, P., Richardson, S.R., Mager, D.L., and Faulkner, G.J. (2016). Transposable elements in the mammalian embryo: pioneers surviving through stealth and service. Genome Biol. *17*, 100.
62. Ellison, C.E., and Bachtrog, D. (2013). Dosage compensation via transposable element mediated rewiring of a regulatory network. Science *342*, 846–850.
63. Zhang, S., Qi, H., Huang, C., Yuan, L., Zhang, L., Wang, R., Tian, Y., and Sun, L. (2021). Interaction of Male Specific Lethal complex and genomic imbalance on global gene expression in *Drosophila*. Sci. Rep. *11*, 19679.
64. Zhang, S., Wang, R., Huang, C., Zhang, L., and Sun, L. (2021). Modulation of Global Gene Expression by Aneuploidy and CNV of Dosage Sensitive Regulatory Genes. Genes *12*, 1606.
65. Camilleri-Robles, C., Amador, R., Klein, C.C., Guigó, R., Corominas, M., and Ruiz-Romero, M. (2022). Genomic and functional conservation of lncRNAs: lessons from flies. Mamm. Genome *33*, 328–342.
66. Zhang, L., Zhang, S., Wang, R., and Sun, L. (2022). Genome-Wide Identification of Long Noncoding RNA and Their Potential Interactors in *ISWI* Mutants. Int. J. Mol. Sci. *23*, 6247.
67. Sarropoulos, I., Marin, R., Cardoso-Moreira, M., and Kaessmann, H. (2019). Developmental dynamics of lncRNAs across mammalian organs and species. Nature *571*, 510–514.
68. Treiber, C.D., and Waddell, S. (2020). Transposon expression in the *Drosophila* brain is driven by neighboring genes and diversifies the neural transcriptome. Genome Res. *30*, 1559–1569.
69. Rahman, R., Chirn, G.W., Kanodia, A., Sytnikova, Y.A., Brembs, B., Bergman, C.M., and Lau, N.C. (2015). Unique transposon landscapes are pervasive across *Drosophila melanogaster* genomes. Nucleic Acids Res. *43*, 10655–10672.
70. Tsai, H.J., Nelliat, A.R., Choudhury, M.I., Kucharavy, A., Bradford, W.D., Cook, M.E., Kim, J., Mair, D.B., Sun, S.X., Schatz, M.C., and Li, R. (2019). Hypo-osmotic-like stress underlies general cellular defects of aneuploidy. Nature *570*, 117–121.
71. Salz, H.K. (2013). Sex, stem cells and tumors in the *Drosophila* ovary. Fly *7*, 3–7.
72. Chau, J., Kulnane, L.S., and Salz, H.K. (2009). Sex-lethal Facilitates the Transition From Germline Stem Cell to Committed Daughter Cell in the *Drosophila* Ovary. Genetics *182*, 121–132.
73. Jia, D., Xu, Q., Xie, Q., Mio, W., and Deng, W.M. (2016). Automatic stage identification of *Drosophila* egg chamber based on DAPI images. Sci. Rep. *6*, 18850.
74. Rathke, C., Baarends, W.M., Jayaramaiah-Raja, S., Bartkuhn, M., Renkawitz, R., and Renkawitz-Pohl, R. (2007). Transition from a nucleosome-based to a protamine-based chromatin configuration during spermiogenesis in *Drosophila*. J. Cell Sci. *120*, 1689–1700.
75. Nakamura, N. (2013). Ubiquitination Regulates the Morphogenesis and Function of Sperm Organelles. Cells *2*, 732–750.
76. Mao, R., Zielke, C.L., Zielke, H.R., and Pevsner, J. (2003). Global up-regulation of chromosome 21 gene expression in the

developing Down syndrome brain. Genomics *81*, 457–467.
77. Yang, H., Shi, X., Chen, C., Hou, J., Ji, T., Cheng, J., and Birchler, J.A. (2021). Predominantly inverse modulation of gene expression in genomically unbalanced disomic haploid maize. Plant Cell *33*, 901–916.
78. Zhang, X., Hong, D., Ma, S., Ward, T., Ho, M., Pattni, R., Duren, Z., Stankov, A., Bade Shrestha, S., Hallmayer, J., et al. (2020). Integrated functional genomic analyses of Klinefelter and Turner syndromes reveal global network effects of altered X chromosome dosage. Proc. Natl. Acad. Sci. USA *117*, 4864–4873.
79. Raznahan, A., Parikshak, N.N., Chandran, V., Blumenthal, J.D., Clasen, L.S., Alexander-Bloch, A.F., Zinn, A.R., Wangsa, D., Wise, J., Murphy, D.G.M., et al. (2018). Sex-chromosome dosage effects on gene expression in humans. Proc. Natl. Acad. Sci. USA *115*, 7398–7403.
80. Nawata, H., Kashino, G., Tano, K., Daino, K., Shimada, Y., Kugoh, H., Oshimura, M., and Watanabe, M. (2011). Dysregulation of gene expression in the artificial human trisomy cells of chromosome 8 associated with transformed cell phenotypes. PLoS One *6*, e25319.
81. Birchler, J.A., and Veitia, R.A. (2021). One Hundred Years of Gene Balance: How Stoichiometric Issues Affect Gene Expression, Genome Evolution, and Quantitative Traits. Cytogenet. Genome Res. *161*, 529–550.
82. Birchler, J.A. (1979). A study of enzyme activities in a dosage series of the long arm of chromosome one in maize. Genetics *92*, 1211–1229.
83. Johnson, A.F., Hou, J., Yang, H., Shi, X., Chen, C., Islam, M.S., Ji, T., Cheng, J., and Birchler, J.A. (2020). Magnitude of modulation of gene expression in aneuploid maize depends on the extent of genomic imbalance. J Genet Genomics *47*, 93–103.
84. Sun, L., Fernandez, H.R., Donohue, R.C., Li, J., Cheng, J., and Birchler, J.A. (2013). Male-specific lethal complex in *Drosophila* counteracts histone acetylation and does not mediate dosage compensation. Proc. Natl. Acad. Sci. USA *110*, E808–E817.
85. Birchler, J.A., Pal-Bhadra, M., and Bhadra, U. (2003). Dosage dependent gene regulation and the compensation of the X chromosome in *Drosophila* males. Genetica *117*, 179–190.
86. Birchler, J.A., Owenby, R.K., and Jacobson, K.B. (1982). Dosage compensation of serine-4 transfer RNA in *Drosophila melanogaster*. Genetics *102*, 525–537.
87. Hiebert, J.C., and Birchler, J.A. (1992). Dosage compensation of the *copia* retrotransposon in *Drosophila melanogaster*. Genetics *130*, 539–545.
88. Shi, X., Yang, H., and Birchler, J.A. (2023). MicroRNAs play regulatory roles in genomic balance. Bioessays *45*, e2200187.
89. Quinn, J.J., and Chang, H.Y. (2016). Unique features of long non-coding RNA biogenesis and function. Nat. Rev. Genet. *17*, 47–62.
90. Kazimierczyk, M., Kasprowicz, M.K., Kasprzyk, M.E., and Wrzesinski, J. (2020). Human Long Noncoding RNA Interactome: Detection, Characterization and Function. Int. J. Mol. Sci. *21*, 1027.
91. Rabinow, L., Nguyen-Huynh, A.T., and Birchler, J.A. (1991). A *trans*-acting regulatory gene that inversely affects the expression of the *white, brown* and *scarlet* loci in *Drosophila*. Genetics *129*, 463–480.
92. Xie, W., and Birchler, J.A. (2012). Identification of Inverse Regulator-a (*Inr-a*) as Synonymous with Pre-mRNA Cleavage Complex II Protein (*Pcf11*) in *Drosophila*. G3 (Bethesda) *2*, 701–706.
93. Birchler, J.A. (2012). Insights from paleogenomic and population studies into the consequences of dosage sensitive gene expression in plants. Curr. Opin. Plant Biol. *15*, 544–548.
94. Long, Y., Wang, X., Youmans, D.T., and Cech, T.R. (2017). How do lncRNAs regulate transcription? Sci. Adv. *3*, eaao2110.
95. Gupta, R.A., Shah, N., Wang, K.C., Kim, J., Horlings, H.M., Wong, D.J., Tsai, M.C., Hung, T., Argani, P., Rinn, J.L., et al. (2010). Long non-coding RNA *HOTAIR* reprograms chromatin state to promote cancer metastasis. Nature *464*, 1071–1076.
96. Romero-Barrios, N., Legascue, M.F., Benhamed, M., Ariel, F., and Crespi, M. (2018). Splicing regulation by long noncoding RNAs. Nucleic Acids Res. *46*, 2169–2184.
97. Salmena, L., Poliseno, L., Tay, Y., Kats, L., and Pandolfi, P.P. (2011). A ceRNA hypothesis: the Rosetta Stone of a hidden RNA language? Cell *146*, 353–358.
98. Jordan, I.K., Rogozin, I.B., Glazko, G.V., and Koonin, E.V. (2003). Origin of a substantial fraction of human regulatory sequences from transposable elements. Trends Genet. *19*, 68–72.
99. Sun, X., Wang, X., Tang, Z., Grivainis, M., Kahler, D., Yun, C., Mita, P., Fenyö, D., and Boeke, J.D. (2018). Transcription factor profiling reveals molecular choreography and key regulators of human retrotransposon expression. Proc. Natl. Acad. Sci. USA *115*, E5526–E5535.
100. Cho, J., and Paszkowski, J. (2017). Regulation of rice root development by a retrotransposon acting as a microRNA sponge. Elife *6*, e30038.
101. Brown, J.B., Boley, N., Eisman, R., May, G.E., Stoiber, M.H., Duff, M.O., Booth, B.W., Wen, J., Park, S., Suzuki, A.M., et al. (2014). Diversity and dynamics of the *Drosophila* transcriptome. Nature *512*, 393–399.
102. Wilk, R., Hu, J., Blotsky, D., and Krause, H.M. (2016). Diverse and pervasive subcellular distributions for both coding and long noncoding RNAs. Genes Dev. *30*, 594–609.
103. Chen, B., Zhang, Y., Zhang, X., Jia, S., Chen, S., and Kang, L. (2016). Genome-wide identification and developmental expression profiling of long noncoding RNAs during *Drosophila* metamorphosis. Sci. Rep. *6*, 23330.
104. Faulkner, G.J., Kimura, Y., Daub, C.O., Wani, S., Plessy, C., Irvine, K.M., Schroder, K., Cloonan, N., Steptoe, A.L., Lassmann, T., et al. (2009). The regulated retrotransposon transcriptome of mammalian cells. Nat. Genet. *41*, 563–571.
105. Djebali, S., Davis, C.A., Merkel, A., Dobin, A., Lassmann, T., Mortazavi, A., Tanzer, A., Lagarde, J., Lin, W., Schlesinger, F., et al. (2012). Landscape of transcription in human cells. Nature *489*, 101–108.
106. Lowe, C.B., Bejerano, G., and Haussler, D. (2007). Thousands of human mobile element fragments undergo strong purifying selection near developmental genes. Proc. Natl. Acad. Sci. USA *104*, 8005–8010.
107. Grow, E.J., Flynn, R.A., Chavez, S.L., Bayless, N.L., Wossidlo, M., Wesche, D.J., Martin, L., Ware, C.B., Blish, C.A., Chang, H.Y., et al. (2015). Intrinsic retroviral reactivation in human preimplantation embryos and pluripotent cells. Nature *522*, 221–225.
108. Rouget, C., Papin, C., Boureux, A., Meunier, A.C., Franco, B., Robine, N., Lai, E.C., Pelisson, A., and Simonelig, M. (2010). Maternal mRNA deadenylation and decay by the piRNA pathway in the early *Drosophila* embryo. Nature *467*, 1128–1132.
109. Kim, D., Paggi, J.M., Park, C., Bennett, C., and Salzberg, S.L. (2019). Graph-based genome alignment and genotyping with HISAT2 and HISAT-genotype. Nat. Biotechnol. *37*, 907–915.
110. Pertea, M., Pertea, G.M., Antonescu, C.M., Chang, T.C., Mendell, J.T., and Salzberg, S.L. (2015). StringTie enables improved reconstruction of a transcriptome from RNA-seq reads. Nat. Biotechnol. *33*, 290–295.
111. Villanueva, R.A.M., and Chen, Z.J. (2019). ggplot2: Elegant Graphics for Data Analysis. Meas-Interdiscip Res *17*, 160–167.
112. Love, M.I., Huber, W., and Anders, S. (2014). Moderated estimation of fold change and dispersion for RNA-seq data with DESeq2. Genome Biol. *15*, 550.
113. Wu, T., Hu, E., Xu, S., Chen, M., Guo, P., Dai, Z., Feng, T., Zhou, L., Tang, W., Zhan, L., et al. (2021). clusterProfiler 4.0: A universal enrichment tool for interpreting omics data. Innovation *2*, 100141.
114. Shannon, P., Markiel, A., Ozier, O., Baliga, N.S., Wang, J.T., Ramage, D., Amin, N., Schwikowski, B., and Ideker, T. (2003). Cytoscape: A software environment for integrated models of biomolecular interaction networks. Genome Res. *13*, 2498–2504.
115. Wicker, T., Sabot, F., Hua-Van, A., Bennetzen, J.L., Capy, P., Chalhoub, B., Flavell, A., Leroy, P., Morgante, M., Panaud, O., et al. (2007). A unified classification system for eukaryotic transposable elements. Nat. Rev. Genet. *8*, 973–982.
116. Li, H., Handsaker, B., Wysoker, A., Fennell, T., Ruan, J., Homer, N., Marth, G., Abecasis, G., and Durbin, R.; 1000 Genome Project Data Processing Subgroup (2009). The Sequence Alignment/Map format and SAMtools. Bioinformatics *25*, 2078–2079.
117. R Core Team (2022). R: A Language and Environment for Statistical Computing (R Foundation for Statistical Computing).
118. Gu, Z., Eils, R., and Schlesner, M. (2016). Complex heatmaps reveal patterns and correlations in multidimensional genomic data. Bioinformatics *32*, 2847–2849.
119. Jandura, A., Hu, J., Wilk, R., and Krause, H.M. (2017). High Resolution Fluorescent *In Situ* Hybridization in *Drosophila* Embryos and Tissues Using Tyramide Signal Amplification. J. Vis. Exp. *128*, 56281.

CellPress
OPEN ACCESS

STAR★METHODS

KEY RESOURCES TABLE

REAGENT or RESOURCE	SOURCE	IDENTIFIER
**Antibodies**		
Rabbit anti-H3Ser10P	EMD Millipore	Cat#06-570; RRID: AB_310177
Mouse anti-SXL	Developmental Studies Hybridoma Bank	Cat#M18-s; RRID: AB_528464
Mouse anti-Ubiquitin	Santa Cruz Biotechnology	Cat#sc-8017; RRID: AB_628423
**Deposited data**		
lncRNA sequencing data of wildtype and aneuploid *Drosophila*	This paper	GEO: GSE233534
RNA-seq data of MSL2 overexpressed *Drosophila*	Gene Expression Omnibus	GEO: GSE41570
RNA-seq data of *Arabidopsis* aneuploidy	Gene Expression Omnibus	GEO: GSE79676
RNA-seq data of mouse aneuploidy	Gene Expression Omnibus	GEO: GSE179435
RNA-seq data of human aneuploidy	Gene Expression Omnibus	GEO: GSE203257
**Experimental models: Organisms/strains**		
D. melanogaster: *y; C(2L)dp; F(2R) bw*	This paper	N/A
D. melanogaster: *C(1)DX, ywf/winscy*	This paper	N/A
D. melanogaster: *UAS-lncRNA:CR33938-RNAi*	This paper	TH15945.S
D. melanogaster: *UAS-lncRNA:CR45916-RNAi*	This paper	TH15947.S
D. melanogaster: *UAS-lncRNA:CR44418-RNAi*	This paper	TH15951.S
D. melanogaster: *UAS-HMS-beagle-RNAi*	This paper	TH16030.S
D. melanogaster: *w;GawB-bab1[Pgal4-2]/TM6B*	Qidong Fangjing Biotechnology	OCB00568
D. melanogaster: *GawB-C587,w*	Qidong Fangjing Biotechnology	OCB02697
**Oligonucleotides**		
Primers for TSA-FISH, see Figure S10	This paper	N/A
RNAi targeting sequence, see experimental model and study participant details	This paper	N/A
**Software and algorithms**		
HISAT2	Kim et al., 2019[109]	http://daehwankimlab.github.io/hisat2/
StringTie	Pertea et al., 2015[110]	https://ccb.jhu.edu/software/stringtie/
R	The R Project for Statistical Computing	https://www.r-project.org/
ggplot2	Villanueva et al., 2019[111]	https://cran.r-project.org/web/packages/ggplot2/index.html
DESeq2	Love et al., 2014[112]	https://www.bioconductor.org/packages/DESeq2
ClusterProfiler	Wu et al., 2021[113]	https://www.bioconductor.org/packages/clusterProfiler
Cytoscape	Cline et al., 2003[114]	http://www.cytoscape.org
ImageJ	Schindelin et al., 2019	https://fiji.sc/

RESOURCE AVAILABILITY

Lead contact

Further information and requests for resources and reagents should be directed to and will be fulfilled by the lead contact, Lin Sun (sunlin@bnu.edu.cn).

Materials availability

The *Drosophila* strains used in this study can be purchased from TsingHua Fly Center and Qidong Fangjing Biotechnology.

Data and code availability

- The sequencing data generated in this study have been deposited in the Gene Expression Omnibus (GEO) database and are accessible through GEO Series accession number GSE233534. The previously published datasets of *Arabidopsis*, mouse, and human aneuploidy used in this article were downloaded from the GEO repository under the accession number GSE79676, GSE179435, and GSE203257, respectively. RNA-seq data of MSL2 overexpressed *Drosophila* are available at the GEO repository under the accession number GSE41570. These datasets are all publicly available. The sources of the datasets supporting the current study are presented in the "key resources table" and "STAR methods" sections.
- This paper does not report the original code.
- Any additional information required to reanalyze the data reported in this work paper is available from the lead contact upon request.

EXPERIMENTAL MODEL AND STUDY PARTICIPANT DETAILS

Drosophila stocks and genetic crosses

The *Drosophila* strains used in this study were all maintained or crossed in our laboratory. The crossing scheme to obtain aneuploid *Drosophila* has been described previously.[63] Trisomy chromosome 2 left arm (2L) female and male third-instar larvae were obtained from the cross of *y; C(2L)dp; F(2R) bw* females and wildtype males. The metafemale larvae were screened from the filial generation of the cross of *C(1)DX, ywf/winscy* females and wildtype males. All *Drosophila* strains were cultured on cornmeal dextrose medium at 25°C. Genes and chromosomal balancers are described in Flybase (https://flybase.org/).

The UAS-RNAi *Drosophila* were constructed by TsingHua Fly Center (https://thfc.zzbd.org/faq.html). First, the designed short hairpin RNA (shRNA) of the target gene was integrated into the pNP vector. After purification, the pNP-shRNA vector was injected into *Drosophila* embryos with a background genotype of *y[1] sc[1] v[1] p{y[+t7.7]}=nos-phiC31\int.NLS}X; P{y[+t7.7]=CaryP}attP2* (TB00018). The filial generation were selected according to their phenotypes and crossed, and finally transgenic *Drosophila* were obtained. The UAS-RNAi strains involved in this paper include: *UAS-lncRNA:CR33938-RNAi* (TH15945.S) with target sequence CACGAGGACCAAACCAGCGAA inserted at attP2 site; *UAS-lncRNA:CR45916-RNAi* (TH15947.S) with target sequence ATGGCACAATGTGATTAGCAA inserted at attP2 site; *UAS-lncRNA:CR44418-RNAi* (TH15951.S) with target sequence ACCAAAGTTATTAGTCCGCAA inserted at attP2 site; *UAS-HMS-beagle-RNAi* (TH16030.S) with target sequence TACGCTAGATTTAGTTTCAAA inserted at attP2 site. The GAL4 driver lines were obtained from Qidong Fangjing Biotechnology (http://fungene.tech/). Crossing *w;GawB-bab1[Pgal4-2]/TM6B* (OCB00568) with UAS-RNAi strains results in specific knockdown of the target gene in the ovary. *GawB-C587,w* (OCB02697) could knock down target genes in the testes and ovaries when crossed to the UAS-RNAi lines.

METHOD DETAILS

Long noncoding RNAs (lncRNA) sequencing and data analysis

The third-instar larvae of wildtype and aneuploid *Drosophila* were collected as samples for sequencing. LncRNA sequencing was performed by Novogene. Firstly, total RNA was extracted from *Drosophila* tissues, and its concentration, purity and integrity were determined. The ribosomal RNA was then removed from RNA samples that passed quality control using the rRNA Removal Kit, and a strand-specific library was constructed. Finally, the sequencing was performed using Illumina NovaSeq 6000 sequencers with the paired-end 150 bp protocol.

The raw data obtained from RNA sequencing was first processed by Trim Galore (version 0.6.7) (https://www.bioinformatics.babraham.ac.uk/projects/trim_galore/) to remove adapters and low-quality bases, and the quality of the data was detected by FastQC (version 0.11.9) (https://www.bioinformatics.babraham.ac.uk/projects/fastqc/). Subsequently, clean reads were aligned to the *Drosophila* reference genome (Drosophila_melanogaster.BDGP6.32.dna.toplevel.fa, downloaded from the Ensembl database) using HISAT2 (version 2.2.1).[109] Next, the alignment results were assembled and quantitated using StringTie (version 2.1.5).[110] The transcript_biotype item in the corresponding genome annotation (version 6.32.105) was used to distinguish between protein-coding genes and noncoding genes to obtain their read counts.

Data analysis of transposable elements (TEs)

Since TEs are part of the repetitive fraction of the genome,[54] it will naturally lead to the phenomenon that one read matches multiple positions in the reference genome. In addition, the similarity of TEs in the same family makes it difficult to distinguish different TE insertions in the same family.[115] To address this technical limitation of sequencing and to simplify the complexity of TE expression, we filtered out secondary multiple alignments in the results of HISAT2 and only counted the total number of reads that mapped to the insertion sequences of each TE

CellPress
OPEN ACCESS

family. Specifically, samtools (version 1.15)[116] was used to process the bam files after HISAT2 alignment, and the multiple alignments were removed (samtools view -h -b -q 1 -F 256). Then, the filtered results were reassembled and quantified by using StringTie. TEs were selected from the genome annotation and the read counts of TE insertions of the same family were added to obtain the total expression of a TE family.

Ratio distributions

The method of making the ratio distributions of transcript expression changes is as described previously.[63] The expression of protein-coding genes and lncRNA genes were normalized to CPM (counts per million). The expression of each TE family was normalized with the overall expression of TEs. Low-expression transcripts were filtered to retain only those with mean read counts greater than 5. Subsequently, the ratios of expression levels of different types of transcripts between aneuploidy and diploid were calculated, respectively. These ratios were plotted as distributions in bins of 0.1, with the vertical axis showing the percentage of the number of transcripts contained in each interval to the total number of transcripts of this type. The plots were generated using ggplot2 (version 3.3.6)[114] in the R program (version 4.2.1).[117]

Differential expression analysis and functional enrichment analysis

Differential expression analysis was performed for protein-coding genes and noncoding genes with mean read counts greater than 5 in each sample using R package DESeq2 (version 1.36.0).[112] Adjusted p value <0.1 was set as the threshold for significantly different expression. The heatmaps were generated using ComplexHeatmap (version 2.12.1).[118] Enrichment analysis was performed by ClusterProfiler (version 4.4.4)[113] using the annotation package org.Dm.e.g.,.db (version 3.15.0) from Bioconductor (https://www.bioconductor.org/). The KEGG pathway data was obtained from network (https://www.kegg.jp/, accessed on 27 August 2022). The differential expression of TE family was analyzed separately.

Screening of potential interactors of lncRNAs and TEs

For differentially expressed lncRNAs (DE-lncRNAs) or TEs (DE-TEs), we screened their possible interacting genes according to co-localization or co-expression. To find mRNAs that were highly related to the expression pattern of the DE-lncRNAs, we calculated the Pearson correlation coefficient between the DE-lncRNAs and the differentially expressed mRNAs (DE-mRNAs). LncRNA-mRNA pairs with absolute values of correlation coefficient >0.95 and p value <0.05 were considered to have interactions. In addition, there may be *cis* interactions between lncRNA and mRNA with adjacent genome coordinates. Therefore, the DE-mRNAs within 10 kb upstream and downstream of the DE-lncRNAs was found by bedtools to form co-located lncRNA-mRNA pairs. We used the mRNAs in these gene pairs to speculate the function of the corresponding lncRNA. The interaction networks of lncRNA or TE and mRNA were drawn using Cytoscape (version 3.7.1).[114]

Fluorescence *in situ* hybridization (FISH) of *Drosophila* embryos

TSA-FISH of *Drosophila* embryos was performed as described.[63] In brief, the embryos of three different developmental stages from wildtype and aneuploid *Drosophila* were first collected according to the corresponding time window, fixed with formaldehyde and stored in methanol at −20°C. Next, primers containing flanking T7 promoter elements are designed for candidate genes (Figure S10A). After PCR amplification and *in vitro* transcription, antisense RNA probes containing digoxigenin-labeled UTP were obtained. The probes were tested by agarose gel electrophoresis (Figure S10B). Subsequently, the embryos were permeabilization, pre-hybridization, and hybridization according to the previously published protocol.[119] Probe signals were detected using tyramide signal amplification (TSA) signal amplification system. Finally, *Drosophila* embryos were placed in anti-fade mounting medium to make slides, and observed with fluorescence microscope (Zeiss Investigated Fluorescence Microcopy Observer Z1) and confocal microscope (ZEISS LSM880). The same probes of different genotype samples for relative fluorescence intensity analysis were photographed using the same parameters. Fluorescence images were processed and analyzed using ImageJ (version 1.53c) (https://fiji.sc/).

Immunofluorescence of *Drosophila* ovaries and testes

Three-day-old females and one-day-old males were selected to dissect ovaries and testes. The tissues were fixed for 20 min in 4% paraformaldehyde at room temperature. Next, tissue samples were washed with PBTT (1 × PBS with 0.1% Tween 20 and 0.3% Triton X-100) for 3 times (15 min each time), and blocked with 3% BSA for 30 min. Subsequently, ovaries or testes were incubated with primary antibodies overnight at 4°C. The following day, samples were first incubated with 1 μg/mL DAPI for 15 min and then washed with PBTT for 4 times (20 min each time). Secondary antibodies were added to samples and incubated for 2 h at room temperature. After three 20 min washes with PBTT and three 5 min washes with PBS, slides can be made with the anti-fade mounting medium. The acquisition of microscopy images was done with confocal microscope (ZEISS LSM880). The following antibodies were used in this study: Rabbit anti-H3Ser10P (1:300, EMD Millipore, 06–570); Mouse anti-SXL (1:100, Developmental Studies Hybridoma Bank, M18-s); Mouse anti-Ubiquitin (1:100, Santa Cruz Biotechnology, sc-8017); Alexa Fluor 488 and 594 (1:200, Jackson Immuno Research).

QUANTIFICATION AND STATISTICAL ANALYSIS

RNA sequencing data were first aligned to the *Drosophila* reference genome using HISAT2, followed by transcript assembly and quantification via StringTie. More details on the analysis can be found in method details. DESeq2 was used for differential expression analysis,

and transcripts with adjusted p value <0.1 were considered to be differentially expressed. Kolmogorov-Smirnov tests were used to compare the difference of ratio distributions between two groups, and a p value <0.05 were considered significant. Fisher's exact tests were used for enrichment analysis. The threshold of co-expressed lncRNA-mRNA pairs was set as absolute values of correlation coefficient >0.95 and p value <0.05. Co-located lncRNA-mRNA pairs were defined as being within 10 kb upstream and downstream. ImageJ was used to analyze the fluorescence intensity of photographs taken by confocal microscopy. Two tailed Student's *t*-tests were performed to compare the relative fluorescence intensity, and the level of significance were shown in figure legends. All statistical tests and plots were performed with R software.

iScience, Volume *26*

Supplemental information

Characteristics and expression of lncRNA and transposable elements in *Drosophila* aneuploidy

Shuai Zhang, Ruixue Wang, Xilin Zhu, Ludan Zhang, Xinyu Liu, and Lin Sun

iScience, Volume *26*

Supplemental information

Characteristics and expression of lncRNA and transposable elements in *Drosophila* aneuploidy

Shuai Zhang, Ruixue Wang, Xilin Zhu, Ludan Zhang, Xinyu Liu, and Lin Sun

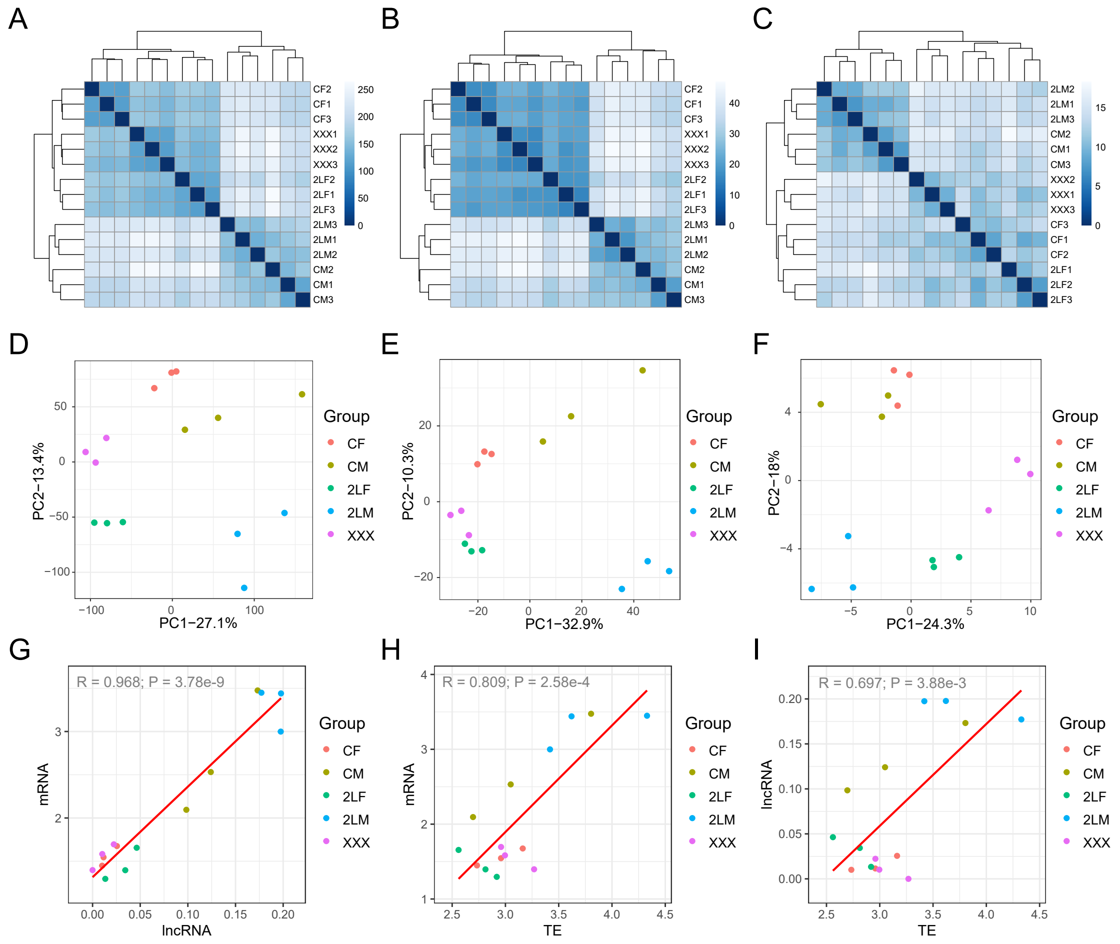

Figure S1, Related to Figure 1. Quality control of samples by different transcript types

(A-C) Heatmaps of distances between samples based on protein-coding genes (A), long noncoding RNAs (lncRNAs) (B), and transposable elements (TEs) (C).

(D-F) PCA plots of samples based on protein-coding genes (D), lncRNAs (E), and TEs (F).

(G-I) Correlation of the expression of different types of transcripts among samples. The Pearson's correlation coefficients and p-values of the medians of expression levels (CPM) for two types of transcripts among samples are shown in the upper left corner of the plots.

CF, wildtype female control; CM, wildtype male control; 2LF, trisomy 2L female; 2LM, trisomy 2L male; XXX, metafemale.

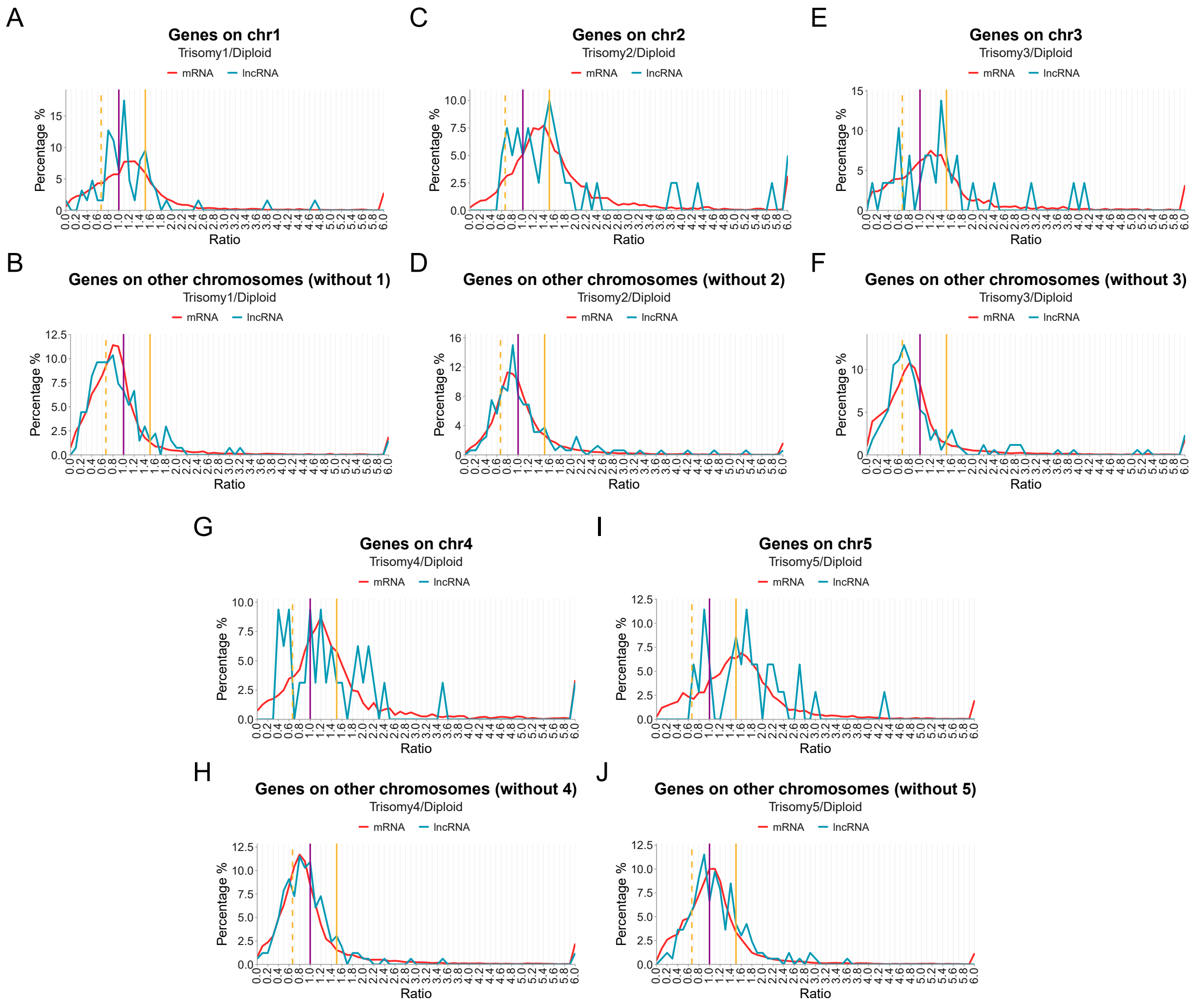

Figure S2, Related to Figure 2. Ratio Distributions of lncRNA and mRNA in aneuploid *Arabidopsis*

(A,B) Ratio distributions of the expression levels of lncRNA and mRNA on chromosome 1 (A) and other chromosomes (B) in trisomy 1 *Arabidopsis*.

(C,D) Ratio distributions of the expression levels of lncRNA and mRNA on chromosome 2 (C) and other chromosomes (D) in trisomy 2 *Arabidopsis*.

(E,F) Ratio distributions of the expression levels of lncRNA and mRNA on chromosome 3 (E) and other chromosomes (F) in trisomy 3 *Arabidopsis*.

(G,H) Ratio distributions of the expression levels of lncRNA and mRNA on chromosome 4 (G) and other chromosomes (H) in trisomy 4 *Arabidopsis*.

(I,J) Ratio distributions of the expression levels of lncRNA and mRNA on chromosome 5 (I) and other chromosomes (J) in trisomy 5 *Arabidopsis*.

The vertical purple solid line represents the ratio of 1.00, the vertical yellow solid line represents the ratio of 1.50, and the vertical yellow dashed line shows the ratio of 0.67. The ratio distributions were generated using ERCC-normalized RPKM data downloaded from GSE79676, and the percentages of frequencies were plotted in bins of 0.1.

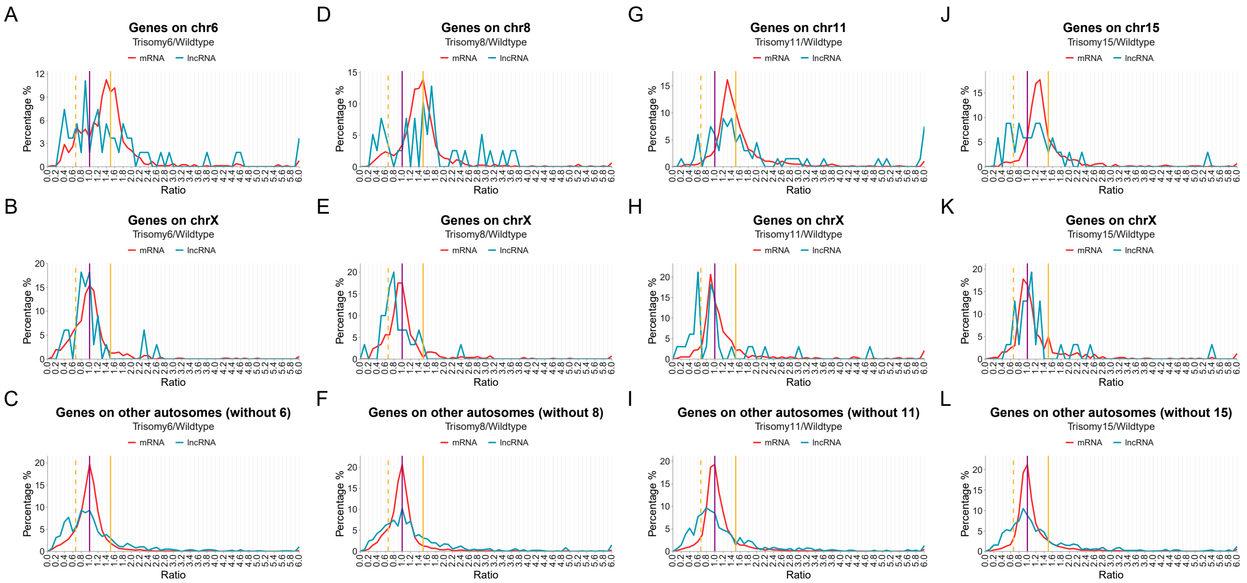

Figure S3, Related to Figure 2. Ratio Distributions of lncRNA and mRNA in aneuploid embryonic stem cells in mouse

(A-C) Ratio distributions of the expression levels of lncRNA and mRNA on chromosome 6 (A), chromosome X (B), and other autosomes (C) in trisomy 6 mouse embryonic stem cells.

(D-F) Ratio distributions of the expression levels of lncRNA and mRNA on chromosome 8 (D), chromosome X (E), and other autosomes (F) in trisomy 8 mouse embryonic stem cells.

(G-I) Ratio distributions of the expression levels of lncRNA and mRNA on chromosome 11 (G), chromosome X (H), and other autosomes (I) in trisomy 11 mouse embryonic stem cells.

(J-L) Ratio distributions of the expression levels of lncRNA and mRNA on chromosome 15 (J), chromosome X (K), and other autosomes (L) in trisomy 15 mouse embryonic stem cells.

The vertical purple solid line represents the ratio of 1.00, the vertical yellow solid line represents the ratio of 1.50, and the vertical yellow dashed line shows the ratio of 0.67. The ratio distributions were generated using CPM data downloaded from GSE179435, and the percentages of frequencies were plotted in bins of 0.1.

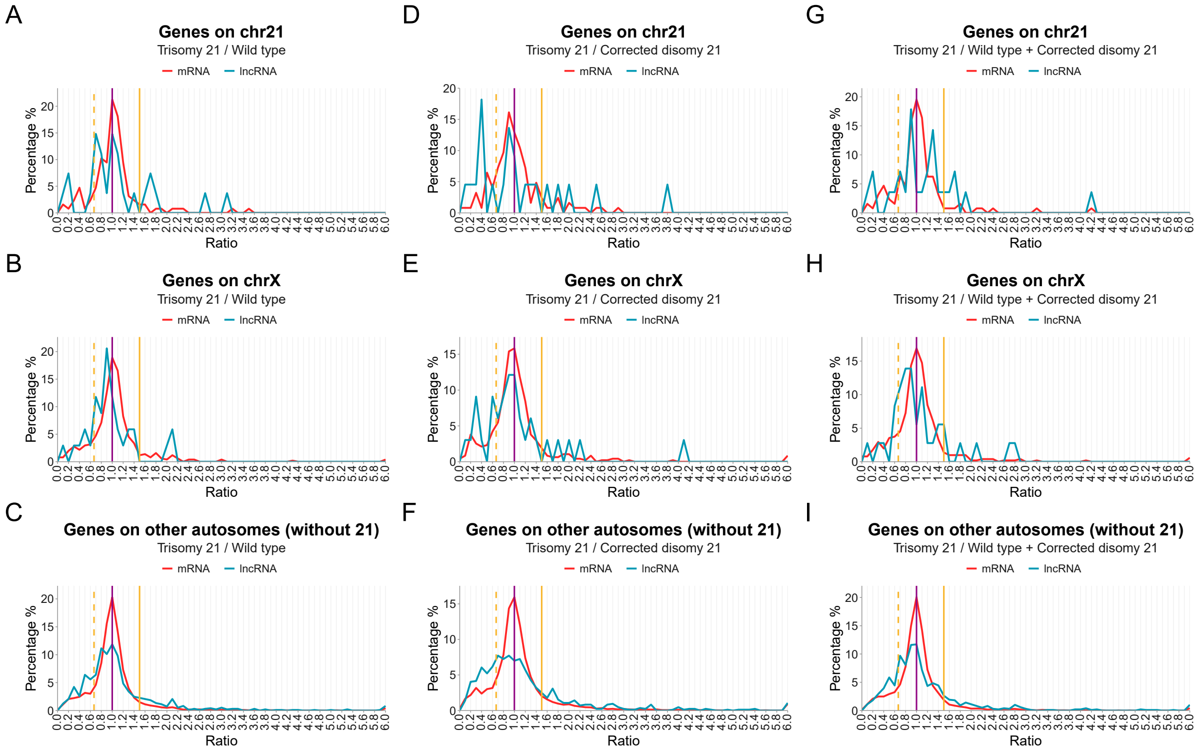

Figure S4, Related to Figure 2. Ratio Distributions of lncRNA and mRNA in induced-pluripotent stem-derived vascular endothelial cells (iPSC-derived ECs) in Down syndrome (human trisomy 21)

(A-C) Ratio distributions of the expression levels of lncRNA and mRNA on chromosome 21 (A), chromosome X (B), and other autosomes (C) in trisomy 21 iPSC-derived ECs compared with wildtype cells.

(D-F) Ratio distributions of the expression levels of lncRNA and mRNA on chromosome 21 (D), chromosome X (E), and other autosomes (F) in trisomy 21 iPSC-derived ECs compared with isogenic corrected disomy 21 cells.

(G-I) Ratio distributions of the expression levels of lncRNA and mRNA on chromosome 21 (G), chromosome X (H), and other autosomes (I) in trisomy 21 iPSC-derived ECs compared with wildtype and corrected disomy 21 cells.

The vertical purple solid line represents the ratio of 1.00, the vertical yellow solid line represents the ratio of 1.50, and the vertical yellow dashed line shows the ratio of 0.67. The ratio distributions were generated using FPKM data downloaded from GSE203257, and the percentages of frequencies were plotted in bins of 0.1.

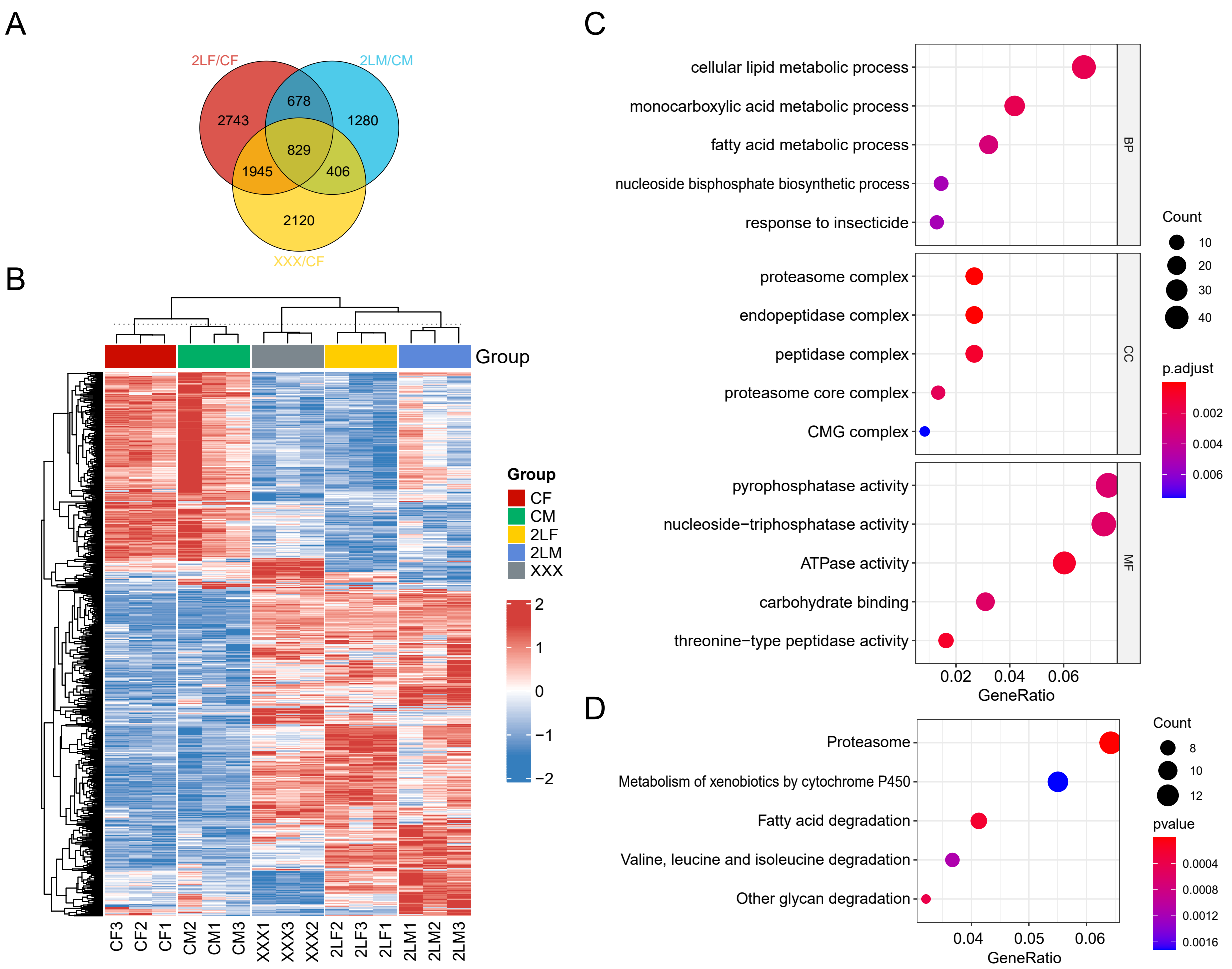

Figure S5, Related to Figure 3. Differential expression analysis of mRNA in aneuploid *Drosophila*

(A) Venn diagram of the number of differentially expressed mRNA (DE-mRNA) in trisomy 2L female, trisomy 2L male, and metafemale.

(B) Clustering heatmap of mRNAs differentially expressed in all three aneuploidies.

(C) Top 5 enriched GO terms in biological process (BP), cell component (CC), and molecular function (MF) of DE-mRNA in three aneuploidies.

(D) Top 5 enriched KEGG pathways of DE-mRNA in three aneuploidies.

CF, wildtype female control; CM, wildtype male control; 2LF, trisomy 2L female; 2LM, trisomy 2L male; XXX, metafemale.

A

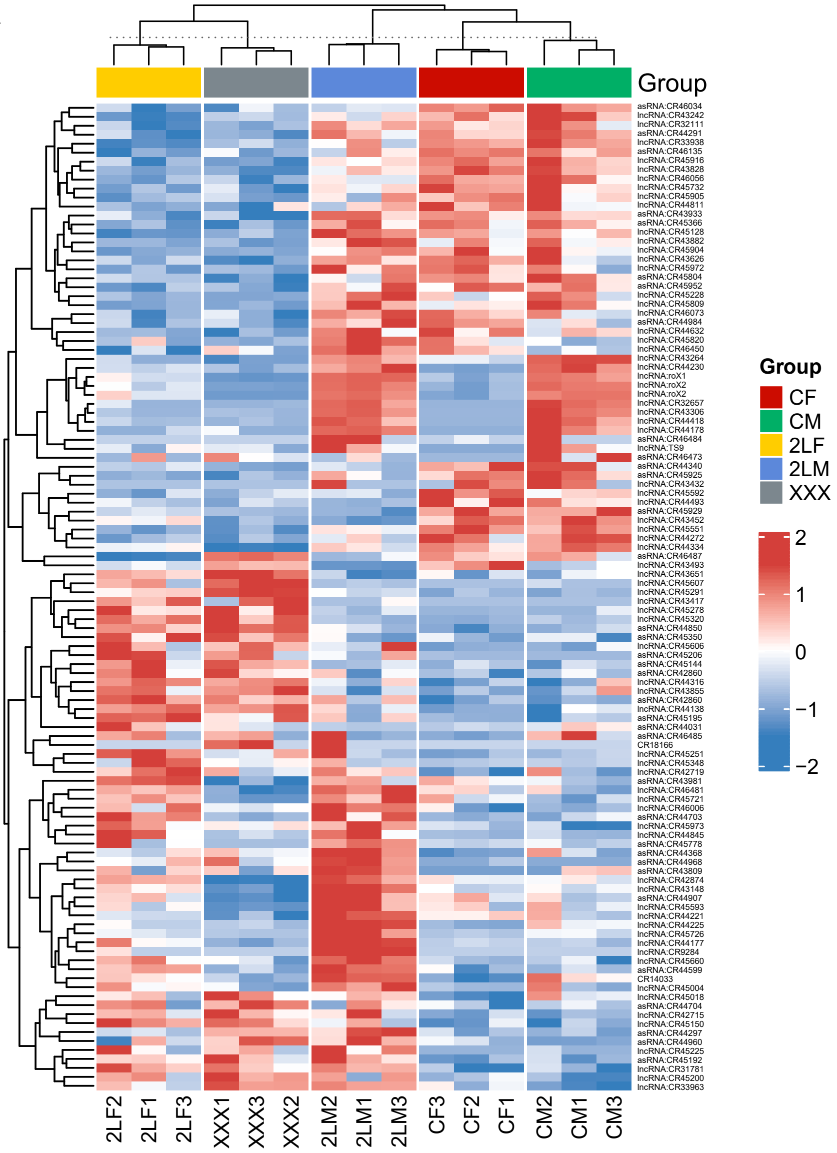

Figure S6, Related to Figure 3. LncRNAs that are significantly differentially expressed in at least two groups of comparisons

(A) Clustering heatmap of lncRNAs differentially expressed in at least two groups of comparisons of trisomy 2L female, trisomy 2L male, and metafemale to their respective controls. CF, wildtype female control; CM, wildtype male control; 2LF, trisomy 2L female; 2LM, trisomy 2L male; XXX, metafemale.

A

lncRNA	predicted functions
lncRNA:roX2	oxidative phosphorylation; mitophagy; biosynthesis of amino acids; peroxisome
lncRNA:CR43306	inorganic ion homeostasis; aerobic respiration; cilium assembly; cilium movement; sperm axoneme assembly; organelle assembly; axoneme assembly; plasma membrane bounded cell projection assembly; mitophagy; biosynthesis of amino acids; peroxisome
lncRNA:CR44178	metal ion homeostasis; aerobic respiration; cilium assembly; cilium movement; organelle assembly; mitophagy; biosynthesis of amino acids
lncRNA:CR32657	inorganic ion homeostasis; aerobic respiration; cilium assembly; cilium movement; sperm axoneme assembly; organelle assembly; mitophagy; biosynthesis of amino acids; peroxisome
lncRNA:CR33938	ubiquitin-dependent protein catabolic process; proteasome assembly; cell fate specification; leg disc proximal/distal pattern formation; renal tubule development; Malpighian tubule development; transcription initiation from RNA polymerase II promoter; histone H4 acetylation; cellular response to virus; regulation of smoothened signaling pathway; proteasome; protein processing in endoplasmic reticulum
lncRNA:roX1	oxidative phosphorylation; mitophagy; biosynthesis of amino acids; peroxisome
lncRNA:CR44418	mitophagy; biosynthesis of amino acids
lncRNA:CR43828	lysosome
lncRNA:CR44225	regulation of actin polymerization or depolymerization; receptor metabolic process; supramolecular fiber organization; glutamine family amino acid biosynthetic process; receptor clustering
lncRNA:CR45916	glycerophospholipid metabolism
lncRNA:CR9284	regulation of hydrolase activity; tyrosine metabolic process; apoptotic mitochondrial changes
asRNA:CR44850	humoral immune response; positive regulation of antibacterial peptide biosynthetic process; positive regulation of Toll signaling pathway; negative regulation of endopeptidase activity; negative regulation of proteolysis; mitochondrial electron transport, cytochrome c to oxygen
lncRNA:CR45809	attachment of spindle microtubules to kinetochore; regulation of cell cycle; protein phosphorylation; negative regulation of BMP signaling pathway; membrane organization; sperm axoneme assembly; male gamete generation

B

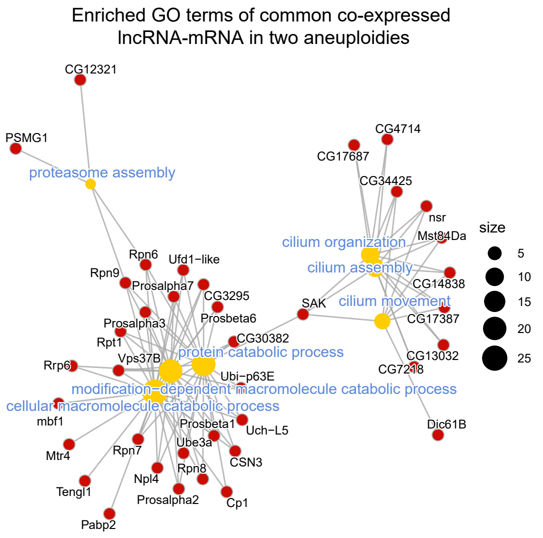

C

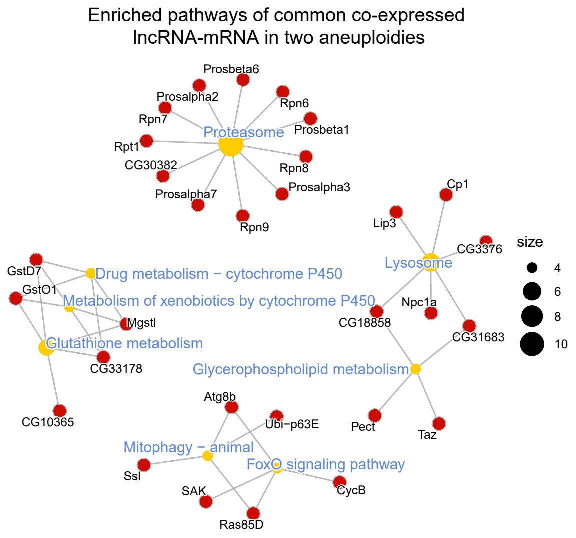

Figure S7, Related to Figure 4. The function of lncRNAs predicted based on lncRNA-mRNA co-expression network

(A) The predicted functions of several lncRNAs with high degree according to the enrichment analysis of their co-expressed mRNAs.

(B) Enriched functions of lncRNA-mRNA co-expression network and related genes.

(C) Enriched pathways of lncRNA-mRNA co-expression network and related genes.

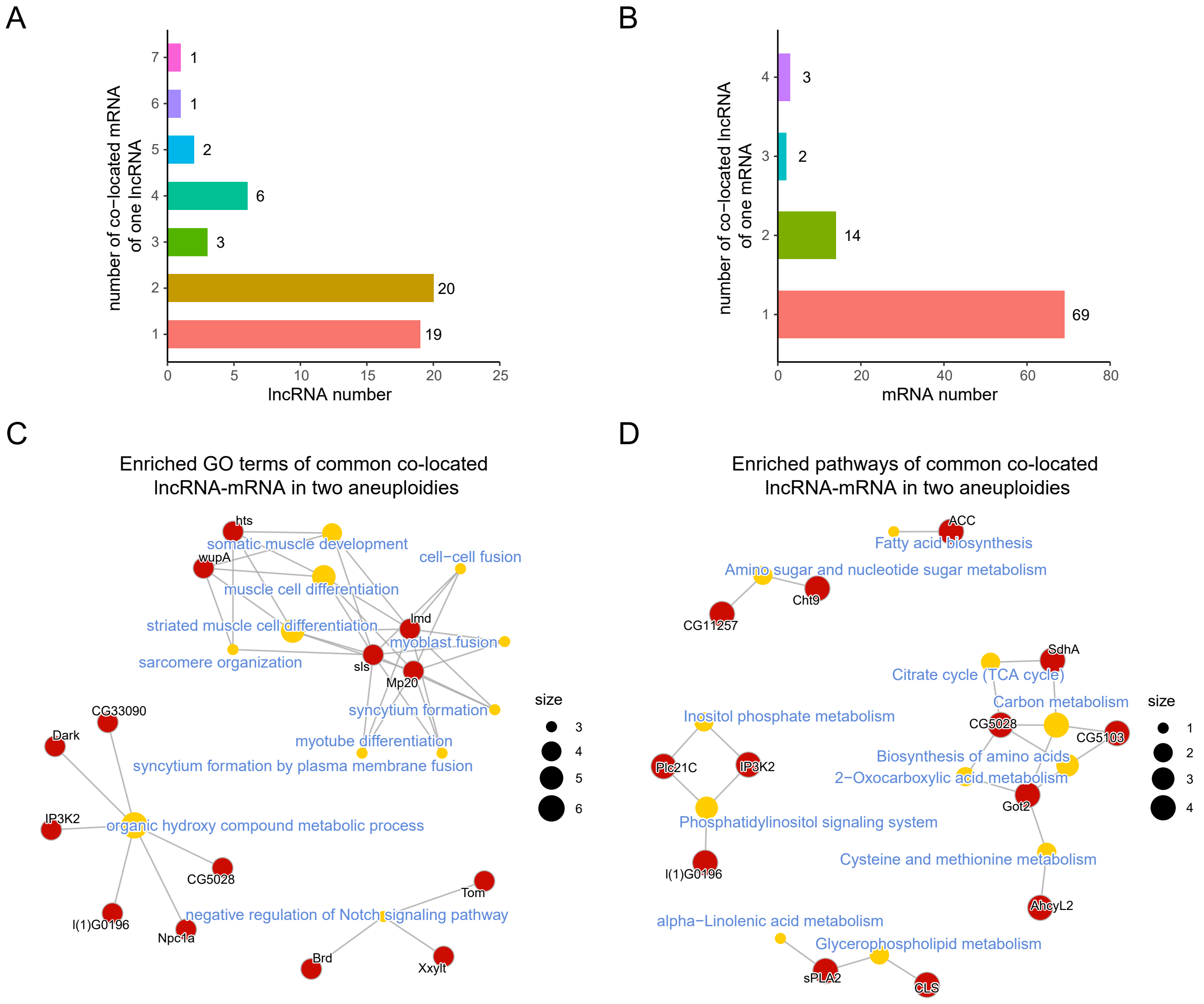

Figure S8, Related to Figure 4. Co-located lncRNA and mRNA, and their enrichment analysis

(A) The number of co-located mRNA owned by per lncRNA.

(B) The number of co-located lncRNA owned by per mRNA.

(C) Enriched functions of lncRNA-mRNA co-localization network and related genes.

(D) Enriched pathways of lncRNA-mRNA co-localization network and related genes.

A

TE	predicted functions
HMS-Beagle	ubiquitin-dependent protein catabolic process; proteasome assembly; response to heat; response to external biotic stimulus; Hippo signaling pathway; apoptosis
accord	organophosphate metabolic process; regulation of ATP metabolic process; purine nucleotide biosynthetic process; oxidative phosphorylation; drug metabolism
Burdock	cellular metal ion homeostasis; regulation of lipid storage; organic acid transmembrane transport
Juan	purine ribonucleoside monophosphate biosynthetic process; ocellus pigment metabolic process; biosynthesis of cofactors
Max	negative regulation of peptide secretion; regulation of calcium-mediated signaling; cellular response to starvation; response to carbohydrate; oxidative phosphorylation; drug metabolism; phagosome

B C

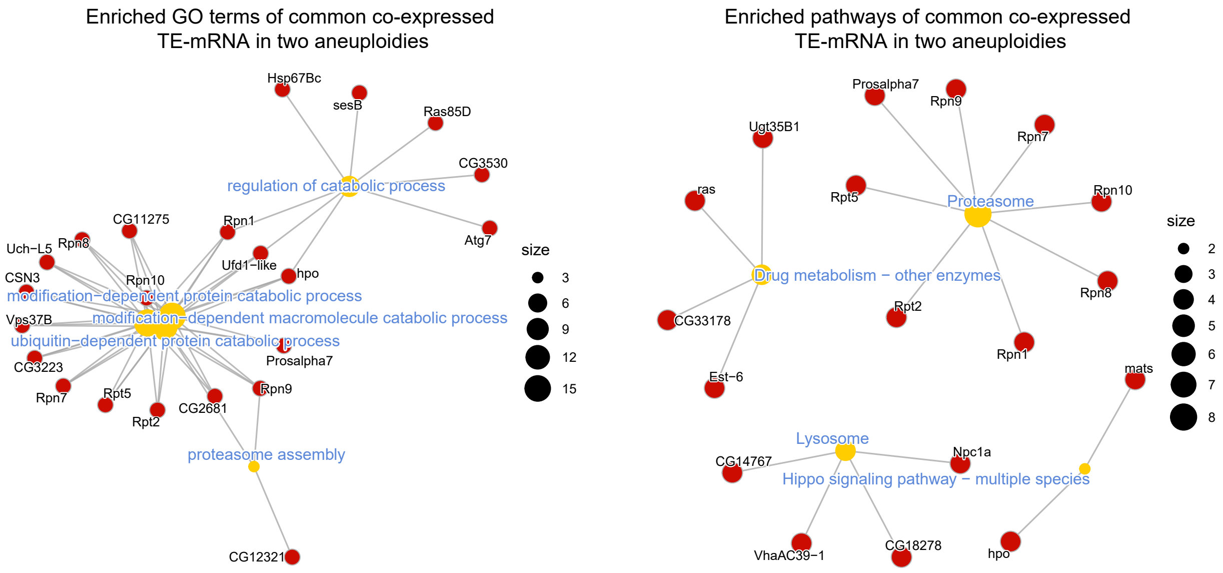

Figure S9, Related to Figure 5. The function of TE families predicted based on TE-mRNA co-expression network

(A) The predicted functions of several TE families with high degree according to the enrichment analysis of their co-expressed mRNAs.

(B) Enriched functions of TE-mRNA co-expression network and related genes.

(C) Enriched pathways of TE-mRNA co-expression network and related genes.

A

Primer name	Sequence
CG3295-F-T3	TGTTGGGAAATCACTCCCAATTAACTGGAGCTAACAATGGACAG
CG3295-R-T7	GTAATACGACTCACTATAGGGAGACCACAGTAGGGGCACTTCAGTATG
HmgD-F-T3	TGTTGGGAAATCACTCCCAATTAACCACCCTAATCCATCACCAA
HmgD-R-T7	GTAATACGACTCACTATAGGGAGACCACCAACGCACATCCTGCTAATC
tsh-F-T3	TGTTGGGAAATCACTCCCAATTAACTCCGAAAGCATAGTTCAGG
tsh-R-T7	GTAATACGACTCACTATAGGGAGACCACCAACCTTGTCTGAGGACTTG
dap-F-T3	TGTTGGGAAATCACTCCCAATTAAGACCGCAAGCTAAATGGAAT
dap-R-T7	GTAATACGACTCACTATAGGGAGACCACAAGTAATGCGGATGTGTTGG
Tehao-F-T3	TGTTGGGAAATCACTCCCAATTAATTGCGTCTGAACAAGAACTC
Tehao-R-T7	GTAATACGACTCACTATAGGGAGACCACGTAGGTCTTGTCCTTGTCCA
Su(var)205-F-T3	TGTTGGGAAATCACTCCCAATTAATCAAAACGTACCACAGATGC
Su(var)205-R-T7	GTAATACGACTCACTATAGGGAGACCACTACAGACACTGACTCTGGAC
ifc-F-T3	TGTTGGGAAATCACTCCCAATTAATTATTAACCCGAAGCCACCT
ifc-R-T7	GTAATACGACTCACTATAGGGAGACCACGTCCGAATGCCCCTTATCTA
nonA-l-F-T3	TGTTGGGAAATCACTCCCAATTAAGACGCAACCGCTTATATGTT
nonA-l-R-T7	GTAATACGACTCACTATAGGGAGACCACCGTCGCTATTATCTGGACCT
Atg8b-F-T3	TGTTGGGAAATCACTCCCAATTAAGCAGCTACATAAGACCTTCG
Atg8b-R-T7	GTAATACGACTCACTATAGGGAGACCACTAGTCCTTGTCGAAGTGCTC
lncRNA:CR33938-F-T3	TGTTGGGAAATCACTCCCAATTAAAAAACGTATGGACTCGGCTA
lncRNA:CR33938-R-T7	GTAATACGACTCACTATAGGGAGACCACAAATTTTGTCCAAAGGGCCG
lncRNA:CR45916-F-T3	TGTTGGGAAATCACTCCCAATTAAAATGCGATGGGAGTAAGAGT
lncRNA:CR45916-R-T7	GTAATACGACTCACTATAGGGAGACCACAGGGGGTAAAACAACTCCAT
asRNA:CR42860-F-T3	TGTTGGGAAATCACTCCCAATTAAGATGACTGAACAGCCGTAAC
asRNA:CR42860-R-T7	GTAATACGACTCACTATAGGGAGACCACAGGCATACGTGGATGGTTTA
lncRNA:CR43148-F-T3	TGTTGGGAAATCACTCCCAATTAAAAACTGTAAGGAAGGTGGCT
lncRNA:CR43148-R-T7	GTAATACGACTCACTATAGGGAGACCACGGCTTGTAGGAGTGGAAATC
lncRNA:CR44178-F-T3	TGTTGGGAAATCACTCCCAATTAAAGGGTATTGAAAGAGGGGTG
lncRNA:CR44178-R-T7	GTAATACGACTCACTATAGGGAGACCACGTCAACAGTTTCCACGTCTT
lncRNA:CR44418-F-T3	TGTTGGGAAATCACTCCCAATTAAAGTTGGAACAGGACGAGTAA
lncRNA:CR44418-R-T7	GTAATACGACTCACTATAGGGAGACCACTATTTGCCCGAAGTCAACTC
HMS-Beagle-F-T3	TGTTGGGAAATCACTCCCAATTAAGAATGTAGAGTGGGGAGTGG
HMS-Beagle-R-T7	GTAATACGACTCACTATAGGGAGACCACTATTGATGGAGAAGGCGGTC

B

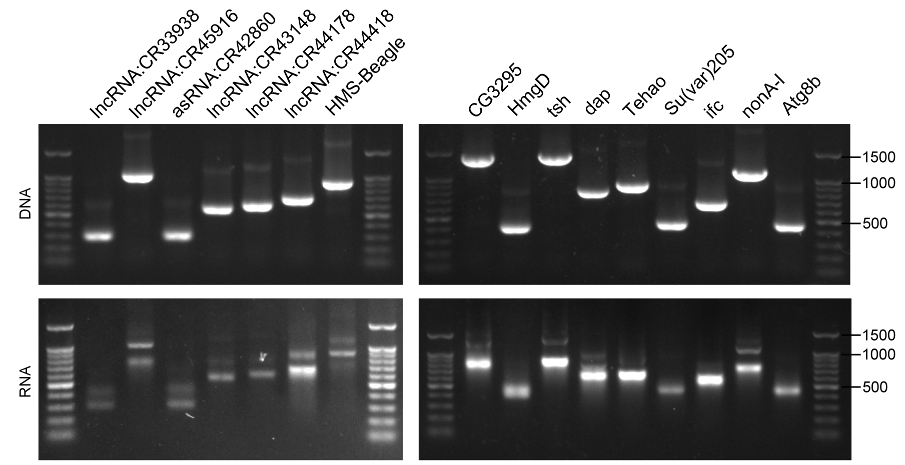

Figure S10, Related to Figure 6. Probe primers designed for candidate genes and their validation

(A) The sequences of probe primers.

(B) The size of the products of the primers after PCR amplification and *in vitro* transcription was tested by agarose gel electrophoresis.
